# Plant metabolites: potential treatments for ischemic acute kidney injury

**DOI:** 10.3389/fphar.2026.1891651

**Published:** 2026-08-14

**Authors:** Yan Li, Wenting Zhang, Shengpeng Zhang, Shaozhen Wang, Yuewu Xie, Qiuyue Lv

**Affiliations:** School of Pharmacy, Wannan Medical University, Wuhu, China

**Keywords:** botanical drugs, ischemic acute kidney injury, phytochemicals, plant metabolites, renoprotection

## Abstract

Ischemia-reperfusion injury is a leading cause of acute kidney injury (AKI), which is characterized by high morbidity and mortality, as well as a substantial risk of progression to chronic kidney disease (CKD). Although supportive care has improved considerably, effective disease-modifying therapies are still lacking. Therefore, the development of new therapeutic drugs for ischemic AKI is crucial. Active metabolites derived from medicinal plants, owing to their low toxicity and multi-target characteristics, are promising candidates for ischemic AKI therapy. This study reviews the pathogenesis of ischemic AKI from multiple dimensions, including metabolic disturbances, oxidative stress, inflammation, endoplasmic reticulum stress, mitochondrial dysfunction, and several forms of regulated cell death (e.g., ferroptosis, apoptosis, pyroptosis). It also summarizes plant metabolites (polyphenols, phenylpropanoids, terpenoids, saponins, alkaloids, and extracts) that can ameliorate ischemic AKI by targeting the aforementioned mechanisms. These metabolites exert their protective effects by modulating key signaling pathways (e.g., Nrf2/HO-1, NF-κB, PI3K/Akt). However, further mechanistic studies, standardization, and well-designed clinical trials are essential for their use and to ensure their integration into clinical practice as effective therapeutic options.

## Graphical Abstract

**Figure:**
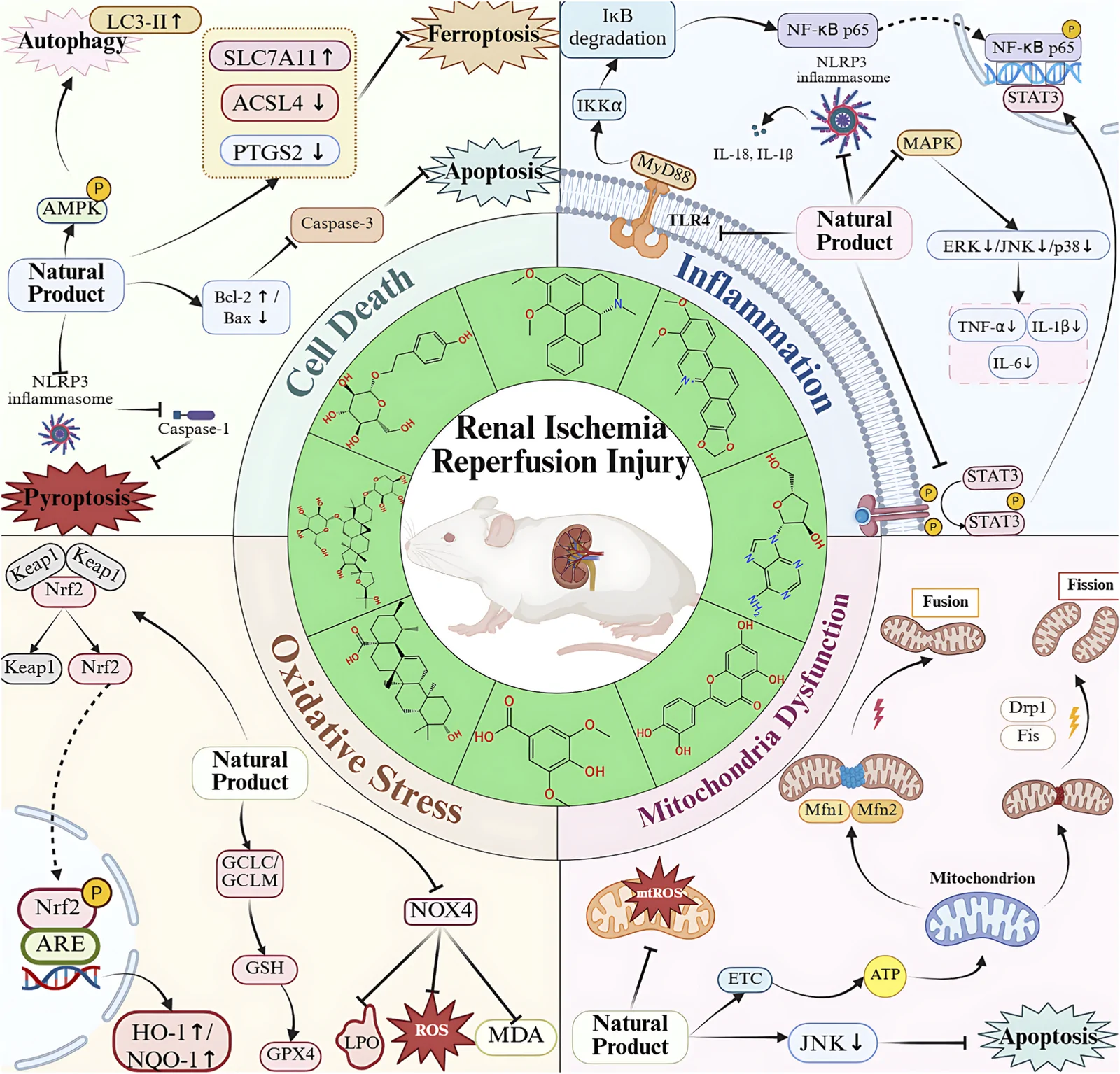

## 1 Introduction

Ischemia-reperfusion (I/R) injury represents one of the most prevalent and clinically significant causes of AKI, which is associated with substantial morbidity, mortality, and long-term renal dysfunction worldwide (Norgard and Svenningsen, 2023). Clinically, ischemic AKI commonly occurs in settings such as kidney transplantation, partial nephrectomy, major cardiovascular surgery, sepsis, and hemorrhagic shock (Chen et al., 2024). Current clinical management remains largely supportive, focusing on hemodynamic optimization and renal replacement therapy in severe cases (Barbar et al., 2025). Although advances in surgical techniques and perioperative management have improved patient outcomes, no specific pharmacological therapy has yet been approved to effectively prevent or treat ischemic AKI (Si et al., 2026). Consequently, identifying novel therapeutic strategies capable of mitigating ischemic AKI remains an urgent unmet clinical need.

The pathophysiology of ischemic AKI is highly complex and involves a dynamic interplay of metabolic disturbance, oxidative stress, inflammation, endothelial dysfunction, and various forms of regulated cell death (Ostermann et al., 2025; Yan et al., 2025). During the ischemic phase, reduced oxygen and nutrient supply leads to adenosine triphosphate (ATP) depletion, disruption of ion homeostasis, and mitochondrial dysfunction (Christou et al., 2026). Upon reperfusion, the abrupt restoration of oxygen paradoxically triggers excessive production of reactive oxygen species (ROS), resulting in lipid peroxidation, protein oxidation, and DNA damage (Reid and Scholey, 2021). Concurrently, activation of innate immune responses promotes the release of proinflammatory cytokines and chemokines, facilitating leukocyte infiltration and amplifying tissue injury. Emerging evidence further indicates that regulated cell death pathways, including apoptosis, necroptosis, ferroptosis, and pyroptosis, play critical roles in propagating renal damage (Li C. et al., 2024). These interconnected processes form a vicious cycle that ultimately culminates in tubular epithelial cell injury, microvascular rarefaction, and impaired renal function.

Importantly, the multifactorial and network-based characteristics of ischemic AKI pose considerable challenges for effective therapeutic intervention (Xu X. et al., 2025). Numerous experimental approaches that focus on individual molecules or isolated signaling pathways have exhibited limited success in translating to clinical practice (Zhang et al., 2024). Consequently, therapeutic agents that simultaneously modulate multiple interconnected pathways may provide enhanced protective effects compared to interventions targeting a single pathway. This holistic approach could potentially improve the efficacy of treatment strategies for ischemic AKI, ultimately leading to better clinical outcomes (Song et al., 2025).

Plant metabolites have garnered increasing attention as a critical resource in drug research and development (Jiao et al., 2025). Derived from medicinal plants, these metabolites encompass a variety of bioactive metabolites, including flavonoids, alkaloids, terpenoids, phenolic acids, and saponins, which demonstrate extensive pharmacological activities (Shyam et al., 2025; Zhou et al., 2023). A multitude of preclinical investigations have shown that these metabolites mitigate renal dysfunction and histological damage in ischemic AKI models (Castaneda et al., 2023; Wang et al., 2024a). From a mechanistic standpoint, plant metabolites often engage endogenous antioxidant defense mechanisms, notably through the activation of the nuclear factor erythroid 2-related factor 2/heme oxygenase-1 (Nrf2/HO-1) signaling pathway (Tian et al., 2025). They also enhance mitochondrial resilience, suppress nuclear receptor κB (NF-κB)-mediated inflammatory responses, inhibit NOD-like receptor family pyrin domain-containing 3 (NLRP3) inflammasome activation, and modulate regulated cell death pathways, including apoptosis and ferroptosis (Fahim et al., 2026). Furthermore, viewed through the lens of systems pharmacology, the multi-target regulatory effects of plant metabolites are particularly well-suited to address the intricate pathogenesis of ischemic AKI.

This review aims to systematically summarize the renoprotective effects of plant metabolites in ischemic AKI, assess the methodological strengths and limitations of existing studies, and discuss prospective directions for translational applications, thereby deepening understanding of the therapeutic landscape and informing future research aimed at developing effective multi-target strategies for ischemic AKI.

## 2 Methods

This work represents a narrative review rather than a formal systematic review. The databases searched included PubMed, Web of Science, and Scopus, covering the past decade. Combinations of keywords used included “renal ischemia-reperfusion injury,” “ischemic acute kidney injury,” “plant metabolites,” “phytochemicals,” “plant extracts,” “botanical drugs,” “flavonoids,” “alkaloids,” “terpenoids,” “polyphenols,” “saponins,” “polysaccharides,” “oxidative stress,” “inflammation,” “mitochondrial dysfunction,” “apoptosis,” “autophagy,” “ferroptosis,” and “pyroptosis.”

Studies were included if they met the following criteria: peer-reviewed original research published in English; investigations of renal ischemia–reperfusion injury; *in vivo* animal models or *in vitro* hypoxia/reoxygenation (H/R) models relevant to renal injury; evaluation of plant metabolites, extracts, or botanical drugs as the main intervention. Studies were excluded if they were reviews, editorials, commentaries, letters, case reports, theses, or conference abstracts without sufficient methodological information; studies not published in English; or represented duplicate publications or overlapping datasets.

Data extraction was performed using a standardized form and included first author, publication year, experimental model, animal species or cell type, ischemia and reperfusion duration, plant metabolite, dose, route and timing of administration, comparator group and reported molecular mechanisms. Included studies were categorized according to the class of plant metabolites, including phenylpropanoids, polyphenols, alkaloids, terpenoids, saponins, and other naturally derived bioactive metabolites. Mechanistic findings were grouped by key processes involved in renal ischemia–reperfusion injury, including oxidative stress, inflammation, mitochondrial dysfunction, apoptosis, autophagy, ferroptosis, pyroptosis, endothelial dysfunction, and immune regulation.

## 3 Pathophysiology of ischemic AKI

Ischemic AKI involves a complex interplay among metabolic disturbances, oxidative stress, inflammatory responses, endoplasmic reticulum stress, mitochondrial dysfunction, and several forms of regulated cell death (e.g., ferroptosis, apoptosis, pyroptosis). This cascade of events engenders a detrimental cycle that intensifies renal damage.

### 3.1 Metabolic disturbance and oxidative stress

**FIGURE 1:**
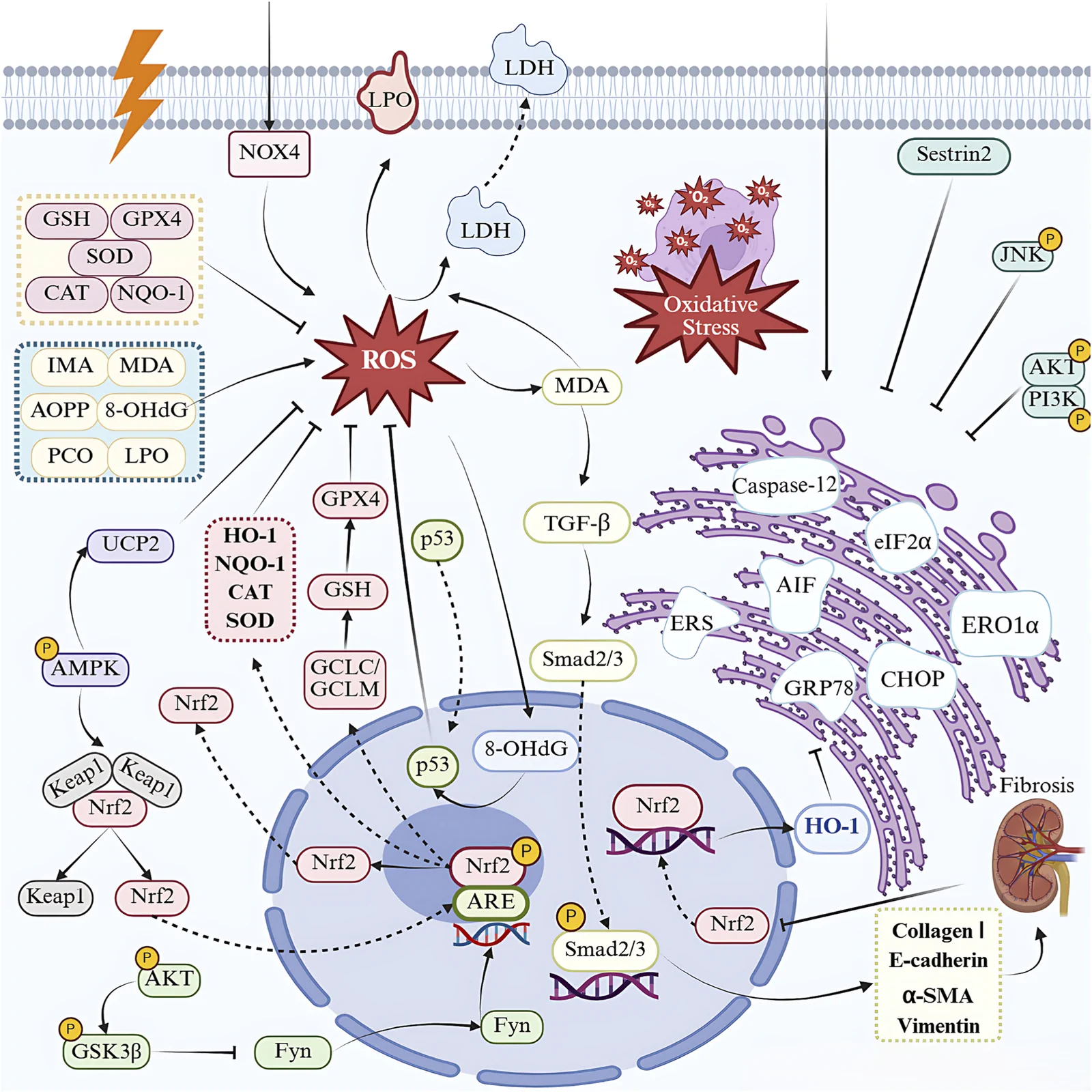
Oxidative stress-related mechanisms. This figure summarizes ROS overproduction, impaired antioxidant defenses, oxidative macromolecular damage, lipid metabolic disturbance, inflammation, apoptosis, and Nrf2/HO-1-mediated antioxidant regulation in ischemic AKI. Natural metabolites may attenuate oxidative injury by reducing ROS generation, restoring antioxidant capacity, suppressing pro-inflammatory and pro-apoptotic signaling, and activating the Nrf2/HO-1 axis. Solid arrows indicate activation, dashed arrows indirect regulation, T-bar/flat-head arrows inhibition, upward/downward arrows increased/decreased activity, and starburst symbols pathway activation.

Ischemic AKI involves metabolic dysregulation and redox imbalance at multiple levels (Granata et al., 2022; Pan and Zhu, 2026). Among these pathological processes, oxidative stress is widely recognized as a central driver of renal injury (Figure 1). During the ischemic phase, oxygen deprivation disrupts mitochondrial oxidative phosphorylation, leading to ATP depletion and impaired electron transport chain function (Li et al., 2025). This metabolic collapse promotes xanthine oxidoreductase activity and perturbs nitric oxide synthase (NOS) signaling, increasing nitric oxide (NO) and superoxide generation (Granata et al., 2022). Early oxidative injury is evidenced by shifts in redox-sensitive biomarkers, including oxidative stress indices and thiol oxidation markers, such as reduced protein sulfhydryl content, which reflects thiol oxidation and redox imbalance (Ozhan et al., 2026). Upon reperfusion, abrupt oxygen reintroduction triggers a burst of ROS production, amplifying oxidative injury. Excessive ROS attacks cellular macromolecules, leading to lipid peroxidation, protein and DNA oxidation (Chouchani et al., 2014).

In parallel with excessive ROS production, endogenous antioxidant defense systems are significantly impaired in ischemic AKI. The activities of key antioxidant enzymes—superoxide dismutase (SOD), catalase (CAT), and glutathione peroxidase (GPX) are markedly reduced, accompanied by depletion of reduced glutathione (GSH), collectively leading to diminished overall antioxidant capacity (Piko et al., 2023). Dysregulation of glutathione synthesis machinery, such as glutamate-cysteine ligase (GCLC), further aggravates redox imbalance (Li Y. et al., 2024). The weakened antioxidant system contributes to ROS accumulation and subsequent downstream oxidative damage. Meanwhile, prooxidant inflammatory mechanisms intensify, with increased myeloperoxidase (MPO) activity associated with neutrophil infiltration and oxidative burst.

Beyond direct oxidative damage, ROS also function as signaling mediators that amplify injury cascades (Xing et al., 2022). Enhanced Fas/Fas ligand signaling reinforces apoptotic programs and propagates downstream stress signaling, contributing to feedforward injury loops in ischemic AKI (Ko et al., 2011). Oxidative stress is also closely linked to lipid metabolic reprogramming. It promotes lipid uptake and lipogenesis through cluster of differentiation 36 and stearoyl-CoA desaturase 1/fatty acid synthase, while inhibiting fatty acid oxidation via carnitine palmitoyltransferase 1 (CPT1) and peroxisome proliferator-activated receptor alpha (PPARα), thereby promoting lipotoxicity (Lee et al., 2024). Excess lipid peroxidation compromises membrane permeability, consistent with lactate dehydrogenase (LDH) leakage as a readout of cellular injury (Baryla et al., 2024).

Importantly, the Nrf2/HO-1 axis plays a pivotal role in maintaining redox homeostasis during ischemic AKI. Impaired Nrf2 activation exacerbates oxidative injury, whereas pharmacological or genetic activation confers renoprotection (Bondi et al., 2024). Given its central role in redox regulation, oxidative stress represents both a cornerstone mechanism in ischemic AKI and a major therapeutic target for antioxidant natural metabolites (Tian et al., 2025). Therefore, targeting oxidative stress and restoring redox balance has emerged as a primary strategy through which numerous natural metabolites exert renoprotective effects in ischemic AKI models.

### 3.2 Inflammatory cascade

**FIGURE 2:**
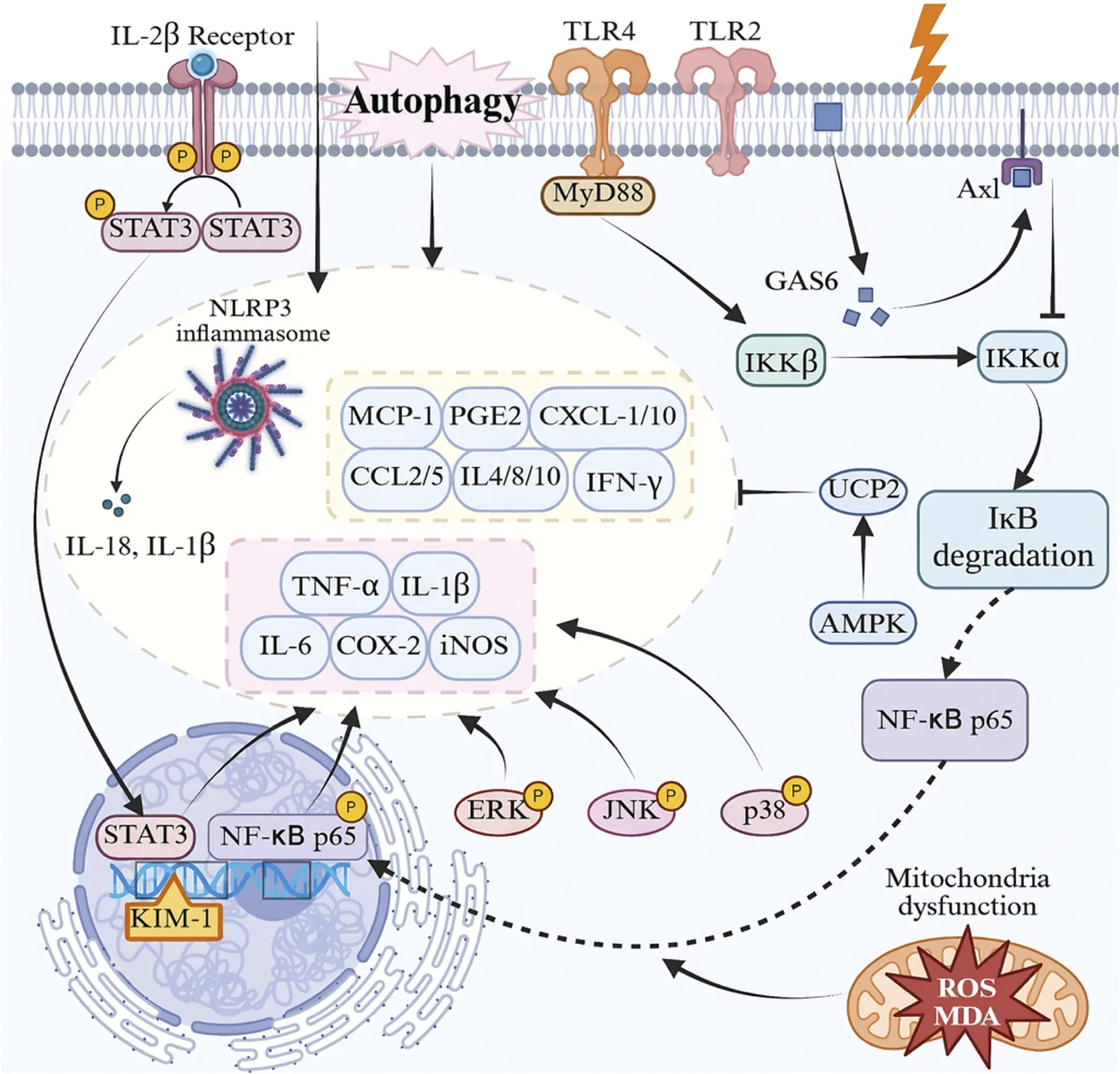
Inflammatory Cascade. This figure summarizes key inflammatory events triggered by ischemia/reperfusion injury, including DAMP-mediated activation of TLRs, upregulation of chemokines (e.g., MCP-1, CXCL1) and adhesion molecules (e.g., ICAM-1), recruitment of immune cells, pro-inflammatory cytokine release (TNF-α, IL-1β, IL-6, IL-17, IFN-γ), activation of the NLRP3 inflammasome, and enhanced iNOS/COX-2 activity leading to NO and PGE_2_ production. MMP-2/9 disrupt extracellular matrix integrity, and NF-κB/MAPKs (p38, JNK) coordinate these pathways, promoting tubular apoptosis and necrosis. Natural metabolites may modulate these pathways at multiple intervention points. Solid arrows indicate activation, dashed arrows indirect regulation, T-bar/flat-head arrows inhibition, upward/downward arrows increased/decreased activity, and starburst symbols pathway activation.

Beyond oxidative stress, inflammation constitutes a central amplifying mechanism in ischemic AKI (Figure 2). The cellular damage that occurs during ischemic episodes followed by reperfusion is associated with the release of damage-associated molecular patterns (DAMPs). These DAMPs subsequently activate pattern recognition receptors, particularly Toll-like receptors (TLRs), thereby triggering innate immune responses within the renal microenvironment (Malathi et al., 2025).

Activation of TLRs rapidly upregulates expression of chemokines, such as monocyte chemoattractant protein-1 and chemokine (C-X-C motif) ligand 1, and adhesion molecules like intercellular adhesion molecule-1 (ICAM-1) (Cantoni et al., 2020). Figure 2 illustrates how these molecules facilitate the recruitment of neutrophils and monocytes/macrophages to sites of renal injury. Infiltrating immune cells, notably pro-inflammatory M1 macrophages, along with stressed tubular epithelial cells, secrete cytokines including tumor necrosis factor-alpha (TNF-α), interleukin-1 beta (IL-1β), interleukin-6 (IL-6), interleukin-17, and interferon-gamma (IFN-γ), creating a self-perpetuating inflammatory environment (Song et al., 2024; Wang Y. et al., 2025).

A central mechanism within this cascade is the activation of the NLRP3 inflammasome, leading to caspase-1-dependent proteolytic maturation of IL-1β and IL-18, which exacerbates inflammatory tissue damage (Han S. J. et al., 2020). Concurrently, the upregulation of inflammatory enzymes, specifically iNOS and cyclooxygenase-2 (COX-2), leads to an augmented synthesis of NO and prostaglandin E_2_ (Farahani et al., 2025). Furthermore, increased activity of matrix metalloproteinases (MMPs), particularly MMP-2 and MMP-9, facilitates extracellular matrix breakdown, thereby undermining the integrity of microvasculature (Tan and Liu, 2024).

These inflammatory responses are orchestrated by upstream signaling pathways. NF-κB and mitogen-activated protein kinases (MAPKs), including p38 and c-Jun N-terminal kinase (JNK), serve as central regulators of this inflammatory network (Yue et al., 2022). Their persistent activation establishes a positive feedback loop with oxidative stress pathways, ultimately causing tubular apoptosis, necrosis, and functional decline (Reid and Scholey, 2021). As highlighted in Figure 2, plant metabolites may mitigate ischemic AKI by modulating NF-κB/MAPK signaling, suppressing inflammasome activation, and reducing pro-inflammatory enzyme activity. Consequently, inflammatory cascades represent critical therapeutic targets for multitarget phytochemicals in ischemic AKI.

### 3.3 Endoplasmic reticulum stress (ERS) and mitochondrial dysfunction

**FIGURE 3:**
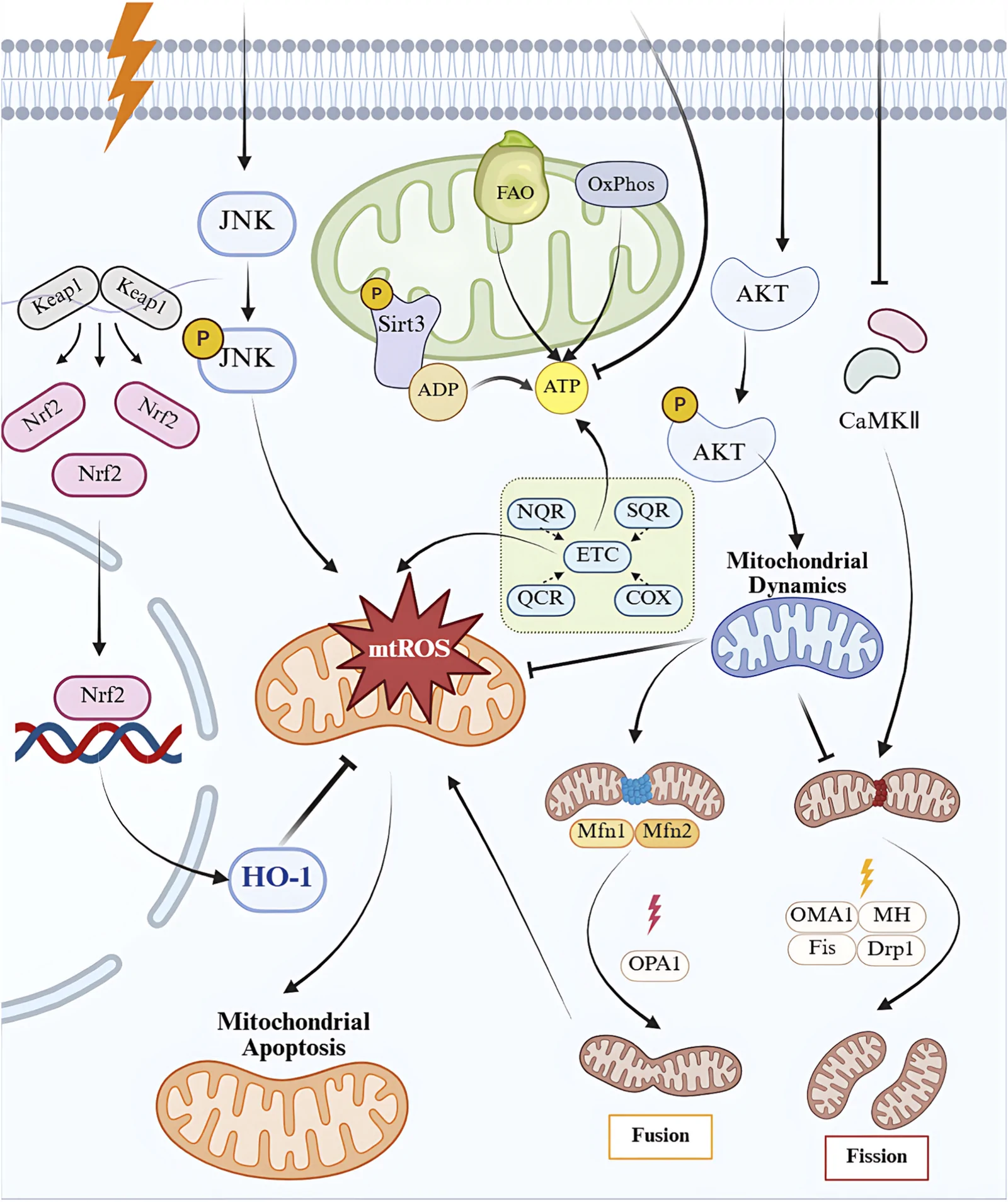
Mitochondrial dysfunction-associated mechanisms. This figure summarizes mitochondrial oxidative stress, impaired oxidative phosphorylation and fatty acid oxidation, reduced ATP production, disrupted mitochondrial dynamics, and mitochondria-related apoptosis in ischemic AKI. mtROS accumulation aggravates mitochondrial injury, whereas the Nrf2/HO-1 axis and stress-related pathways, including JNK, AKT, and CaMKII, regulate mitochondrial homeostasis. Mfn1, Mfn2, and OPA1 are mainly associated with mitochondrial fusion, whereas OMA1, Fis1, and Drp1 are associated with mitochondrial fission. Natural metabolites may protect against mitochondrial injury by reducing mtROS, restoring energy metabolism, and improving fission/fusion balance. Solid arrows indicate activation, dashed arrows indirect regulation, T-bar/flat-head arrows inhibition, upward/downward arrows increased/decreased activity, and starburst symbols pathway activation.

ERS and mitochondrial dysfunction are critical pathological mechanisms underlying ischemic AKI (Figure 3). The ischemic-reperfusion process disrupts the normal protein folding mechanisms within the endoplasmic reticulum, thereby activating the unfolded protein response (Chen et al., 2024). Prolonged stress conditions may trigger ERS-induced apoptosis, characterized by elevated expression of key markers such as glucose-regulated protein 78 (GRP78), C/EBP-homologous protein (CHOP), caspase-12, and activating transcription factor 4 (ATF4) (Zhao et al., 2020; Lu et al., 2025). Furthermore, the upregulation of microRNA-1271 has been shown to inhibit CHOP, a process that is modulated by various signaling pathways, including Sestrin1/2, JNK, PI3K/AKT, JAK/STAT, and Nrf2/HO-1 (Mahtal et al., 2022). These findings suggest that ERS is not an isolated event but is mechanistically connected with oxidative stress, mitochondrial signaling, and apoptosis-related pathways. However, most evidence remains derived from preclinical models, and direct validation of ER–mitochondria crosstalk in human ischemic AKI is still limited.

Mitochondria are central hubs of cellular energy metabolism and are highly susceptible to ischemic AKI-induced injury, displaying altered morphology (increased fission), diminished membrane potential, reduced ATP production, and elevated ROS generation (Xu X. et al., 2025). Mitochondrial injury is characterized by impaired oxidative phosphorylation, reduced fatty acid oxidation, decreased ATP generation, and increased production of mitochondrial reactive oxygen species (mtROS). A study reported a reduction in mitochondrial membrane potential and diminished activities of electron transport chain enzymes, including NADH-quinone reductase, succinate-quinone reductase, ubiquinol-cytochrome c reductase, and cytochrome c oxidase, accompanied by reduced ATP levels and a lower mitochondrial copy number in ischemic AKI. These changes were partially reversed by modulation of peroxisome proliferator-activated receptor-γ coactivator-1α (PGC-1α) and sirtuin 3 (Sirt3) expression, accompanied by increased levels of mitochondrial fission proteins, including mitochondrial fission factor, fission 1, dynamin 1, and dynamin-related protein 1 (DRP1); decreased expression of fusion proteins (mitofusin 1/2); and upregulated expression of mitochondrial autophagy-related genes, including parkin RBR E3 ubiquitin protein ligase and optineurin (OPTN) (Pan and Zhu, 2026). These observations support the central role of mitochondrial quality control in ischemic AKI; nevertheless, the causal relationship between individual fission/fusion markers and renal functional recovery requires cautious interpretation, especially when based only on marker expression rather than functional mitochondrial assays.

Selective modulation of mitochondrial fission markers OMA1 zinc metallopeptidase (OMA1), optic atrophy 1 (OPA1), and PTEN-induced kinase 1 (PINK1) precludes mitochondrial fragmentation by suppressing calcium/calmodulin-dependent protein kinase II (CAMKII) activation. Mitochondrial injury is further modulated by autophagy-related 7 (Atg7) and hypoxia-inducible factor-1α (HIF-1α), along with PGC-1α–dependent mechanisms. Dysregulated expression of mitochondrial DNA copy number, CYP2E1, GPX1, sulfiredoxin 1, PGC-1α, PGC-1β, translocase of outer mitochondrial membrane 20, and mitochondrial transcription factor A (TFAM) in ischemic AKI can be modulated by sirt1, thereby enhancing mitochondrial homeostasis (Zhao et al., 2023). These pathways converge on mitochondrial dynamics, including the balance between fusion-related proteins such as Mfn1/Mfn2 and OPA1 and fission-related proteins such as OMA1, Fis1, and DRP1. Natural metabolites may act at these target sites by limiting mtROS accumulation, preserving mitochondrial membrane potential, and improving mitochondrial quality control.

The downregulation of oxidative phosphorylation-related genes, specifically Atp5a1, COX5b, and SDHb, alongside fatty acid oxidation-related genes such as ACSL1 and ACADM, leads to a decrease in ATP production (Huang et al., 2024). Prolonged downregulation may result in the collapse of the mitochondrial membrane potential. Additionally, reductions in TFAM, ND3, and the mitochondrial DNA copy number further compromise mitochondrial biogenesis. Through network pharmacology approaches, PPARα, STAT3, and HIF-1α have been identified as potential transcription factors that regulate mitochondrial quality. Notably, enhanced PPARα expression modulates BNIP3, which in turn improves mitochondrial morphology and function (Yao et al., 2022). Furthermore, the activation of Nrf2 signaling is associated with improved mitochondrial function, while the upregulation of GPX4 mitigates mitochondrial damage and inhibits ERS (Esteras and Abramov, 2022). The enhancement of mitochondrial function is also linked to the downstream factor UCP2, although the exact nature of this relationship is still under investigation. Overall, the evidence indicates that ERS and mitochondrial dysfunction are interconnected therapeutic targets in ischemic AKI. However, the strength of evidence varies across study types: *in vitro* studies provide mechanistic clues, animal models offer functional and histological support, whereas clinical evidence remains insufficient. Future studies should prioritize dose–response validation, appropriate positive and negative controls, pharmacokinetic characterization, taxonomic authentication of botanical drugs, and confirmation that mitochondrial protection translates into clinically relevant renal outcomes.

### 3.4 Cell death

#### 3.4.1 Ferroptosis

**FIGURE 4:**
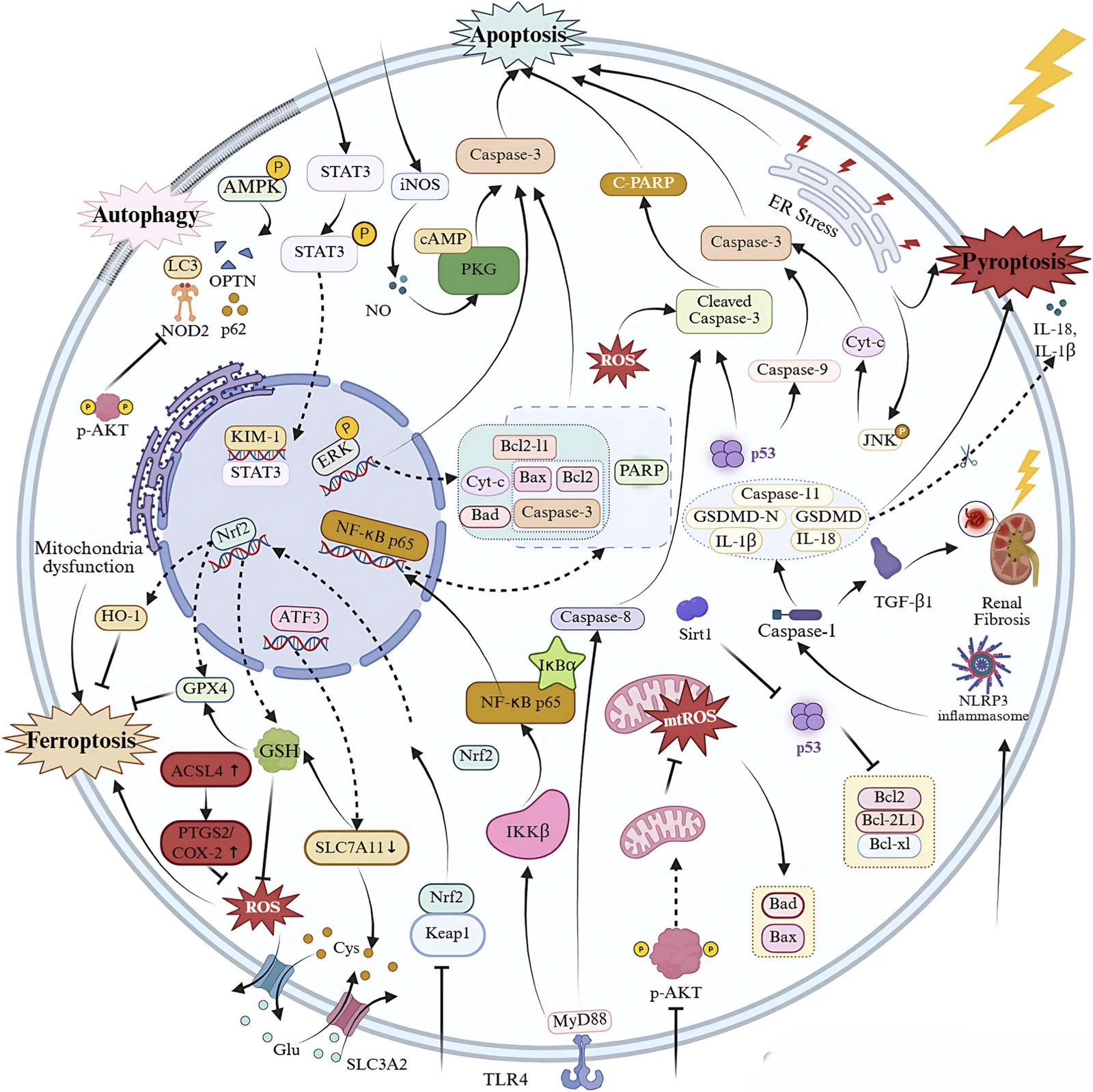
Regulated cell death pathways. This figure summarizes the major forms of regulated cell death, including ferroptosis, apoptosis, and pyroptosis. Ferroptosis involves iron accumulation, lipid peroxidation, decreased GSH, GPX4 and SLC7A11 suppression, and upregulation of ACSL4 and PTGS2/COX-2. Apoptosis occurs via mitochondrial and death receptor pathways, regulated by Bcl-2 family proteins, caspases, cytochrome c, and JNK/ERK/PI3K/AKT/p53 signaling. Pyroptosis is mediated by NLRP3 inflammasome, caspase-1/4, GSDMD cleavage, and IL-1β/IL-18 release. Natural metabolites may modulate these pathways at multiple intervention points. Solid arrows indicate activation, dashed arrows indirect regulation, T-bar/flat-head arrows inhibition, upward/downward arrows increased/decreased activity, and starburst symbols pathway activation.

Ferroptosis represents an iron-dependent mechanism of regulated cell death, predominantly initiated by lipid peroxidation (Figure 4). This process is marked by a significant reduction in GSH levels and an increase in ROS. The modulation of ferroptosis can be achieved through the targeting of GPX4; specifically, the inhibition of GPX4 leads to a decrease in intracellular glutathione levels and intensifies lipid peroxidation (Chu et al., 2023). Additionally, the cystine/glutamate antiporter (System Xc^−^) plays a crucial role in regulating ferroptosis by facilitating cystine uptake, with its inhibition resulting in accelerated ferroptotic cell death (Tu et al., 2021). Important molecular markers associated with ferroptosis include Fe^2+^, GPX4, ACSL4, SLC7A11, SLC3A2, ATF3, and TFR1. Notably, ACSL4 and PTGS2/COX-2 are upregulated, while SLC7A11 is downregulated during ferroptosis, reflecting canonical ferroptotic marker changes. PTGS2 should be considered a transcriptional/enzymatic marker of ferroptosis rather than a protective factor (Tang Q. et al., 2023).

A defining characteristic of ferroptosis is the pronounced increase in lipid peroxidation and oxidative stress (Sun et al., 2025). The accumulation of intracellular iron facilitates Fenton reactions, which produce highly reactive hydroxyl radicals that target unsaturated fatty acids within cellular membranes, thereby initiating a cascade of lipid peroxidation (Lotfipour Nasudivar et al., 2024). Consequently, excessive iron levels and compromised antioxidant defenses emerge as significant contributors to the process of ferroptosis. In the context of ischemic AKI, ferroptosis plays a critical role in the death of renal tubular epithelial cells, exacerbating inflammation and tissue damage (Huang L. et al., 2025). However, most studies are based on *in vitro* and animal models, and robust validation in human samples is limited, highlighting the need for caution when extrapolating mechanistic conclusions.

Ferroptosis is also intricately connected to autophagy. In ischemic AKI, dysregulation of autophagy can lead to the accumulation of harmful intracellular substances, thereby facilitating ferroptosis. Notably, autophagy-related markers such as OPTN, P62, LC3, and NOD2 exhibit upregulation following injury, indicating that autophagy may serve a dual role in both the clearance of damaged cellular components and the promotion of ferroptotic cell death (Pan et al., 2024).

Natural metabolites, such as polyphenols, flavonoids, and terpenes, mitigate ferroptosis-related damage by decreasing ROS generation and lipid peroxidation, while also enhancing Nrf2-mediated antioxidant defenses and indirectly regulating iron metabolism, including reducing intracellular iron accumulation and modulating NCOA4-associated ferritinophagy (Tong et al., 2026; Tian et al., 2025; Li J. Y. et al., 2024).

#### 3.4.2 Apoptosis

Apoptosis constitutes a predominant mechanism of renal tubular epithelial cell death in ischemic AKI. Both oxidative stress and inflammatory responses trigger apoptosis through intrinsic (mitochondrial) and extrinsic (death receptor) pathways (Li C. et al., 2024).

In ischemic AKI, the dysregulation of Bcl-2 family proteins—specifically the pro-apoptotic Bax and the anti-apoptotic Bcl-2—and the activation of caspases are critical to the apoptotic process (Havasi and Borkan, 2011; Thomas et al., 2022). An increase in Bcl-2, Bcl-xl, and Mcl-1, coupled with decreased Bax expression and reduced activity of caspases-3, -8, and -9, contributes to the inhibition of apoptosis in tubular cells. The activation of iNOS leads to the production of NO, which activates downstream protein kinase G signaling, modulating caspase-3 activity (Farahani et al., 2025). In ischemic AKI, the dysregulation of mitochondrial proteins such as mitochondrial cytochrome c, cytoplasmic cytochrome c, BCL2L1, BAD, and Smac/DIABLO is evident. Pharmacological strategies can partially alleviate mitochondria-mediated apoptosis via the JNK, ERK, and PI3K/AKT signaling pathways. Additionally, inhibiting p53-mediated ATP depletion and targeting STAT3 may reduce apoptosis. The activation of Sirt1 and sphingosine-1-phosphate kinase 1 (SPHK1) modulates p53 and PUMA, further suppressing mitochondria-induced apoptosis. Necroptosis exacerbates inflammatory responses, with the inhibition of receptor-interacting protein kinases 1 and 3 (RIPK1 and RIPK3) and mixed lineage kinase domain-like protein (MLKL) significantly diminishing necroptosis and protecting renal tissue (Yang et al., 2023). Emerging evidence suggests that this protective effect may be mediated through the suppression of the IGFBP7/IGF1R axis (Yang et al., 2021).

Dysregulation of Bcl-2 family proteins (Bax, Bcl-2, Bcl-xl, Mcl-1) and activation of caspases are critical. Figure 4 highlights these pathways and Natural Product intervention points. Mitochondrial proteins such as cytochrome c, BCL2L1, BAD, and Smac/DIABLO mediate apoptosis, while signaling via JNK, ERK, PI3K/AKT, p53, STAT3, Sirt1, and SPHK1 modulates these processes. Necroptosis involving RIPK1/3 and MLKL can exacerbate injury; pharmacological inhibition attenuates tissue damage.

#### 3.4.3 Pyroptosis

Pyroptosis is mediated by inflammasome activation, particularly NLRP3, leading to caspase-1/4 activation, GSDMD cleavage, and IL-1β/IL-18 release (Lu et al., 2020). Figure 4 illustrates this pathway and the potential Natural Product targets. Evidence for direct causality is stronger in knockout or inhibitor studies; many expression-based observations remain correlative, underscoring the importance of distinguishing association from mechanistic proof (Liu X et al., 2025).

### 3.5 Others

During ischemic AKI, various mechanisms, including autophagy dysregulation, microcirculatory dysfunction, endothelial damage, and epigenetic alterations, interact to exacerbate renal injury (Yan et al., 2025). Autophagy dysregulation significantly contributes to renal damage, as both excessive and insufficient autophagy can lead to cell death. Natural metabolites such as costunolide and vitexin have been shown to restore autophagy homeostasis, thereby alleviating damage through the reduction of oxidative stress and enhancement of cellular repair processes (Guler et al., 2023; Chen J. N. et al., 2025).

Microcirculatory dysfunction and endothelial injury impede renal blood flow, with endothelial disruption further intensifying inflammation and edema (Tang et al., 2026). Natural metabolites like salvianolic acid A have demonstrated efficacy in improving microcirculation by enhancing endothelial function, decreasing vascular permeability, and mitigating inflammation in preclinical models (Zhang et al., 2018). Given that dysregulation of autophagic flux mediated by lysosomal membrane proteins is closely associated with kidney disease, targeting this pathway may represent a promising strategy for natural product-based renoprotective interventions.

## 4 Classification and potential advantages of plant metabolites in ischemic AKI

Building on these findings, plant metabolites investigated for ischemic AKI are organized into eight principal categories according to their chemical features, source characteristics, and preparation forms: phenylpropanoid, polyphenols, saponins, alkaloids, terpenoids, traditional Chinese medicine (TCM) formulations, botanical extracts, and others. Polyphenolic metabolites are further divided into non-flavonoid polyphenols and flavonoid metabolites to better reflect their structural diversity. The following sections systematically summarize the renoprotective effects, key molecular mechanisms, therapeutic potential, and translational implications of each category (Supplementary Table 1).

### 4.1 Phenylpropanoids

Phenylpropanoids, including phenolic acids, volatile phenylpropanoid derivatives, and related glycosides, have shown renoprotective potential in ischemic AKI. Their reported effects mainly involve mitochondrial protection, antioxidant defense, inflammation suppression, ER stress alleviation, apoptosis inhibition, ferroptosis regulation, and metabolic remodeling. Among them, schisandrin B and schisantherin A are derived from phenylpropanoid units and can be included within the broader phenylpropanoid family. However, the strength of mechanistic evidence varies considerably among different metabolites. In many studies, pathway involvement is mainly inferred from altered expression or phosphorylation of signaling proteins, whereas direct causal validation is available only in selected cases.

Schisandrin B is a well-studied lignan-type metabolite biosynthetically derived from phenylpropanoid units. In a mouse model of bilateral renal I/R, schisandrin B was administered by oral gavage at 20 or 40 mg/kg/day for 7 days before 45 min of ischemia and 24 h of reperfusion (Xu et al., 2024). This pretreatment ameliorated renal dysfunction, tubular injury, oxidative stress, inflammation, and apoptosis. Transcriptomic analysis, network pharmacology, molecular docking, and target validation identified AKT1 and PI3K/AKT-related signaling as major candidate targets. A subsequent study further showed that schisandrin B at 20 μM protected HK-2 cells against H/R injury and reduced mitochondrial ROS, mitochondrial fragmentation, and apoptosis, partly in association with AKT1 phosphorylation and mitochondrial localization (Xu C. et al., 2025). Compared with studies relying only on expression markers, these studies provide more integrated evidence, including transcriptomics, molecular docking, CETSA/SPR-based target-binding assays, and mitochondrial functional assessments. Nevertheless, the evidence still mainly reflects preventive treatment before ischemic injury. Whether schisandrin B remains effective after reperfusion or during established AKI is unclear.

Cells were pretreated with schisantherin A at concentrations of 5, 10, or 20 μM before H/R injury, which improved cell viability and mitigated oxidative stress, inflammatory cytokine production, and apoptosis (Gong and Wang, 2018). The involvement of PI3K/Akt signaling was supported by increased PI3K and Akt phosphorylation, and the PI3K inhibitor LY294002 partly reversed its protective effects. Therefore, PI3K/Akt is more than a simple expression-associated pathway in this study. However, the evidence remains limited to a single *in vitro* model, without animal validation, pharmacokinetic data, or long-term outcome assessment. Thus, its translational relevance remains preliminary.

Methyl eugenol provides relatively strong evidence for antioxidant pathway regulation. In C57BL/6J mice, methyl eugenol was administered before renal I/R injury, and the dose of 20 mg/kg was selected for further *in vivo* experiments. In HK-2 cells, a concentration of 40 μM was used for H/R experiments (Kuang et al., 2023). Methyl eugenol alleviated renal dysfunction, tubular injury, mitochondrial damage, oxidative stress, and apoptosis. Mechanistically, the study suggested that methyl eugenol promoted Nrf2 nuclear retention through AMPK/GSK3β signaling and increased downstream antioxidant proteins, including HO-1, NQO1, and SOD. Importantly, the application of the Nrf2 inhibitor ML385, Nrf2 siRNA, and AMPK/GSK3β pathway inhibitors strengthened the causal link between this pathway and the protective phenotype. The study also examined renal fibrosis after nonfatal ischemic injury, which improves its translational relevance.

Sesamol also provides comparatively strong mechanistic evidence because Nrf2-deficient mice were used. In a unilateral ischemia model involving right nephrectomy and 30 min ischemia of the left kidney followed by 24 h reperfusion, sesamol was administered at 30 mg/kg/day before the IRI procedure (Xue et al., 2024). Sesamol improved renal function, reduced tubular injury, oxidative stress, and inflammatory mediators, and apoptosis, and increased Nrf2, HO-1, and NQO1 expression. Notably, these protective effects were absent in Nrf2-deficient mice, supporting a causal role of Nrf2 rather than a simple association based on protein expression. However, the study focused only on early injury at 24 h after reperfusion, and whether sesamol improves long-term renal repair, fibrosis, or AKI-to-CKD transition remains unknown.

Salidroside has been investigated in more than one study and appears to regulate both inflammatory-apoptotic injury and ferroptosis-related damage. In HK-2 cells, salidroside at 6.25–25 μM reduced hypoxia/reoxygenation-induced oxidative stress, inflammatory cytokine production, and apoptosis (Sun et al., 2018). The involvement of TLR4/NF-κB signaling was supported by TLR4 overexpression, which weakened the protective effects of salidroside. A later *in vivo* study showed that oral Salidroside pretreatment at 1, 10, or 100 mg/kg/day for 7 days attenuated renal I/R injury in rats, with stronger effects at 10 and 100 mg/kg (Tang et al., 2023b). This study further linked salidroside to PI3K/AKT-associated ferroptosis suppression, including reduced lipid peroxidation and altered ferroptosis-related markers. However, the ferroptosis mechanism still relies largely on pathway and marker changes, and stronger validation using GPX4/SLC7A11 loss- or gain-of-function, ferroptosis rescue experiments, or pathway-specific inhibition would be needed.

Salvianolic acid C is noteworthy because it extends phenylpropanoid research beyond classical antioxidant and anti-inflammatory mechanisms. In male C57BL/6J mice, salvianolic acid C was administered intraperitoneally at 10 mg/kg before I/R injury or cisplatin-induced AKI (Chen et al., 2026). The study showed that salvianolic acid C improved renal function and tubular injury while restoring renal gluconeogenesis through the FOXO1/PGC1α/FBP1 axis. The causal role of this metabolic pathway was supported by an FBP1 inhibitor and FBP1 siRNA experiments, which reversed or weakened the protective effects. This provides stronger mechanistic support than studies relying only on expression analyses. However, the study used only male mice and short-term AKI models. Whether this metabolic regulation applies to females, aged animals, or comorbid models remains unknown.

Other phenylpropanoids, including ligustilide, trans-cinnamaldehyde, anethole, nodakenin, ferulic acid, senkyunolide I, and eugenol, have also shown protective effects in preclinical ischemic AKI models, but their mechanistic evidence is generally less complete. Ligustilide was associated with improved mitochondrial morphology, ATP production, and reduced oxidative stress and apoptosis, possibly involving Sirt3-dependent mitochondrial regulation (Xia et al., 2024). Trans-cinnamaldehyde attenuated renal injury mainly in association with suppression of JNK/p38 MAPK-mediated inflammatory signaling (Chen et al., 2023). Anethole reduced oxidative stress, apoptosis, and inflammatory cytokine release, with changes linked to the HMGB1/TLR2/4–MyD88–NF-κB cascade (Mohamed et al., 2022b). Nodakenin was associated with suppression of ROS-induced NLRP3 inflammasome activation and NF-κB p65 signaling (Liao et al., 2021), while ferulic acid was linked to HIF-1α–CD39/CD73 signaling and adenosine production (Zhou Q. et al., 2018). Senkyunolide I alleviated ER stress and apoptosis, with changes in Nrf2/HO-1/NQO1 and GRP78/CHOP-related markers (Zhu et al., 2022). Eugenol attenuated oxidative and ER stress and was associated with Sestrin2 upregulation (Liu J. et al., 2025). These studies collectively support the multitarget potential of phenylpropanoids, but most conclusions remain associative because they are based primarily on changes in pathway protein expression rather than knockout models, knockdown experiments, rescue assays, or direct target-binding validation.

### 4.2 Polyphenols

Polyphenols are a large and chemically diverse class of metabolites derived from botanical drugs (Tang et al., 2020). To provide clarity and reflect chemical hierarchy, we subdivided this class into non-flavonoid metabolites and flavonoid metabolites (Flavonoids). Flavonoids are a subclass of polyphenols, while non-flavonoids include phenolic acids, stilbenes, and other phenolic derivatives. Mechanistic discussion throughout this section differentiates between pathways that are merely associated with protective effects and those supported by causal evidence, such as knockout, knockdown, siRNA, overexpression, inhibitor, or rescue experiments.

#### 4.2.1 Polyphenols (non-flavonoids)

Curcumin pretreatment at 100 mg/kg before ischemia reduced renal dysfunction, tubular injury, and apoptosis; in tubular epithelial cell H/R models, curcumin also attenuated cell death and mitochondrial injury (Fan et al., 2017). Mechanistically, the protective effect was supported by APPL1 knockdown experiments, which aggravated Akt activation and apoptosis, while Akt inhibition partially reversed the effects of APPL1 knockdown. This provides stronger causal evidence for the APPL1/Akt-related pathway than expression analysis alone. Other studies using rat renal I/R models showed that curcumin at 15, 30, or 60 mg/kg improved renal function and reduced oxidative stress, inflammation, and caspase-3-related apoptosis (Liu et al., 2016; Cui et al., 2021). However, these studies mainly relied on biochemical and protein markers. More recent work also explored curcumin-based delivery systems, including curcumin-loaded nanoparticles, to improve renal targeting and bioavailability (Wang N. et al., 2025). These delivery strategies are translationally relevant, but their long-term safety, tissue distribution, and clinical feasibility remain to be determined.

Resveratrol reduced renal dysfunction, oxidative stress, inflammation, and apoptosis, with protection associated with Nrf2 activation and TLR4/NF-κB suppression (Li et al., 2018). Importantly, Nrf2 siRNA weakened the protective effect *in vitro*, supporting a functional role for Nrf2 rather than a purely correlative relationship. Other studies linked resveratrol to JAK/STAT inhibition, SIRT1 activation, and NLRP3-related inflammatory regulation (Erkasap et al., 2017; Baltaci et al., 2019; Wang et al., 2020; Zhou et al., 2022; Alaasam et al., 2024; Martinez-Rojas et al., 2024). Nevertheless, not all of these pathways were tested with loss- or gain-of-function approaches. Moreover, one recent study reported that resveratrol aggravated H_2_O_2_-induced injury under certain conditions, indicating that its effect may be context-, dose-, and model-dependent (Luo et al., 2025). Therefore, the therapeutic window and cell-context specificity of resveratrol require careful clarification before clinical translation.

Salvianolic acid B, a polyphenolic metabolite derived from the botanical drug *Salvia miltiorrhiza* Bunge, has been studied in renal I/R-related oxidative stress and pyroptosis. In mouse renal I/R models, salvianolic acid B was administered intragastrically at 50, 100, or 200 mg/kg before injury and reduced renal dysfunction, oxidative stress, inflammation, and pyroptosis-related changes (Pang et al., 2020). Its protective effect was associated with Nrf2 nuclear accumulation and suppression of NLRP3 inflammasome activation. The use of the NLRP3 inhibitor MCC950 and the caspase-1 inhibitor VX-765 strengthened the interpretation that inflammasome-related pyroptosis contributes to the observed injury process. However, direct genetic validation of Nrf2 or NLRP3 was not performed in this study, so the Nrf2/NLRP3 axis should be described as a supported pathway rather than a fully established direct target of salvianolic acid B. Additional work also suggests antioxidant and anti-inflammatory activity of salvianolic acid B, but pharmacokinetic exposure, optimal dosing, and post-injury efficacy remain insufficiently defined.

Theaflavin provides another example in which pathway validation was partly strengthened by siRNA experiments. In male C57BL/6J mice and TCMK-1 cells, theaflavin pretreatment alleviated renal dysfunction, oxidative stress, and apoptosis. Its protective effect was associated with Nrf2 activation and suppression of p53-mediated apoptosis (Li et al., 2021). Transfection with p53 siRNA partially inhibited the effect of theaflavin, supporting the involvement of p53-related apoptotic signaling (Zhu et al., 2024).

Salvianolic acid A was reported to improve renal microcirculation and reduce ischemic injury, with effects associated with VEGFA and Klotho upregulation (Zhang et al., 2018). This suggests a potential endothelial-protective role, which is important because microvascular dysfunction contributes substantially to ischemic AKI. However, the evidence remains largely based on expression changes and functional readouts; direct validation using endothelial-specific models or VEGFA/Klotho loss-of-function approaches is still lacking.

Several other polyphenolic metabolites have shown renoprotective effects, but with less extensive mechanistic validation. Pterostilbene reduced renal I/R injury and inflammatory responses, with protection associated with TLR4/NF-κB inhibition (Gao et al., 2018). Ellagic acid attenuated oxidative stress, inflammation, and apoptosis, with effects associated with the NOX4/JAK/STAT pathway (Liu et al., 2020). Oleuropein reduced renal oxidative stress and apoptosis and was linked to AMPK/eNOS signaling (Nasrallah et al., 2020). Gallic acid, chlorogenic acid, tannic acid, rosmarinic acid, and urolithin A generally improved renal functional, oxidative, inflammatory, apoptotic, or mitochondrial indices in experimental renal I/R models (Ahmadvand et al., 2019; Toprak et al., 2020; Alechinsky et al., 2020; Li et al., 2016; Firouzeh et al., 2025; Zhang et al., 2022). However, most of these studies relied primarily on short-term rodent models and pathway marker changes.

#### 4.2.2 Polyphenols (flavonoids)

Flavonoids are widely studied for ischemic AKI due to their abundance in medicinal plants and diet, as well as their ability to target multiple overlapping pathological processes. However, the strength of evidence differs substantially among individual metabolites. In many studies, pathways such as Nrf2/HO-1, NF-κB, MAPK, PI3K/Akt, JAK/STAT, TLR4, NLRP3, GPX4, and SLC7A11 are mainly inferred from altered protein expression or phosphorylation.

Scutellarin provides relatively strong mechanistic evidence for Nrf2-dependent antioxidant protection. The metabolite reduced renal dysfunction, KIM-1 expression, oxidative stress, and histological injury. Molecular docking suggested a possible interaction with Nrf2, and Nrf2 siRNA in HK-2 cells abolished the ability of scutellarin to reduce ROS and upregulate HO-1, supporting a functional role of Nrf2 rather than a simple expression association (Dai et al., 2022). A later study further showed that scutellarin alleviated renal I/R injury by reducing macrophage infiltration and pro-inflammatory macrophage polarization, with protection associated with inhibition of MAPK signaling (Deng et al., 2024). However, macrophage-specific depletion or lineage-targeted gain- and loss-of-function experiments were not performed, so the immune-regulatory mechanism remains partly associative.

Tiliroside is one of the stronger examples of ferroptosis-related pathway validation. Tiliroside was administered by gavage at 30 mg/kg/day before injury; in HK-2 cells, 10 μM Tiliroside was used for mechanistic experiments. Mechanistic studies suggested that tiliroside may disrupt the Keap1-Nrf2 protein-protein interaction, activate the Nrf2/GPX4 pathway, and inhibit ferroptosis (Cai et al., 2024). The conclusion was supported by molecular docking, CETSA-based Keap1 target engagement, and CRISPR/Cas9-mediated Nrf2 knockout in HK-2 cells, which weakened the protective effect. These data provide stronger causal evidence than expression analysis alone. Nevertheless, the study still used pretreatment, and post-reperfusion efficacy and pharmacokinetic exposure remain unclear.

Xanthohumol also has relatively strong evidence for Nrf2-associated ferroptosis regulation. The metabolite improved renal function and histological injury and reduced ROS accumulation, lipid peroxidation, and ferroptosis-related changes. The use of erastin as a ferroptosis inducer and Nrf2 siRNA in HK-2 cells strengthened the interpretation that Nrf2/HO-1-related ferroptosis inhibition contributes to the protective phenotype (Tang et al., 2023a). However, *in vivo* genetic validation was not performed, and the clinical relevance of administration shortly before ischemia remains limited.

Luteoloside has recently been studied with multiple mechanistic validation approaches. In renal I/R mice, luteoloside pretreatment showed dose-dependent protection, with 80 mg/kg producing stronger renoprotective effects; in HK-2 H/R experiments, 80 μM was used for mechanistic validation. Luteoloside reduced oxidative stress, mitochondrial dysfunction, and ferroptosis-related damage. Its protective effect was associated with Nrf2 activation, GPX4/SLC7A11 preservation, and reduced lipid peroxidation. Importantly, Nrf2 siRNA in HK-2 cells and the Nrf2 inhibitor ML385 in mice weakened its antioxidant and anti-ferroptotic effects. Additional reporter and ubiquitination-related assays suggested that luteoloside may stabilize Nrf2 through Keap1-related regulation (Qiu et al., 2026). These findings provide stronger mechanistic support, although post-injury treatment and long-term AKI-to-CKD outcomes remain to be evaluated.

Earlier work showed that hydroxysafflor yellow A alleviated renal dysfunction, oxidative stress, apoptosis, and inflammatory injury, with protection associated with suppression of TLR4/NF-κB signaling (Bai et al., 2018). A later HK-2 H/R study used 20 μg/mL hydroxysafflor yellow A and demonstrated that the Akt inhibitor LY294002 attenuated its protective effects, supporting involvement of the Akt/GSK-3β/Fyn-Nrf2 axis (Wang et al., 2022a). Thus, compared with studies relying only on pathway expression, the latter work provides stronger pharmacological evidence. Nevertheless, the *in vivo* evidence remains mainly preventive, and genetic validation of Akt, Fyn, or Nrf2 is still lacking.

Rats received puerarin at 50 or 100 mg/kg once daily for 7 days before renal I/R injury, and HK-2 cells were treated with 1 or 10 μM before H/R injury (Wang J. et al., 2025). Puerarin reduced renal dysfunction, apoptosis, and ER stress, with protection associated with increased Nrf2/HO-1 and decreased caspase-3 and CHOP expression. However, the study did not provide Nrf2 knockdown, inhibitor, or rescue evidence.

Naringin has been studied both alone and in combination with trimetazidine. In rat renal I/R injury, naringin was administered intraperitoneally at 100 mg/kg for 7 days, while trimetazidine was given intravenously before reperfusion in combination studies (Amini et al., 2019). These studies showed improvement in renal and remote cardiac oxidative injury markers, with effects associated with increased Nrf2 expression and antioxidant capacity. A more recent study reported that naringin affected caspase-3, IL-1β, HIF-1α, and TNF-α in renal I/R injury (Danis et al., 2024). However, most mechanistic conclusions are based on marker changes, and the combination studies should not be interpreted as evidence that naringin alone accounts for all observed effects.

Earlier studies showed that luteolin reduced renal dysfunction, oxidative stress, inflammation, and apoptosis in renal I/R models (Hong et al., 2017; Kalbolandi et al., 2019). A recent study used a unilateral renal I/R model to evaluate AKI-to-CKD transition, administering luteolin at 50 mg/kg/day by oral gavage starting after surgery for 30 days (Wei et al., 2025). This post-injury and long-term design is more clinically relevant than pretreatment-only experiments. Luteolin reduced fibrosis, inflammation, apoptosis, oxidative stress, and Nrf2/HO-1-related changes. However, the authors noted that gene knockout or overexpression was not performed, so the Nrf2/HO-1 pathway remains an associated mechanism rather than a confirmed causal target.

Astilbin represents a distinctive strategy because it was used to pretreat mesenchymal stem cells rather than simply administered as a free metabolite. Astilbin-pretreated MSCs improved renal injury and AKI-to-CKD outcomes in mouse I/R models. Mechanistically, astilbin was shown to interact with KLF4, and KLF4 promoted PTGS2 transcription, thereby enhancing the ability of MSCs to promote M2 macrophage polarization. The study used molecular docking, SPR, DARTS/LC-MS/MS, ChIP-PCR, dual-luciferase reporter assays, KLF4 overexpression, and PTGS2 knockdown; notably, PTGS2 knockdown reversed the beneficial effects of Astilbin-pretreated MSCs (Geng et al., 2024). This provides strong causal evidence for the KLF4/PTGS2 axis in this MSC-based strategy. However, this is a cell-therapy enhancement approach rather than direct flavonoid therapy, and its manufacturing standardization, safety, and clinical scalability require further evaluation.

Other flavonoid metabolites, as summarized in Supplementary Table 1, also showed renoprotective effects in preclinical renal I/R or H/R models. These include metabolites mainly associated with antioxidant and anti-inflammatory responses, such as quercetin, hesperidin, genistein, nobiletin, eriocitrin, and chrysin; metabolites linked to mitochondrial protection or apoptosis regulation, such as hyperoside, apigenin, tilianin, fisetin, oroxylin A, and isoquercitrin; and metabolites reported to affect ferroptosis, pyroptosis, autophagy, or macrophage-related responses, such as silibinin, naringenin, loureirin C, proanthocyanidin B2, cyanidin-3-O-glucoside, astragalin, and tangeretin. Overall, these studies support the broad pharmacological potential of flavonoid metabolites, but most rely on short-term animal or tubular cell models and pathway marker changes.

### 4.3 Saponins

Saponins have shown renoprotective potential in ischemic AKI through regulation of pyroptosis, necroptosis, oxidative stress, mitochondrial quality control, inflammation, and macrophage polarization.

Platycodin D provides one of the more mechanistically detailed examples among saponins. In a diabetic renal I/R model, rats were treated with platycodin D by gavage at 15 or 30 mg/kg before I/R injury, and HK-2 cells under high-glucose H/R conditions were also used (Qin et al., 2025). Platycodin D reduced renal injury, oxidative stress, apoptosis, and inflammation, while improving mitochondrial quality control. Mechanistically, transcriptomic analysis suggested enrichment of AMPK and mitophagy-related pathways. Functional experiments further showed that platycodin D increased AMPK phosphorylation and promoted PINK1/Parkin-mediated mitophagy, while also suppressing MAPK/NF-κB-related inflammatory signaling. Importantly, the AMPK inhibitor Compound C *in vivo* and AMPK siRNA in HK-2 cells abolished these effects, supporting a causal role for AMPK rather than a simple association based on expression changes. Nevertheless, the study was conducted in a diabetic I/R context, and whether this mechanism applies to non-diabetic ischemic AKI, female animals, aged models, or long-term AKI-to-CKD transition remains unclear.

Madecassoside, a triterpenoid saponin, provides relatively strong mechanistic evidence compared with many other studies. The study showed that madecassoside reduced renal injury, oxidative stress, inflammation, and apoptosis. Mechanistically, RNA sequencing and molecular docking suggested JNK/c-JUN signaling as a target, and the study further reported that madecassoside may directly bind JNK kinase. Its protective effect was compared with curcumin and the JNK inhibitor SP600125, supporting JNK/c-JUN involvement beyond simple expression analysis (Shan et al., 2024). However, more rigorous validation using JNK knockdown/overexpression or rescue experiments would still be needed to establish JNK as the indispensable causal target in ischemic AKI.

Gypenoside XVII was pretreated with 10, 20, or 40 mg/kg/day before 40 min bilateral renal ischemia, with 4-PBA used as a positive control for ER stress inhibition. Gypenoside XVII reduced renal dysfunction, tubular injury, inflammatory cytokines, ER stress markers, and pyroptosis-related proteins, including NLRP3, caspase-1, GSDMD, and IL-1β (Wang J. et al., 2024). These findings suggest that gypenoside XVII may protect against renal I/R injury by suppressing ER stress-associated NLRP3 inflammasome activation.

Sodium aescinate also targets pyroptosis-related injury. In a mouse renal I/R model, sodium aescinate was administered intraperitoneally at 2.5 or 5 mg/kg before surgery, and HK-2 H/R experiments were used for mechanistic exploration. The study showed reduced renal dysfunction and decreased expression of pyroptosis-related markers, including NLRP3, caspase-1, GSDMD, and IL-1β. The use of the AKT agonist SC79 in HK-2 cells strengthened the mechanistic interpretation by showing that activation of AKT weakened the inhibitory effect of sodium aescinate on inflammatory and pyroptosis-related responses (Liu X. et al., 2025). Nevertheless, the *in vivo* evidence remains based on pretreatment, and more rigorous validation using NLRP3 or GSDMD knockdown/knockout models would be required to establish pyroptosis as the causal therapeutic target.

Several saponins have been linked to immune regulation and macrophage polarization. Asiaticoside reduced renal dysfunction and inflammatory injury in renal I/R models and was associated with a shift toward anti-inflammatory M2 macrophage polarization (Tang et al., 2022). Ginsenoside Rd also attenuated renal I/R injury in mice and reduced M1 macrophage-related inflammatory responses, with *in vivo* data showing protection at 50 mg/kg and *in vitro* experiments using macrophage cultures to assess polarization-related effects (Ren et al., 2016). These studies are valuable because they move beyond tubular epithelial cells and examine the immune microenvironment. However, macrophage polarization was mainly evaluated using phenotypic markers and cytokine profiles. Whether these macrophage changes are required for renal protection remains uncertain, as macrophage depletion, adoptive transfer, or lineage-specific gain- and loss-of-function experiments were not performed.

Other saponins have shown protective effects through oxidative stress, inflammation, necroptosis, or cytokine regulation, but their mechanistic evidence is generally less complete. Astragaloside IV was associated with activation of the Keap1-Nrf2/ARE pathway and improvement of oxidative stress in rat renal I/R injury (Su et al., 2022), while clematichinenoside protected HK-2 cells against H/R injury through Nrf2/HO-1-related antioxidant responses, with Nrf2 knockdown reversing its protective effects (Feng et al., 2020). Gypenoside XLIX was reported to inhibit necroptosis through the IGFBP7/IGF1R pathway (Yang et al., 2021), and Notoginsenoside R1 reduced inflammatory and oxidative markers while balancing pro- and anti-inflammatory cytokines in experimental renal I/R injury (Fan et al., 2020). Earlier work on gypenoside suggested anti-inflammatory and antioxidant effects associated with ERK signaling and reduced MDA/increased SOD levels (Ye et al., 2016). Overall, these findings support a broad protective profile for saponins, but many studies still rely on pathway marker expression, short-term injury endpoints, and pretreatment designs.

### 4.4 Alkaloids

Berberine is among the most extensively studied alkaloids in this field. Berberine reduced apoptosis and mitochondrial dysfunction, with mechanistic evidence linking its protective effects to Sirt1/p53 signaling and SPHK1 downregulation (Lin et al., 2018; Lu et al., 2018). These studies used *in vitro* gain- or loss-of-function approaches, including Sirt1 overexpression or silencing and SPHK1-related validation, which strengthens the causal interpretation compared with simple expression analysis. More recent work extended berberine research to the gut-kidney axis: in rats with renal I/R injury, berberine was administered by gavage at 150 mg/kg/day for 14 days before ischemia, and the study showed improvement in renal injury, intestinal barrier dysfunction, gut microbiota composition, and intestinal TLR4/NF-κB activation (Huo and Wang, 2024). Another recent study suggested that berberine attenuates H/R-induced pyroptosis in HK-2 cells through the FOXO3a/ARC axis (Wang and Huang, 2025). Collectively, these studies indicate that Berberine may protect through multiple interconnected mechanisms, including mitochondrial stress, apoptosis, inflammation, pyroptosis, and gut-derived inflammatory signaling. Nevertheless, most evidence remains preclinical, and the high oral doses used in animals raise questions regarding human-equivalent exposure, bioavailability, active metabolites, and clinical feasibility.

Leonurine was evaluated in a rat renal I/R model in which SD rats received oral leonurine at 7.5, 15, or 30 mg/kg/day for 7 days before 45 min renal ischemia followed by 24 h reperfusion. Leonurine reduced Scr and BUN levels, tubular injury, oxidative stress, and inflammatory cytokine release (Han et al., 2022). The study reported increased Nrf2-related antioxidant proteins and decreased TLR4/NF-κB pathway activation, suggesting that leonurine may exert dual antioxidant and anti-inflammatory effects. However, these mechanistic conclusions are mainly based on expression and phosphorylation analyses. No Nrf2 knockdown/knockout, TLR4 inhibition, or NF-κB rescue experiments were performed. Therefore, Nrf2 and TLR4/NF-κB should be regarded as associated pathways rather than fully demonstrated causal targets.

Anisodamine provides stronger evidence for an anti-apoptotic mechanism. In a rat renal I/R model established by right nephrectomy and 45 min left renal pedicle clamping followed by 24 h reperfusion, anisodamine reduced renal dysfunction, histological injury, and tubular epithelial apoptosis (Zhang et al., 2021). Mechanistically, anisodamine increased ERK phosphorylation and Bcl-2 expression while reducing Bax and cleaved caspase-3. Importantly, the MEK/ERK inhibitor PD98059 partially reversed these protective effects, supporting the involvement of ERK signaling in the anti-apoptotic effect. However, this evidence remains limited to an acute rat model, and the study did not address delayed treatment, long-term renal repair, or pharmacokinetic properties.

Tetramethylpyrazine hydrochloride was administered intraperitoneally at 40 mg/kg after reperfusion and reduced renal dysfunction, inflammation, and tubular injury, with changes associated with HIF-1α and NLRP3 inflammasome-related signaling (Sun et al., 2020). A later study also used 40 mg/kg tetramethylpyrazine after reperfusion and suggested protection through modulation of DKK1/Wnt/β-catenin-related repair responses (Wang X. et al., 2025). These post-reperfusion designs are more clinically relevant than pretreatment-only studies.

Other alkaloids have shown protective activity, but most require stronger mechanistic validation. Evodiamine reduced NF-κB-associated inflammation and caspase-3-related apoptosis in renal I/R injury (Eraslan et al., 2019). Nuciferine improved renal dysfunction after I/R and was associated with reduced oxidative stress, NF-κB inhibition, and restoration of autophagy-related clearance of damaged mitochondria (Sallabi et al., 2025). Oxymatrine and piperlongumine mainly reduced inflammation-associated oxidative damage and increased antioxidant enzyme activity, including SOD and CAT (Ozturk et al., 2017; Xiao and Vijayalakshmi, 2022). Neferine was linked to inhibition of IκB-α degradation, reduced NF-κB activation, and Klotho upregulation, which may indirectly suppress TLR4 signaling (Li H. et al., 2019). Rutaecarpine reduced inflammatory cytokines and adhesion molecules in association with JNK/p38 MAPK inhibition (Wang et al., 2017). Piperine showed dose-dependent vascular protection by reducing iNOS upregulation and preserving eNOS activity, thereby limiting peroxynitrite formation (Mohammadi et al., 2020). Ligustrazine alleviated renal I/R injury by inhibiting NOD2-mediated inflammation, and autophagy appeared to contribute to this effect (Jiang et al., 2020).

### 4.5 Terpenoids

Poricoic acid A is one of the more translationally relevant terpenoids because it was evaluated in the context of AKI-to-CKD transition rather than only early ischemic injury. In a rat renal I/R model, poricoic acid A was administered orally at 10 mg/kg/day after reperfusion, together with melatonin treatment, and was shown to enhance the protective effect of melatonin against renal dysfunction, oxidative stress, inflammation, and fibrotic progression (Chen et al., 2019). Mechanistically, the protective effect was associated with regulation of the Gas6/Axl-NF-κB/Nrf2 axis. This study is valuable because it addresses post-injury treatment and longer-term maladaptive repair, which are closer to clinical concerns than pretreatment-only designs. Nevertheless, the contribution of poricoic acid A alone versus its interaction with melatonin requires clearer separation, and stronger causal validation of the Gas6/Axl-NF-κB/Nrf2 axis using knockdown, pathway blockade, or rescue experiments would further strengthen the conclusion.

Lycopene has recently been investigated using both network-based and target-focused approaches. In a rat renal I/R model, oral lycopene pretreatment at 100 mg/kg/day for 7 days improved renal function and histological injury, with network pharmacology and experimental validation suggesting involvement of inflammation- and oxidative stress-related pathways (Pan et al., 2024). A more recent study further tested lycopene in cisplatin- and I/R-induced AKI models and identified PARP1 as a potential target (Hu et al., 2026). This study is mechanistically stronger because it used PARP1 inhibitor comparison, CETSA-based target engagement, and PARP1 knockdown strategies, supporting a more direct link between PARP1 inhibition and the protective phenotype. Nevertheless, the dosing strategy remains preclinical, and whether sufficient renal tissue exposure can be achieved safely in humans remains unclear.

Crocin has been examined in more than one renal I/R study and therefore represents a relatively well-documented carotenoid derivative. In rat models of bilateral or unilateral renal I/R injury, crocin administration improved renal function and reduced oxidative stress and inflammatory markers, including ICAM-1, TNF-α, and TLR4-related responses (Yarijani et al., 2017; Abou-Hany et al., 2018). These findings suggest antioxidant and anti-inflammatory activity, but the mechanistic evidence remains mostly associative because the studies did not use pathway-specific inhibitors, genetic models, or rescue approaches. Thus, crocin should be described as reducing inflammatory and oxidative injury rather than as definitively acting through a specific causal pathway.

Astaxanthin has also been evaluated in multiple rat renal I/R studies. In one study, oral astaxanthin at 50 mg/kg for 7 days before renal ischemia reduced renal injury and improved oxidative stress-related parameters (Arslan et al., 2021). A later study tested different doses of astaxanthin in renal I/R-modeled rats and focused on oxidative damage and autophagy-related markers (Kisaoglu et al., 2024).

Other terpenoids have shown renoprotective activity but are supported by more limited mechanistic validation. Ursolic acid and (−)-α-bisabolol reduced renal dysfunction, oxidative stress, and tubular injury markers in experimental renal I/R models (Peng et al., 2016; Sampaio et al., 2016). Tanshinone IIA attenuated renal I/R injury and was associated with reduced pro-inflammatory cytokines and p38 MAPK activation (Xu et al., 2016), while perillyl alcohol protected HK-2 cells against H/R injury, with PI3K/Akt/eNOS involvement supported by PI3K inhibitors LY294002 and wortmannin (Xu et al., 2017). Carnosol reduced inflammation and apoptosis in rat renal I/R injury, with changes associated with p38 MAPK and caspase-3 signaling (Zheng et al., 2018). Taraxasterol, geraniol, celastrol, glaucocalyxin A, fucoxanthin, loganin, ganoderic acids, picroside II, myrrh essential oil, lupeol, myrtenal, α-pinene, and β-elemene also improved renal functional, oxidative, inflammatory, apoptotic, or mitochondrial injury indices in preclinical models (Li et al., 2020; Mohamed et al., 2022a; Younis and Ghanim, 2022; Hosohata et al., 2021; Mao et al., 2022; Huang et al., 2022; Shao et al., 2021; Ren et al., 2024; Younis, 2023; Kapisiz et al., 2024; Beytur et al., 2025; Ozbek Sebin et al., 2025). Among these, β-elemene is notable because MyD88 siRNA was used, providing stronger support for the involvement of MyD88-dependent inflammatory signaling than marker expression alone.

### 4.6 Traditional Chinese Medicine (TCM)

Traditional Chinese Medicine (TCM) formulations differ from single metabolites because they contain multiple botanical drugs and numerous chemical metabolites. In ischemic AKI models, these formulas have shown protective effects mainly associated with anti-inflammatory, antioxidant, anti-apoptotic, and anti-fibrotic responses. Their multicomponent nature may theoretically allow coordinated regulation of multiple pathological processes; however, this same complexity also creates major challenges for standardization, quality control, active metabolite identification, pharmacokinetic evaluation, and mechanistic validation (Cao D et al., 2024; Liu et al., 2022).

Shenhua Tablet, containing *Radix Astragali*, *Rhizoma Atractylodis Macrocephalae*, and *Flos Lonicerae*, has been evaluated in a rat renal I/R model. In male Wistar rats, Shenhua Tablet was administered at 1.5 or 3.0 g/kg/day for 1 week before I/R injury, with astragaloside used as a positive control (Li Q. P. et al., 2019). The treatment improved renal function and histological injury at 24 and 72 h after reperfusion and reduced the expression of TLR2, TLR4, MyD88, TNF-α, and IL-6. These findings suggest that it may protect against renal I/R injury through suppression of TLR2/TLR4/MyD88-associated inflammatory signaling. However, the evidence is mainly based on mRNA and protein expression changes. Pathway-specific blockade, immune-cell validation, or active-metabolite attribution was not performed, so the TLR pathway should be considered associated with, rather than definitively proven to mediate, the protective effect.

Joa-Gui Em, composed of *Rehmanniae Radix Preparata*, *Dioscoreae Rhizoma*, and *Corni Fructus*, was studied in a rat acute renal failure model induced by renal I/R injury, with rats orally administered 100 or 200 mg/kg/day for 4 days after I/R surgery (Na et al., 2021). This post-injury treatment design is more clinically relevant than pretreatment-only studies. Joa-Gui Em reduced BUN and SCr levels, improved renal histology, and decreased inflammatory and apoptotic responses. Mechanistically, these effects were associated with downregulation of TXNIP/NLRP3 and TLR4/NF-κB signaling. Nevertheless, the study did not use NLRP3 knockout, TXNIP knockdown, NF-κB inhibition, or rescue experiments. Thus, the proposed mechanism remains largely associative, and the active metabolites responsible for these effects remain unclear.

Xiaoyu Xiezhuo Drink, containing Astragalus membranaceus and Radix cyathulae, was investigated in aged mice with renal I/R injury, which increases the clinical relevance of the model because elderly patients are more susceptible to ischemic AKI. The study combined UHPLC-Q-TOF-MS-based chemical profiling, network pharmacology, bioinformatics analysis, and experimental validation (Ye et al., 2021). Xiaoyu Xiezhuo Drink improved renal dysfunction, inflammatory injury, oxidative stress, and fibrosis-related changes. Network and experimental data suggested involvement of TGF-β1/Smad3 and HIF-1 signaling, along with multiple hub targets such as IL-6, TNF, TP53, VEGFA, PTGS2, TLR4, NOS3, EGFR, PPARG, HIF1A, and HMOX1. This integrative design is useful for exploring multicomponent formulas, but it does not prove the direct causality of individual pathways or metabolites.

Nao-Ling-Su Capsule, a 15-herb formulation, was investigated using network pharmacology and experimental validation in I/R-induced AKI models (Lin et al., 2024). Network analysis identified STAT3, HSP90AA1, and other candidate targets, while molecular docking suggested interactions between major bioactive metabolites and core targets such as STAT3 and KIM-1. *In vivo*, Nao-Ling-Su Capsule pretreatment improved renal function and reduced renal injury; *in vitro*, both the formula and drug-containing serum protected HK-2 cells, with effects associated with inhibition of STAT3 signaling and reduced KIM-1 expression. However, as with many network pharmacology-based studies, docking results only provide predictive evidence. The study would be strengthened by STAT3 knockdown/overexpression, rescue experiments, and identification of the active absorbed metabolites responsible for the observed effects.

### 4.7 Botanical extracts

Total flavonoids from *Rosa laevigata* Michx. fruit provide one of the more mechanistically supported examples. Mechanistically, its effects were associated with activation of Sirt1/Nrf2/HO-1 signaling and suppression of NF-κB-related inflammatory responses. Sirt1 siRNA attenuated the protective effect *in vitro*, supporting a causal role of Sirt1 rather than simple pathway association (Zhao et al., 2016).

Salvianolate, derived from *Salvia miltiorrhiza* Bunge, is another well-documented example. In mouse renal I/R, salvianolate was administered at 10, 30, or 90 mg/kg before ischemia, and renal tubular cell models were used for mechanistic validation (Sun et al., 2022). Protection was associated with activation of Keap1/Nrf2/ARE signaling. Nrf2 siRNA, ARE-luciferase reporter assays, and CETSA-based target engagement provided stronger causal evidence than expression analyses alone.

Huaier extract from *Trametes robiniophila* Murrill was tested in a post-injury design. Oral administration at 6 g/kg/day from day 3 to day 28 after I/R reduced renal fibrosis, tubular apoptosis, and ER stress, with protection associated with miR-1271 upregulation and suppression of GRP78/CHOP-mediated ER stress (Zhao and Wu, 2020). Luciferase reporter experiments supported CHOP as a target of miR-1271. However, the complexity of Huaier extract limits the identification of responsible metabolites and standardization.

D-005 lipid extract from *Acrocomia crispa* (H.B.K.) C.K.E. fruits improved renal function, oxidative stress markers, and histological injury in rat renal I/R models, with protection associated with improved mitochondrial-related energy metabolism (Oyarzabal-Yera et al., 2019). However, this study lacked pathway-specific inhibitors, knockdown, or rescue experiments, so mechanistic conclusions remain associative.

Other botanical extracts targeted apoptosis, mitochondrial function, or specialized pathways. *Stevia rebaudiana* (Bertoni) Bertoni reduced Bax/caspase-3/9 activation and increased Bcl-2 (Elsaid et al., 2019). *Juglans regia* L. and *Agathis robusta* (C.Moore ex F. Muell.) L.A.S.Johnson extracts improved mitochondrial integrity and anti-apoptotic markers (Askin et al., 2022; Mohamed et al., 2022c).

Other botanical extracts have shown consistent renoprotective effects, including reductions in renal dysfunction, tubular injury, oxidative stress, inflammation, apoptosis, and mitochondrial or ER stress, as summarized in Table 1. Mechanistic evidence for these extracts is mostly associative, as detailed in the table, and stronger causal validation is generally lacking.

**TABLE 1:** Protective effect of extracts against ischemic AKI.

Botanical source	Family	Extract	Model	Effect	Mechanism	References
*Nigella sativa* L.	Ranunculaceae Juss	Volatile oil (steam-distilled seed extract, 400 mg/kg, p.o.)	*In vivo*: SD rats (male), BIRI (45 min/24 h)	↓Renal damage, apoptosis, and cell proliferation	↓NF-κB activation	Caskurlu et al. (2016)
*Rydingia persica* (Burm.f.) Scheen & V.A.Albert	Lamiaceae Martinov	Ethanolic extract (aerial parts, 300 mg/kg/day, p.o., 2 weeks)	*In vivo*: Wistar rats (male), UIRI/UNX (45 min/24 h)	↓Renal damage and oxidative stress	NR	Takhtfooladi et al. (2016)
*Rosa laevigata* Michx.	Rosaceae	Total flavonoids (fruit extract, 50, 100, 200 mg/kg, p.o.)	*In vivo*: SD rats (male), BIRI (45 min/24 h); *In vitro*: NRK-52E cells, H/R (6 h)	↓Oxidative stress and inflammation	↑Sirt1/Nrf2/HO-1 and ↓ NF-κB	Zhao et al. (2016)
*Hypericum perforatum* L.	Hypericaceae	Ethanolic extract (50 mg/kg, p.o.)	*In vivo*: SD rats (male), UIRI/UNX (45 min/3 h)	↓Oxidative stress, lipid peroxidation	NR	Cakir et al. (2017)
*Crocus sativus* L.	Iridaceae	Hydro-ethanolic stigma extract (5–20 mg/kg, i.p.)	*In vivo*: Wistar rats (male), BIRI (30 min/24 h)	↓Renal damage, inflammation, and oxidative stress	NR	Mahmoudzadeh et al. (2017)
*Malva sylvestris* L.	Malvaceae	Hydro-ethanolic flower extract (200–600 mg/kg, i.p.)	*In vivo*: Wistar rats (male), BIRI (30 min/24 h)	↓Renal damage, inflammation, and oxidative stress	NR	Najafi et al. (2017)
*Crepidiastrum denticulatum* (Houtt.) Pak & Kawano	Asteraceae	Ethanol leaf extract (75 mg/kg, p.o.)	*In vivo*: C57BL/6 mice (male), BIRI (30 min/48 h)	↓Tubular damage	↑Nrf2/HO-1	Kim et al. (2018)
*Vitis vinifera* L.	Vitaceae	Grape seed extract (0.1 mg/kg, i.v.)	*In vivo*: C57BL/6 mice (male), UIRI/UNX (45 min/48 h)	↓Renal damage	Through PI3K/Akt/eNOS pathway	Ohkita et al. (2019)
*Hydrangea paniculata* Siebold & Zucc.	Hydrangeaceae	Hydroalcoholic extract (30 mg/kg, i.p.)	*In vivo*: Wistar rat (male), BIRI (30 min/24 h)	↓Renal damage, inflammation, and oxidative stress	↓ NF-κB	Yousefi-Manesh et al. (2019)
*Senna tora* (L.) Roxb	Fabaceae	70% ethanol seed extract (100 mg/kg, p.o., 7 days)	*In vivo*: SD rats (male), BIRI (30 min/24 h)	↓Renal damage and oxidative stress	NR	Park et al. (2019)
*Acrocomia crispa* (H.B.K.) C.K.E.	Arecaceae	D-005 lipid extract (25–400 mg/kg, p.o.)	*In vivo*: SD rats (male), BIRI (30 min/24 h)	↓Renal damage and oxidative stress	NR	Oyarzabal-Yera et al. (2019)
*Stevia rebaudiana* (Bertoni) Bertoni	Asteraceae	Methanolic leaf extract (200 mg/kg/day, p.o., 5 weeks)	*In vivo*: SD rats (male), UIRI/UNX (45 min/24 h)	↓Renal damage, oxidative stress, and apoptosis	NR	Elsaid et al. (2019)
*Moringa oleifera* Lam	Moringaceae	Defatted leaf methanol extract (200–400 mg/kg, p.o.)	*In vivo*: Wistar rats (male), UIRI (45 min/24 h)	↓Renal damage and oxidative stress	NR	Akinrinde et al. (2020)
*Phoenix dactylifera* L	Arecaceae	Fruit and seed aqueous or methanolic extracts (4–10 mg/kg, p.o.)	*In vivo*: SD rats (male), UIRI/UNX (45 min/24 h, 48 h, and day 7)	↓Renal damage, oxidative stress, inflammation and apoptosis	↑Nrf2 and caspase-3	Alghamdi et al. (2020)
*Trametes robiniophila* Murrill	Polyporaceae	Proteoglycan-rich extract (41.5% polysaccharides; 6 g/kg, p.o.)	*In vivo*: C57BL/6 mice (male), UIRI (35 min/24 h); *In vitro*: HK-2 cells	↓Tubular injury, apoptosis and ER stress	↑miR-1271; ↓GRP78/CHOP	Zhao and Wu (2020)
*Stachys pilifera* Benth.	Lamiaceae	Ethanolic aerial-part extract (500 mg/kg, p.o.)	*In vivo*: Wistar rats (male), UIRI/UNX (30 min/24 h)	↓Renal damage and oxidative stress	NR	Moslemi et al. (2021)
*Lens culinaris* Medik.	Fabaceae	Seed extract (2–8 mg/100 μL saline/mouse, p.o., 2 weeks)	*In vivo*: ICR mice (male), BIRI (20 min/30 min)	↓Renal damage, oxidative stress, inflammation and apoptosis	↓Fas, caspase-3	(Lee et al., 2021)
*Aronia melanocarpa* (Michx.) Elliott	Rosaceae	Anthocyanin extract (50 mg/kg, p.o., 2 weeks)	*In vivo*: C57BL/6 mice (male), BIRI (30 min/24 h)	↑Renal function and histology	↓TNF-α, IL-6, caspase-9, lipid peroxidation	Li et al. (2021)
*Punica granatum* L	Lythraceae	Standardized fruit extract (30% punicalagin, 5% ellagic acid; 400 mg/kg, p.o.)	*In vivo:* SD rats (male), UIRI/UNX (45 min/24 h)	↓Renal damage, oxidative stress, and inflammation	↓NF-κB	Makled et al. (2021)
*Juglans regia* L.	Juglandaceae	Walnut seed-skin extract (450 mg/kg, p.o.)	*In vivo*: SD rats (female), BIRI (50 min/3 h)	↓Renal damage, oxidative stress, inflammation and apoptosis	↓caspase-4, ↑ eNOS	Askin et al. (2022)
*Agathis robusta* (C.Moore ex F.Muell.) L.A.S.Johnson	Araucariaceae	Ethanolic bark extract (200 mg/kg, p.o.)	*In vivo*: Wistar rats (male), UIRI (60 min/24 h)	↓Renal damage, inflammation, and apoptosis	↓NF-κB, caspase-3, HSP90 and p53	Mohamed et al. (2022c)
*Pistia stratiotes* L.	Araceae	Hydroalcoholic whole-plant extract (100 mg/kg, p.o., 7 days)	*In vivo*: SD rats (male), BIRI (30 min/1 h)	↓Renal damage, oxidative stress, and inflammation	NR	Bhavsar et al. (2023)
*Abuta grandifolia* (Mart.) Sandwith	Menispermaceae	Aqueous stem-leaf extract (400 mg/kg, p.o., 5 days)	*In vivo*: Wistar rats (male), BIRI (30 min/24 h)	↓Renal damage and oxidative stress	NR	de Oliveira et al. (2023)
*Sasa* sp	Poaceae	Alkaline leaf extract (NaOH extract, 0.03–3 g/kg, p.o.)	*In vivo*: ddY mice (male), UIRI (45min/24 h) *In vitro*: NRK-52E cells	↓Renal damage, oxidative stress, and inflammation	NR	Sano et al. (2024)
*Jatropha dioica* Sessé	Euphorbiaceae	Hydroalcoholic extract (300 mg/kg, p.o., 7 days)	*In vivo*: Wistar Rats (male and female), BIRI (45min/24 h)	↓Renal damage and oxidative stress	NR	Rodriguez-Rodriguez et al. (2025)

BIRI, Bilateral ischemia-reperfusion injury; UIRI, Unilateral ischemia-reperfusion injury; UIRI/UNX, Unilateral Ischemia-Reperfusion Injury combined with Uninephrectomy; H/R, Hypoxia/Reoxygenation; HK-2, Human renal tubular epithelial cells; SD rats, Sprague–Dawley rats; NR, not reported.

### 4.8 Others

In addition to the major phytochemical classes discussed above, several other natural metabolites, including quinones, anthraquinones, polysaccharides, sulfur-containing metabolites, nucleoside analogues, glycosides, peptides, and enzyme inhibitors, have shown renoprotective effects in ischemic AKI.

Emodin is one of the more extensively investigated metabolites in this section. Early *in vitro* evidence showed that Emodin protected HK-2 cells against H/R injury by reducing oxidative stress and apoptosis (Chen et al., 2017). Subsequent studies extended these findings to renal I/R models and suggested more specific mitochondrial and apoptotic mechanisms. In one study, emodin was administered to mice at relatively low doses before renal I/R injury and protected against mitochondrial fragmentation, mitochondrial membrane potential loss, oxidative stress, and tubular injury; in HK-2 cells, concentrations below 10 μM were used to avoid cytotoxicity (Wang et al., 2022b). Mechanistically, the protective effect was associated with suppression of CaMKII-mediated DRP1 phosphorylation at Ser616 and inhibition of excessive mitochondrial fission. The use of the CaMKII inhibitor KN93 strengthened the interpretation that CaMKII/DRP1 signaling contributes to this effect, although additional genetic validation of DRP1 would further strengthen causality. Another study showed that emodin reduced p53-mediated tubular apoptosis in renal I/R injury based on network pharmacology and experimental validation (Lu et al., 2023). Overall, emodin has relatively broad evidence, but most studies remain preclinical and mainly assess early injury rather than long-term repair, fibrosis, or AKI-to-CKD transition.

Cordycepin pretreatment improved renal function and histological injury and reduced oxidative stress, inflammatory responses, and apoptosis (Aydin et al., 2020; Han F. et al., 2020). A more recent study showed that cordycepin reduced renal injury, inflammation, apoptosis, and ferroptosis-related changes, with protection associated with inhibition of the p38/JNK MAPK pathway (Chen Q. et al., 2025). Although the consistency across studies supports the renoprotective potential of cordycepin, the mechanistic evidence still relies largely on pathway protein expression and pharmacological observation. Stronger validation using p38/JNK inhibitors, overexpression, or rescue experiments would be required to determine whether this pathway is necessary for its protective effect.

Diosgenin is notable because it was studied not only in acute ischemic AKI but also in the progression from AKI to CKD. Diosgenin was administered after renal injury and improved renal function, reduced tubular injury, inflammatory infiltration, oxidative stress, and renal fibrosis (Chiang et al., 2024). Mechanistically, the study linked diosgenin to suppression of the NOX4/p65 axis. Importantly, the use of NOX4-related siRNA experiments in tubular epithelial cells provided stronger support for the involvement of NOX4/p65 signaling than expression analysis alone. This makes the study relatively translationally relevant because it addresses post-injury intervention and long-term fibrotic progression.

Paeoniflorin provides comparatively strong mechanistic evidence for ferroptosis regulation. In renal I/R mice, paeoniflorin was administered before ischemia at 25, 50, or 100 mg/kg, with ferrostatin-1 used as a positive ferroptosis inhibitor control (Ma et al., 2023). The study showed that paeoniflorin reduced renal dysfunction, tubular injury, inflammatory responses, and ferroptosis-related damage. RNA sequencing suggested that the glutathione pathway and SLC7A11 were involved, and SLC7A11 siRNA in HK-2 cells weakened the anti-ferroptotic effect of paeoniflorin. Therefore, the SLC7A11-dependent ferroptosis mechanism is better supported than studies relying only on GPX4 or SLC7A11 expression changes. However, the treatment was still preventive, and whether paeoniflorin is effective when administered after reperfusion remains unknown.

Honokiol is another metabolite with relatively strong mechanistic validation. Honokiol increased glutathione biosynthesis by upregulating GCLC and GCLM and reduced renal oxidative injury. Its effects were associated with Nrf2 activation through PI3K/Akt and PKC signaling. The use of pharmacological inhibitors and siRNA-mediated gene silencing strengthened the causal interpretation that *de novo* glutathione synthesis contributes to honokiol-mediated protection (Park et al., 2020). Nevertheless, the study focused primarily on antioxidant defense, and long-term renal outcomes and clinical pharmacokinetics were not evaluated.

Allicin has been investigated in more than one renal I/R study, where it appears to exert antioxidant, anti-inflammatory, and anti-apoptotic effects, associated with improved antioxidant capacity, reduced Bax/Bcl-2 imbalance, and inhibition of caspase-3 activation (Shan et al., 2021; Li et al., 2022). However, these mechanisms were mainly inferred from marker expression, without apoptosis-specific rescue experiments or pathway inhibition. Thus, allicin should be described as associated with anti-apoptotic and antioxidant responses rather than as having a definitively established molecular target.

Other metabolites have shown protective effects but generally have less extensive mechanistic validation. Gastrodin pretreatment at 300 mg/kg/day improved renal function, histological damage, oxidative stress, inflammation, and apoptosis in rat renal I/R injury, with protection associated with Nrf2/HO-1 activation (Zheng et al., 2022). Polyacetylene glycoside reduced oxidative stress, inflammatory mediators, and NF-κB activation in tubular epithelial cells and renal ischemic injury models, suggesting anti-inflammatory and antioxidant activity (Zhou Y. et al., 2018). Shikonin attenuated renal I/R injury by suppressing ER stress-induced apoptosis, with protection associated with activation of the Sirt1/Nrf2/HO-1 pathway (Huang Q. et al., 2025). Chitosan oligosaccharide reduced ischemic AKI and later renal fibrosis by alleviating oxidative stress, mitochondrial injury, and excessive ER stress (Yin et al., 2023). Ganoderma lucidum polysaccharide peptide protected against renal I/R injury and H/R injury by reducing oxidative stress, apoptosis, and mitochondrial or ER stress-related damage (Zhong et al., 2015). Apocynin, an NADPH oxidase inhibitor, alleviated renal I/R injury by reducing oxidative stress and inflammation and was associated with restoration of zinc levels and metallothionein expression (Hu et al., 2017). Oleuropein was recently reported to improve renal I/R injury in rats through antioxidant and anti-apoptotic effects (Helvaci-Kurt et al., 2026). Although these studies collectively support the multitarget potential of miscellaneous natural metabolites, most remain descriptive and rely on short-term rodent or tubular cell models.

## 5 Discussion and future directions

Over the past decade, an increasing body of evidence has elucidated the substantial renoprotective effects of natural metabolites in experimental models of ischemic AKI. These metabolites and preparations, which are derived from botanical drugs and other natural sources, encompass flavonoid metabolites, alkaloids, terpenoids, saponins, phenylpropanoid metabolites, polyphenolic metabolites, TCM formulations, and natural extracts, and have been shown to mitigate renal dysfunction and histopathological damage through the coordinated modulation of oxidative stress, inflammation, mitochondrial dysfunction, and various regulated forms of cell death. A distinctive characteristic of natural metabolites is their ability to act on multiple targets and pathways. Despite the wealth of preclinical evidence, several critical limitations must be addressed to facilitate clinical translation.

First, most studies use rodent models with relatively standardized ischemic durations and controlled experimental conditions, which may not adequately replicate the heterogeneity and comorbidities seen in clinical populations, such as advanced age, diabetes, hypertension, or immunosuppression (Shiva et al., 2020). Moreover, large animal models and transplantation-specific models are currently underutilized and should be integrated more extensively to enhance translational relevance. In addition, most studies rely on short-term outcome assessment, whereas long-term consequences such as renal fibrosis, maladaptive repair, and AKI-to-CKD transition remain insufficiently investigated.

Second, mechanistic investigations are often limited to descriptive analyses of protein or mRNA expression changes. Although alterations in signaling pathways such as Nrf2, NF-κB, PI3K/Akt, and MAPK are frequently reported, causal relationships are rarely validated through genetic knockout models, pathway-specific inhibitors, or gain- and loss-of-function approaches. Without such rigorous functional validation, it remains difficult to determine whether these pathways are primary targets or secondary responses. Future studies should emphasize functional verification to strengthen mechanistic credibility. This issue is particularly relevant for several categories, including flavonoid metabolites, where many studies report pathway associations without sufficient causal validation. Although stronger mechanistic support is available for selected flavonoid metabolites such as Scutellarin, Tiliroside, Xanthohumol, Luteoloside, Hydroxysafflor yellow A, and Astilbin, many other flavonoid metabolites still require validation using knockout, knockdown, overexpression, inhibitor, direct target-engagement, reporter, and rescue approaches.

Third, pharmacokinetic challenges represent a major barrier to clinical translation. Many natural metabolites exhibit poor aqueous solubility, low oral bioavailability, rapid metabolism, and limited tissue targeting (Bilia et al., 2019). Long-term safety profiles and potential botanical drug–drug interactions remain insufficiently characterized. Comprehensive pharmacokinetic and toxicological evaluations are essential before advancing toward clinical trials (Liang et al., 2021). The development of advanced delivery systems, including nanoparticles, liposomes, and targeted carriers, may help overcome these limitations by improving stability, bioavailability, and tissue specificity (Si et al., 2026; Zeng et al., 2025). Future studies should also determine dose-response relationships, systemic exposure, renal tissue distribution, and active metabolite profiles, rather than relying only on efficacy endpoints.

Fourth, the timing of administration poses a critical translational challenge. In experimental studies, natural metabolites are frequently administered as preventive or preconditioning agents before ischemia induction, whereas clinical ischemic AKI often develops unexpectedly and is usually recognized only after renal injury or reperfusion has occurred. Therefore, evidence obtained from prophylactic paradigms may be more applicable to predictable ischemic settings, such as kidney transplantation, cardiac surgery, or other planned procedures, but may not fully reflect the therapeutic requirements of unanticipated AKI. Whether metabolites such as resveratrol or baicalin are effective when administered during reperfusion or after the onset of injury, and what constitutes the optimal therapeutic window, remain open questions. Future studies should therefore distinguish clearly between preventive and therapeutic administration, and should incorporate clinically realistic post-injury treatment designs, including administration at defined time points after reperfusion, such as early, delayed, and repeated dosing regimens. In addition, therapeutic efficacy should be assessed not only by short-term changes in serum creatinine, BUN, or histological injury, but also by renal repair, inflammation resolution, fibrosis, and AKI-to-CKD transition. Such designs would provide a more rigorous basis for determining whether natural metabolites and botanical preparations have genuine translational potential beyond experimental preconditioning models.

Several widely studied polyphenolic metabolites discussed in this review, including curcumin, quercetin, resveratrol, luteolin, apigenin, and genistein, may represent potential assay-interfering metabolites *in vitro*. Such metabolites can generate non-specific signals through mechanisms like redox cycling, aggregation, metal chelation, fluorescence interference, or promiscuous protein binding (Bolz et al., 2021). Therefore, protective effects observed in biochemical or cell-based assays—particularly those inferred solely from changes in Nrf2/HO-1, NF-κB, MAPK, PI3K/Akt, or NLRP3 markers—should be interpreted with caution. To reduce the risk of overestimating their specific activity, future studies should ideally incorporate orthogonal assays, concentration-response analysis, target-engagement validation, genetic knockdown/knockout, inhibitor or rescue experiments, and *in vivo* confirmation. Such approaches will help distinguish true renoprotective activity from assay-dependent or non-specific *in vitro* effects.

Despite these challenges, several promising directions may accelerate progress. Integration of multi-omics technologies, including transcriptomics, proteomics, metabolomics, and single-cell sequencing, can provide a systems-level understanding of how natural metabolites modulate complex regulatory networks (Sanches et al., 2024; Cao W et al., 2024). Recent work revealed that astragaloside IV regulates distinct gene clusters in tubular epithelial cells versus endothelial cells, highlighting cell type-specific effects within the kidney microenvironment (Wang et al., 2024b). Such approaches may uncover novel molecular targets and identify key regulatory hubs.

The concept of systems pharmacology offers a valuable framework for understanding the pleiotropic effects of natural metabolites and complex natural preparations. Network-based analyses can elucidate how multiple pathways converge to restore homeostasis and guide rational combination therapies (Pan et al., 2026). Synergistic strategies combining natural metabolites or botanical preparations with established therapeutic agents—such as antioxidants, anti-inflammatory drugs, or immunosuppressants—may enhance protective effects (Karimi et al., 2025; Shen et al., 2025; Topkaraoglu et al., 2026).

Standardization and quality control also warrant attention, particularly for botanical extracts or complex formulations (Chen et al., 2022). Variability in extraction methods, chemical profiles, and batch consistency can influence pharmacological outcomes (Zhou et al., 2025). Establishing standardized preparation protocols and precise chemical characterization, such as HPLC fingerprinting for extracts, is essential for reproducibility and regulatory approval (Xiao et al., 2022). Ultimately, well-designed clinical studies are indispensable for determining therapeutic efficacy and safety in humans. Randomized controlled trials in high-risk populations, such as kidney transplant recipients or patients undergoing major cardiovascular surgery, could provide valuable insights. Biomarker-guided stratification strategies may enable precision-based application of natural metabolites in susceptible individuals, paving the way for personalized renoprotective therapies.

## 6 Conclusion

In summary, plant metabolites represent a promising and mechanistically diverse class of agents for mitigating ischemic AKI. Their ability to modulate oxidative stress, inflammation, and regulated cell death pathways (including ferroptosis, apoptosis, and pyroptosis) positions them as potential network regulators in the pathogenesis of ischemic AKI. Polyphenols, terpenoids, and alkaloids carry the strongest preclinical evidence, largely by activating the Nrf2/HO-1 antioxidant pathway and blocking the proinflammatory NF-κB cascade. However, clinical applicability remains uncertain owing to unresolved issues of bioavailability, pharmacokinetics, toxicity, formulation standardization, herb-drug interactions, reproducibility, and the absence of adequately powered clinical trials. Future research incorporating systems biology, advanced drug delivery technologies, and rigorous clinical evaluations is essential to bridge the gap between experimental findings and therapeutic applications. With continued interdisciplinary collaboration and innovative research, plant metabolites can transition from experimental candidates to clinically relevant adjunctive therapies for ischemic AKI.

## References

[B1] Abou-Hany H. O. Atef H. Said E. Elkashef H. A. Salem H. A. (2018). Crocin reverses unilateral renal ischemia reperfusion injury-induced augmentation of oxidative stress and toll like receptor-4 activity. Environ. Toxicol. Pharmacol. 59, 182–189. 10.1016/j.etap.2018.03.017 29625388

[B2] Ahmadvand H. Yalameha B. Adibhesami G. Nasri M. Naderi N. Babaeenezhad E. (2019). The protective role of gallic acid pretreatment on renal ischemia-reperfusion injury in rats. Rep. Biochem. Mol. Biol. 8 (1), 42–48. 31334287 PMC6590945

[B3] Akinrinde A. S. Oduwole O. Akinrinmade F. J. Bolaji-Alabi F. B. (2020). Nephroprotective effect of methanol extract of Moringa oleifera leaves on acute kidney injury induced by ischemia-reperfusion in rats. Afr. Health. Sci. 20 (3), 1382–1396. 10.4314/ahs.v20i3.44 33402987 PMC7751547

[B4] Alaasam E. R. Janabi A. M. Al-Buthabhak K. M. Almudhafar R. H. Hadi N. R. Alexiou A. (2024). Nephroprotective role of resveratrol in renal ischemia-reperfusion injury: a preclinical study in sprague-Dawley rats. BMC Pharmacol. Toxicol. 25 (1), 82. 10.1186/s40360-024-00809-8 39468702 PMC11520524

[B227] Alechinsky L. Favreau F. Cechova P. Inal S. Faye P.-A. Ory C. (2020). Tannic acid improves renal function recovery after renal warm ischemia–reperfusion in a rat model. Biomolecules 10, 439. 10.3390/biom10030439 32178273 PMC7175177

[B5] Alghamdi M. A. Hussein A. M. Al-Eitan L. N. Elnashar E. Elgendy A. Abdalla A. M. (2020). Possible mechanisms for the renoprotective effects of date palm fruits and seeds extracts against renal ischemia/reperfusion injury in rats. Biomed. Pharmacother. 130, 110540. 10.1016/j.biopha.2020.110540 32763814

[B7] Amini N. Sarkaki A. Dianat M. Mard S. A. Ahangarpour A. Badavi M. (2019). Protective effects of naringin and trimetazidine on remote effect of acute renal injury on oxidative stress and myocardial injury through Nrf-2 regulation. Pharmacol. Rep. 71 (6), 1059–1066. 10.1016/j.pharep.2019.06.007 31604166

[B8] Arslan E. Turk H. Caglayan M. Turkmenoglu T. T. Gonel A. Tayman C. (2021). Protective effects of astaxanthin on nephrotoxicity in rats with induced renovascular occlusion. Comb. Chem. High. Throughput Screen. 24 (8), 1236–1242. 10.2174/1386207323666200914104432 32928081

[B9] Askin S. Askin H. Dursun E. Palabiyik E. Uguz H. Cakmak O. (2022). The hepato-renal protective potential of walnut seed skin extract against acute renal ischemia/reperfusion damage. Cytokine 153, 155861. 10.1016/j.cyto.2022.155861 35306426

[B10] Aydin H. R. Sekerci C. A. Yigit E. Kucuk H. Kocakgol H. Kartal S. (2020). Protective effect of cordycepin on experimental renal ischemia/reperfusion injury in rats. Arch. Ital. Urol. Androl. 92 (4), 340–344. 10.4081/aiua.2020.4.340 33348963

[B11] Bai J. Zhao J. Cui D. Wang F. Song Y. Cheng L. (2018). Protective effect of hydroxysafflor yellow A against acute kidney injury via the TLR4/NF-kappaB signaling pathway. Sci. Rep. 8 (1), 9173. 10.1038/s41598-018-27217-3 29907783 PMC6003992

[B12] Baltaci A. K. Gokbudak H. Baltaci S. B. Mogulkoc R. Avunduk M. C. (2019). The effects of resveratrol administration on lipid oxidation in experimental renal ischemia-reperfusion injury in rats. Biotech. Histochem. 94 (8), 592–599. 10.1080/10520295.2019.1612091 31271315

[B13] Barbar S. D. Wald R. Quenot J. P. (2025). Acute kidney injury: when and how to start renal replacement therapy. Intensive Care Med. 51 (6), 1172–1175. 10.1007/s00134-025-07933-x 40387887

[B14] Baryla M. Skrzycki M. Danielewicz R. Kosieradzki M. Struga M. (2024). Protein biomarkers in assessing kidney quality before transplantation-current status and future perspectives. Int. J. Mol. Med. 54 (6), 107. (Review). 10.3892/ijmm.2024.5431 39370783 PMC11448562

[B15] Beytur L. Korkmaz E. Kose E. Taslidere A. Tekin S. (2025). Myrtenal pretreatment exerts a protective effect against renal ischemia-reperfusion injury in rats. J. Biochem. Mol. Toxicol. 39 (3), e70202. 10.1002/jbt.70202 40025781 PMC11873678

[B16] Bhavsar V. P. Patel A. Vaghasiya J. D. Padhiyar S. Patel T. B. (2023). *Pistia stratiotes* has renoprotective potentials in ischemia reperfusion injury in normal and diabetic rats. Indian J. Pharmacol. 55 (6), 367–375. 10.4103/ijp.ijp_272_23 38174533 PMC10821690

[B17] Bilia A. R. Piazzini V. Risaliti L. Vanti G. Casamonti M. Wang M. (2019). Nanocarriers: a successful tool to increase solubility, stability and optimise bioefficacy of natural constituents. Curr. Med. Chem. 26 (24), 4631–4656. 10.2174/0929867325666181101110050 30381065

[B18] Bolz S. N. Adasme M. F. Schroeder M. (2021). Toward an understanding of pan-assay interference compounds and promiscuity: a structural perspective on binding modes. J. Chem. Inf. Model. 61 (5), 2248–2262. 10.1021/acs.jcim.0c01227 33899463

[B19] Bondi C. D. Hartman H. L. Tan R. J. (2024). NRF2 in kidney physiology and disease. Physiol. Rep. 12 (5), e15961. 10.14814/phy2.15961 38418382 PMC10901725

[B20] Cai F. F. Li D. R. Zhou K. Q. Zhang W. Yang Y. W. (2024). Tiliroside attenuates acute kidney injury by inhibiting ferroptosis through the disruption of NRF2-KEAP1 interaction. Phytomedicine 126, 155407. 10.1016/j.phymed.2024.155407 38340577

[B21] Cakir M. Duzova H. Baysal I. Gul C. C. Kuscu G. Kutluk F. (2017). The effect of hypericum perforatum on kidney ischemia/reperfusion damage. Ren. Fail. 39 (1), 385–391. 10.1080/0886022X.2017.1287734 28209087 PMC6014337

[B22] Cantoni C. Granata S. Bruschi M. Spaggiari G. M. Candiano G. Zaza G. (2020). Recent advances in the role of natural killer cells in acute kidney injury. Front. Immunol. 11, 1484. 10.3389/fimmu.2020.01484 32903887 PMC7438947

[B23] Cao D. Long H. S. Shen X. B. Hu B. Xu S. X. Zhang H. N. (2024). Simultaneous qualitative and quantitative analysis for evaluating constituents of Atractylodis macrocephalae rhizome by UPLC-QTOF-MS. Acta Chromatogr. 36 (4), 333–343. 10.1556/1326.2023.01151

[B24] Cao W. Wu J. Zhao X. Li Z. Yu J. Shao T. (2024). Structural elucidation of an active polysaccharide from Radix Puerariae lobatae and its protection against acute alcoholic liver disease. Carbohydr. Polym. 325, 121565. 10.1016/j.carbpol.2023.121565 38008472

[B25] Caskurlu T. Kanter M. Erboga M. Erboga Z. F. Ozgul M. Atis G. (2016). Protective effect of Nigella sativa on renal reperfusion injury in rat. Iran. J. Kidney Dis. 10 (3), 135–143. 27225721

[B26] Castaneda R. Caceres A. Cruz S. M. Aceituno J. A. Marroquin E. S. Barrios Sosa A. C. (2023). Nephroprotective plant species used in traditional Mayan medicine for renal-associated diseases. J. Ethnopharmacol. 301, 115755. 10.1016/j.jep.2022.115755 36181985

[B27] Chen H. Huang R. S. Yu X. X. Ye Q. Pan L. L. Shao G. J. (2017). Emodin protects against oxidative stress and apoptosis in HK-2 renal tubular epithelial cells after hypoxia/reoxygenation. Exp. Ther. Med. 14 (1), 447–452. 10.3892/etm.2017.4473 28672952 PMC5488603

[B28] Chen D. Q. Feng Y. L. Chen L. Liu J. R. Wang M. Vaziri N. D. (2019). Poricoic acid A enhances melatonin inhibition of AKI-to-CKD transition by regulating Gas6/AxlNFkappaB/Nrf2 axis. Free Radic. Biol. Med. 134, 484–497. 10.1016/j.freeradbiomed.2019.01.046 30716432

[B29] Chen L. Wang S. Yuan H. Yang J. Meng M. Zhan Z. (2022). High-performance thin-layer chromatography (HPTLC) method for analysis of secondary metabolites of *Semiaquilegiae Radix* . Jpc-J. Planar Chromat. 35 (4), 403–410. 10.1007/s00764-022-00194-0

[B30] Chen L. Yuan J. Li H. Ding Y. Yang X. Yuan Z. (2023). Trans-cinnamaldehyde attenuates renal ischemia/reperfusion injury through suppressing inflammation via JNK/p38 MAPK signaling pathway. Int. Immunopharmacol. 118, 110088. 10.1016/j.intimp.2023.110088 37011503

[B31] Chen Y. Li Z. Zhang H. Chen H. Hao J. Liu H. (2024). Mitochondrial metabolism and targeted treatment strategies in ischemic-induced acute kidney injury. Cell Death Discov. 10 (1), 69. 10.1038/s41420-024-01843-5 38341438 PMC10858869

[B32] Chen J. N. Chen C. W. Lv C. Feng R. T. Zhong W. B. Liu Y. G. (2025). Vitexin enhances mitophagy and improves renal ischemia-reperfusion injury by regulating the p38/MAPK pathway. Ren. Fail. 47 (1), 2463572. 10.1080/0886022x.2025.2463572 39961687 PMC11834780

[B33] Chen Q. Guo J. Han S. Wang T. Xia K. Yu B. (2025). Cordycepin alleviates renal ischemia-reperfusion injury by suppressing the p38/JNK signaling pathway. Int. Immunopharmacol. 150, 114264. 10.1016/j.intimp.2025.114264 39954658

[B34] Chen J. Li Y. Zhou P. Zou S. Zhao J. Wu M. (2026). Salvianolic acid C alleviates acute kidney injury by restoring fructose-1,6-bisphosphatase 1-mediated gluconeogenesis. Ren. Fail. 48 (1), 2629902. 10.1080/0886022X.2026.2629902 41795894 PMC12973791

[B35] Chiang C. H. Lan T. Y. Hsieh J. H. Lin S. C. Chen J. W. Chang T. T. (2024). Diosgenin reduces acute kidney injury and ameliorates the progression to chronic kidney disease by modifying the NOX4/p65 signaling pathways. J. Agric. Food Chem. 72 (31), 17444–17454. 10.1021/acs.jafc.4c04183 39074384 PMC11311217

[B37] Chouchani E. T. Pell V. R. Gaude E. Aksentijevic D. Sundier S. Y. Robb E. L. (2014). Ischaemic accumulation of succinate controls reperfusion injury through mitochondrial ROS. Nature 515 (7527), 431–435. 10.1038/nature13909 25383517 PMC4255242

[B38] Christou C. D. Jaradat F. Olsburgh J. Sacks S. Kassimatis T. (2026). Rationale for targeting complement to mitigate renal transplant ischemia-reperfusion injury. J. Am. Soc. Nephrol. 37 (1), 193–207. 10.1681/ASN.0000000826 40694415 PMC12807171

[B39] Chu L. K. Cao X. Wan L. Diao Q. Zhu Y. Kan Y. (2023). Autophagy of OTUD5 destabilizes GPX4 to confer ferroptosis-dependent kidney injury. Nat. Commun. 14 (1), 8393. 10.1038/s41467-023-44228-5 38110369 PMC10728081

[B40] Cui X. Lin L. Sun X. Wang L. Shen R. (2021). Curcumin protects against renal ischemia/reperfusion injury by regulating oxidative stress and inflammatory response. Evid. Based Complement. Altern. Med. 2021, 8490772–8490778. 10.1155/2021/8490772 PMC8605918 34812266

[B41] Dai J. Li C. Zhao L. Guan C. Yang C. Zhang N. (2022). Scutellarin protects the kidney from ischemia/reperfusion injury by targeting Nrf2. Nephrol. Carlt. 27 (8), 690–700. 10.1111/nep.14069 35638402

[B42] Danis E. G. Acar G. Dasdelen D. Solmaz M. Mogulkoc R. Baltaci A. K. (2024). Naringin affects caspase-3, IL-1beta, and HIF-1alpha levels in experimental kidney ischemia-reperfusion in rats. Curr. Pharm. Des. 30 (42), 3339–3349. 10.2174/0113816128324562240816095551 39229980

[B43] de Oliveira B. K. F. de Oliveira Silva E. Ventura S. Vieira G. H. F. de Pina Victoria C. D. Volpini R. A. (2023). Amazonia phytotherapy reduces ischemia and reperfusion injury in the kidneys. Cells 12 (13), 1688. 10.3390/cells12131688 37443721 PMC10341095

[B44] DengG. ZhengB. DouM. GaoY. ZhangX. NiuZ. (2024). Scutellarin alleviates renal ischemia-reperfusion injury by inhibiting the MAPK pathway and pro-inflammatory macrophage polarization. FASEB J. 38 (13), e23769. 10.1096/fj.202302243R 38958951

[B45] Elsaid F. H. Khalil A. A. Ibrahim E. M. Mansour A. Hussein A. M. (2019). Effects of exercise and stevia on renal ischemia/reperfusion injury in rats. Acta Sci. Pol. Technol. Aliment. 18 (3), 317–332. 10.17306/J.AFS.0652 31569913

[B46] Eraslan E. Tanyeli A. Polat E. Yetim Z. (2019). Evodiamine alleviates kidney ischemia reperfusion injury in rats: a biochemical and histopathological study. J. Cell. Biochem. 120 (10), 17159–17166. 10.1002/jcb.28976 31099131

[B47] Erkasap S. Erkasap N. Bradford B. Mamedova L. Uysal O. Ozkurt M. (2017). The effect of leptin and resveratrol on JAK/STAT pathways and Sirt-1 gene expression in the renal tissue of ischemia/reperfusion induced rats. Bratisl. Lek. Listy 118 (8), 443–448. 10.4149/BLL_2017_086 29050480

[B48] Esteras N. Abramov A. Y. (2022). Nrf2 as a regulator of mitochondrial function: energy metabolism and beyond. Free Radic. Biol. Med. 189, 136–153. 10.1016/j.freeradbiomed.2022.07.013 35918014

[B49] Fahim S. A. El-Dessouki A. M. Osama N. Mageed S. S. A. Mahrous M. H. Mohammed R. A. (2026). Natural compounds targeting inflammatory signaling and cell adhesion molecules in ischemic acute kidney injury. Arch. Pharm. Res. 49, 577–635. 10.1007/s12272-026-01601-4 42035370 PMC13222333

[B50] Fan Y. Chen H. Peng H. Huang F. Zhong J. Zhou J. (2017). Molecular mechanisms of curcumin renoprotection in experimental acute renal injury. Front. Pharmacol. 8, 912. 10.3389/fphar.2017.00912 29311922 PMC5733093

[B51] Fan C. R. N. Chen Q. N. Ren J. Y. Yang X. H. Ru J. Zhang H. B. (2020). Notoginsenoside R1 suppresses inflammatory signaling and rescues renal ischemia-reperfusion injury in experimental rats. Med. Sci. Monit. 26, e920442. 10.12659/MSM.920442 32198879 PMC7111146

[B52] Farahani A. Farahani A. Kashfi K. Ghasemi A. (2025). Inducible nitric oxide synthase (iNOS): more than an inducible enzyme? Rethinking the classification of NOS isoforms. Pharmacol. Res. 216, 107781. 10.1016/j.phrs.2025.107781 40389042 PMC12161154

[B53] Feng J. Kong R. R. Xie L. Y. Lu W. H. Zhang Y. L. Dong H. J. (2020). Clemaichinenoside protects renal tubular epithelial cells from hypoxia/reoxygenation injury *in vitro* through activating the Nrf2/HO-1 signalling pathway. Clin. Exp. Pharmacol. Physiol. 47 (3), 495–502. 10.1111/1440-1681.13219 31785117

[B228] Firouzeh G. Samira M. H. Zeinab K. (2025). Rosmarinic acid: a promising agent for male rats’ renal protection against ischemia/reperfusion injury. Mol. Biol. Rep. 52, 325. 10.1007/s11033-025-10422-5 40106003

[B54] Gao D. Jing S. Zhang Q. Wu G. (2018). Pterostilbene protects against acute renal ischemia reperfusion injury and inhibits oxidative stress, inducible nitric oxide synthase expression and inflammation in rats via the Toll-like receptor 4/nuclear factor-kappaB signaling pathway. Exp. Ther. Med. 15 (1), 1029–1035. 10.3892/etm.2017.5479 29403551 PMC5780844

[B55] Geng X. Fu Z. Geng G. Chi K. Liu C. Hong H. (2024). Astilbin improves the therapeutic effects of mesenchymal stem cells in AKI-CKD mice by regulating macrophage polarization through PTGS2-mediated pathway. Stem Cell Res. Ther. 15 (1), 427. 10.1186/s13287-024-04025-3 39543734 PMC11566621

[B56] Gong J. Wang X. (2018). Schisantherin A protects renal tubular epithelial cells from hypoxia/reoxygenation injury through the activation of PI3K/Akt signaling pathway. J. Biochem. Mol. Toxicol. 32 (7), e22160. 10.1002/jbt.22160 29785781

[B57] Granata S. Votrico V. Spadaccino F. Catalano V. Netti G. S. Ranieri E. (2022). Oxidative stress and ischemia/reperfusion injury in kidney transplantation: focus on ferroptosis, mitophagy and new antioxidants. Antioxidants (Basel) 11 (4), 769. 10.3390/antiox11040769 35453454 PMC9024672

[B58] Guler M. C. Akpinar E. Tanyeli A. Comakli S. Bayir Y. (2023). Costunolide prevents renal ischemia-reperfusion injury in rats by reducing autophagy, apoptosis, inflammation, and DNA damage. Iran. J. Basic Med. Sci. 26 (10), 1168–1176. 10.22038/IJBMS.2023.71779.15596 37736519 PMC10510491

[B60] Han F. Dou M. Wang Y. X. Xu C. X. Li Y. Ding X. M. (2020). Cordycepin protects renal ischemia/reperfusion injury through regulating inflammation, apoptosis, and oxidative stress. Acta Bioch. Biophys. Sin. 52 (2), 125–132. 10.1093/abbs/gmz145 31951250

[B61] Han S. J. Lovaszi M. Kim M. D'Agati V. Hasko G. Lee H. T. (2020). P2X4 receptor exacerbates ischemic AKI and induces renal proximal tubular NLRP3 inflammasome signaling. FASEB J. 34 (4), 5465–5482. 10.1096/fj.201903287R 32086866 PMC7136150

[B62] Han L. Chen A. Liu L. Wang F. (2022). Leonurine preconditioning attenuates ischemic acute kidney injury in rats by promoting Nrf2 nuclear translocation and suppressing TLR4/NF-kappaB pathway. Chem. Pharm. Bull. (Tokyo) 70 (1), 66–73. 10.1248/cpb.c21-00740 34980736

[B63] Havasi A. Borkan S. C. (2011). Apoptosis and acute kidney injury. Kidney Int. 80 (1), 29–40. 10.1038/ki.2011.120 21562469 PMC4625984

[B238] Helvaci-Kurt N. Kural A. Sari-Ak D. Topkaraoglu S. Misirli D. Tavukcuoglu O. (2026). Protective effects of oleuropein isolated from Olea europaea L. on renal ischemia/reperfusion injury in rats: insights into antioxidant and anti-apoptotic pathways. Phytomedicine 150, 157691. 10.1016/j.phymed.2025.157691 41401586

[B64] Hong X. Zhao X. Wang G. Zhang Z. Pei H. Liu Z. (2017). Luteolin treatment protects against renal ischemia-reperfusion injury in rats. Mediat. Inflamm. 2017, 9783893. 10.1155/2017/9783893 29358852 PMC5735687

[B65] Hosohata K. Jin D. Takai S. (2021). Glaucocalyxin A ameliorates hypoxia/reoxygenation-induced injury in human renal proximal tubular epithelial cell line HK-2 cells. Int. J. Mol. Sci. 23 (1), 446. 10.3390/ijms23010446 35008870 PMC8745506

[B66] Hu B. Wu Y. Tong F. Liu J. Shen X. Shen R. (2017). Apocynin alleviates renal ischemia/reperfusion injury through regulating the level of zinc and metallothionen. Biol. Trace Elem. Res. 178 (1), 71–78. 10.1007/s12011-016-0904-z 27909865

[B233] Hu X.-W. Wang Y.-Q. Li X.-Y. Shen X.-Y. Shan R.-R. Yu J.-T. (2026). Lycopene protects against acute kidney injury by suppressing PARP/NOTCH-mediated inflammation. Int. Immunopharmacol. 171, 116136. 10.1016/j.intimp.2025.116136 41481957

[B67] Huang F. Wang X. Xiao G. Xiao J. (2022). Loganin exerts a protective effect on ischemia-reperfusion-induced acute kidney injury by regulating JAK2/STAT3 and Nrf2/HO-1 signaling pathways. Drug Dev. Res. 83 (1), 150–157. 10.1002/ddr.21853 34189758

[B68] Huang R. Zhang C. Xiang Z. Lin T. Ling J. Hu H. (2024). Role of mitochondria in renal ischemia-reperfusion injury. FEBS J. 291 (24), 5365–5378. 10.1111/febs.17130 38567754

[B69] Huang L. Tang Q. Li G. Hou Y. Chen S. Yao Q. (2025). Targeting ALOX5 ameliorates renal tubular injury in ischemia-reperfusion-induced acute kidney injury via inhibition of ferroptosis. Commun. Biol. 8 (1), 1403. 10.1038/s42003-025-08803-4 41028852 PMC12484620

[B70] Huang Q. Shi Z. Zheng D. Chen H. Huang Q. (2025). Shikonin inhibits endoplasmic reticulum stress-induced apoptosis to attenuate renal ischemia/reperfusion injury by activating the Sirt1/Nrf2/HO-1 pathway. Kidney Blood Press. Res. 50 (1), 131–146. 10.1159/000542417 39662059 PMC11844683

[B71] Huo A. Wang F. (2024). Berberine alleviates ischemia reperfusion injury induced AKI by regulation of intestinal microbiota and reducing intestinal inflammation. BMC Complement. Med. Ther. 24 (1), 66. 10.1186/s12906-023-04323-y 38291383 PMC10826000

[B73] Jiang G. S. Xin R. Yuan W. D. Zhang L. X. Meng X. H. Sun W. N. (2020). Ligustrazine ameliorates acute kidney injury through downregulation of NOD2-mediated inflammation. Int. J. Mol. Med. 45 (3), 731–742. 10.3892/ijmm.2020.4464 31985025 PMC7015130

[B74] Jiao H. Zhang M. J. Chen L. L. Zhang Z. R. (2025). Traditional Chinese medicine targeting the TGF-β/Smad signaling pathway as a potential therapeutic strategy for renal fibrosis. Front. Pharmacol. 16, 1513329. 10.3389/fphar.2025.1513329 40463902 PMC12130906

[B75] Kalbolandi S. M. Gorji A. V. Babaahmadi-Rezaei H. Mansouri E. (2019). Luteolin confers renoprotection against ischemia-reperfusion injury via involving Nrf2 pathway and regulating miR320. Mol. Biol. Rep. 46 (4), 4039–4047. 10.1007/s11033-019-04853-0 31089916

[B76] Kapisiz A. Kaya C. Eryilmaz S. Karabulut R. Turkyilmaz Z. Inan M. A. (2024). Protective effects of lupeol in rats with renal ischemia-reperfusion injury. Exp. Ther. Med. 28 (2), 313. 10.3892/etm.2024.12602 38911048 PMC11190881

[B77] Karimi S. Koohpeyma F. Khorsandi L. Panahi A. Rezaei-Tazangi F. Fakhredini F. (2025). Comparative analysis of curcumin and metformin effects on the kidney in a model of unilateral nephrectomy and ischemia-reperfusion injury. Rep. Biochem. Mol. Biol. 13 (4), 579–589. 10.61186/rbmb.13.4.579 40842894 PMC12367211

[B78] Kim J. S. Jang H. J. Jeong E. K. Kim S. S. Kim Y. M. Lee J. H. (2018). Crepidiastrum denticulatum extract ameliorates kidney ischemia-reperfusion injury in mice. Transpl. Proc. 50 (4), 1160–1166. 10.1016/j.transproceed.2018.02.048 29731086

[B232] Kisaoglu A. Kose E. Yilmaz N. Tanbek K. Yildiz A. Yilmaz U. (2024). Investigation of the effect of astaxanthin on autophagy in renal ischemia-reperfusion modeled rats. Medeni Med. J. 39, 101–108. 10.4274/MMJ.galenos.2024.27243 38940481 PMC11572265

[B79] Ko G. J. Jang H. R. Huang Y. Womer K. L. Liu M. Higbee E. (2011). Blocking fas ligand on leukocytes attenuates kidney ischemia-reperfusion injury. J. Am. Soc. Nephrol. 22 (4), 732–742. 10.1681/ASN.2010010121 21436290 PMC3065228

[B80] Kuang B. C. Wang Z. H. Hou S. H. Zhang J. Wang M. Q. Zhang J. S. (2023). Methyl eugenol protects the kidney from oxidative damage in mice by blocking the Nrf2 nuclear export signal through activation of the AMPK/GSK3beta axis. Acta Pharmacol. Sin. 44 (2), 367–380. 10.1038/s41401-022-00942-2 35794373 PMC9889399

[B235] Lee S.-O. Chun S. Y. Lee E. Kim B. Yoon B. Gil H. (2021). Renal protective effect of beluga lentil pretreatment for ischemia-reperfusion injury. BioMed Res. Int. 2021, 6890679. 10.1155/2021/6890679 33604384 PMC7868138

[B81] Lee L. E. Doke T. Mukhi D. Susztak K. (2024). The key role of altered tubule cell lipid metabolism in kidney disease development. Kidney Int. 106 (1), 24–34. 10.1016/j.kint.2024.02.025 38614389 PMC11193624

[B82] Li K. Gong X. Kuang G. Jiang R. Wan J. Wang B. (2016). Sesamin protects against renal ischemia reperfusion injury by promoting CD39-adenosine-A2AR signal pathway in mice. Am. J. Transl. Res. 8 (5), 2245–2254. 27347331 PMC4891436

[B83] Li J. Li L. Wang S. Zhang C. Zheng L. Jia Y. (2018). Resveratrol alleviates inflammatory responses and oxidative stress in rat kidney ischemia-reperfusion injury and H_2_O_2_-induced NRK-52E cells via the Nrf2/TLR4/NF-kappaB pathway. Cell. Physiol. Biochem. 45 (4), 1677–1689. 10.1159/000487735 29490296

[B84] Li H. Chen W. Chen Y. Zhou Q. Xiao P. Tang R. (2019). Neferine attenuates acute kidney injury by inhibiting NF-kappaB signaling and upregulating klotho expression. Front. Pharmacol. 10, 1197. 10.3389/fphar.2019.01197 31680971 PMC6804424

[B85] Li Q. P. Wei R. B. Yang X. Zheng X. Y. Su T. Y. Huang M. J. (2019). Protective effects and mechanisms of shenhua tablet on toll-like receptors in rat model of renal ischemia-reperfusion injury. Chin. J. Integr. Med. 25 (1), 37–44. 10.1007/s11655-017-2756-6 28466227

[B86] Li C. Zheng Z. Xie Y. Zhu N. Bao J. Yu Q. (2020). Protective effect of taraxasterol on ischemia/reperfusion-induced acute kidney injury via inhibition of oxidative stress, inflammation, and apoptosis. Int. Immunopharmacol. 89 (Pt A), 107169. 10.1016/j.intimp.2020.107169 33183976

[B87] Li L. Li J. Xu H. Zhu F. Li Z. Lu H. (2021). The protective effect of anthocyanins extracted from aronia melanocarpa berry in renal ischemia-reperfusion injury in mice. Mediat. Inflamm. 2021, 7372893. 10.1155/2021/7372893 PMC7846408 33551679

[B88] Li M. Ning J. Huang H. Jiang S. Zhuo D. (2022). Allicin protects against renal ischemia-reperfusion injury by attenuating oxidative stress and apoptosis. Int. Urol. Nephrol. 54 (7), 1761–1768. 10.1007/s11255-021-03014-2 34825305 PMC9184421

[B89] Li C. Yu Y. Zhu S. Hu Y. Ling X. Xu L. (2024). The emerging role of regulated cell death in ischemia and reperfusion-induced acute kidney injury: current evidence and future perspectives. Cell Death Discov. 10 (1), 216. 10.1038/s41420-024-01979-4 38704372 PMC11069531

[B90] Li J. Y. Feng Y. H. Li Y. X. He P. Y. Zhou Q. Y. Tian Y. P. (2024). Ferritinophagy: a novel insight into the double-edged sword in ferritinophagy-ferroptosis axis and human diseases. Cell Prolif. 57 (7), e13621. 10.1111/cpr.13621 38389491 PMC11216947

[B91] Li Y. Ma S. Wang Z. Shi M. Zeng R. Yao Y. (2024). Gclc as a marker for injured distal nephron in ischemia-reperfusion induced acute kidney injury. J. Inflamm. Res. 17, 527–540. 10.2147/JIR.S451402 38313210 PMC10838515

[B92] Li H. Wang X. Deng D. Lv S. Huang L. Wang X. (2025). Advances in understanding the role of mitochondria in renal ischemia-reperfusion injury. Clin. Exp. Nephrol. 29 (12), 1685–1698. 10.1007/s10157-025-02727-3 40627278 PMC12660473

[B93] Liang D. Wu Z. Liu Y. Li C. Li X. Yang B. (2021). HPLC-MS/MS-mediated analysis of the pharmacokinetics, bioavailability, and tissue distribution of schisandrol B in rats. Int. J. Anal. Chem. 2021, 8862291. 10.1155/2021/8862291 33679983 PMC7929678

[B94] Liao Y. Lin X. Li J. Tan R. Zhong X. Wang L. (2021). Nodakenin alleviates renal ischaemia-reperfusion injury via inhibiting reactive oxygen species-induced NLRP3 inflammasome activation. Nephrol. Carlt. 26 (1), 78–87. 10.1111/nep.13781 32902019

[B95] Lin Y. Sheng M. Ding Y. Zhang N. Song Y. Du H. (2018). Berberine protects renal tubular cells against hypoxia/reoxygenation injury via the Sirt1/p53 pathway. J. Nat. Med. 72 (3), 715–723. 10.1007/s11418-018-1210-1 29680964

[B96] Lin Y. Xu L. Lin H. Cui W. Jiao Y. Wang B. (2024). Network pharmacology and experimental validation to investigate the mechanism of Nao-Ling-Su capsule in the treatment of ischemia/reperfusion-induced acute kidney injury. J. Ethnopharmacol. 326, 117958. 10.1016/j.jep.2024.117958 38395179

[B98] Liu F. H. Ni W. J. Wang G. K. Zhang J. J. (2016). Protective role of curcumin on renal ischemia reperfusion injury via attenuating the inflammatory mediators and Caspase-3. Cell. Mol. Biol. (Noisy-le-grand) 62 (11), 95–99. 27755959

[B99] Liu Q. Liang X. B. Liang M. T. Qin R. B. Qin F. X. Wang X. L. (2020). Ellagic acid ameliorates renal ischemic-reperfusion injury through NOX4/JAK/STAT signaling pathway. Inflammation 43 (1), 298–309. 10.1007/s10753-019-01120-z 31768706

[B100] Liu Y. Song X. Shen X. Xiong Y. Liu L. Yang Y. (2022). A rapid and efficient strategy for quality control of clinopodii herba encompassing optimized ultrasound-assisted extraction coupled with sensitive variable wavelength detection. Molecules 27 (14), 4418. 10.3390/molecules27144418 35889291 PMC9316769

[B101] Liu J. Sun X. Liang J. Song S. (2025). Eugenol alleviates renal ischemia-reperfusion injury induced-endoplasmic reticulum stress via activating Sestrin2. Clin. (Sao Paulo) 80, 100627. 10.1016/j.clinsp.2025.100627 PMC11985136 40138864

[B102] Liu X. Ning K. H. Li J. C. Yan X. D. Yan J. H. Zhao X. Y. (2025). Sodium aescinate protects renal ischemia-reperfusion and pyroptosis through AKT/NLRP3 signaling pathway. Ren. Fail. 47 (1), 2488140. 10.1080/0886022x.2025.2488140 40260531 PMC12016278

[B103] Lotfipour Nasudivar S. Pedrera L. Garcia-Saez A. J. (2024). Iron-induced lipid oxidation alters membrane mechanics favoring permeabilization. Langmuir 40 (47), 25061–25068. 10.1021/acs.langmuir.4c03294 39532309 PMC11603768

[B104] Lu J. Yi Y. Pan R. Zhang C. Han H. Chen J. (2018). Berberine protects HK-2 cells from hypoxia/reoxygenation induced apoptosis via inhibiting SPHK1 expression. J. Nat. Med. 72 (2), 390–398. 10.1007/s11418-017-1152-z 29260413

[B105] Lu F. Lan Z. Xin Z. He C. Guo Z. Xia X. (2020). Emerging insights into molecular mechanisms underlying pyroptosis and functions of inflammasomes in diseases. J. Cell. Physiol. 235 (4), 3207–3221. 10.1002/jcp.29268 31621910 PMC13482838

[B106] Lu H. Xie D. Qu B. Li M. He Y. Liu W. (2023). Emodin prevents renal ischemia-reperfusion injury via suppression of p53-mediated cell apoptosis based on network pharmacology. Heliyon 9 (5), e15682. 10.1016/j.heliyon.2023.e15682 37215853 PMC10195913

[B107] Lu J. B. Zhang X. F. Chen K. S. (2025). Molecular mechanisms underlying endoplasmic reticulum stress-triggered apoptosis induced by exopolysaccharide in WEHI-3 mouse myeloid leukemic cells. J. Carbohyd. Chem. 44 (4-6), 231–251. 10.1080/07328303.2025.2528127

[B108] Luo S. Chen Y. Ma X. Tian F. He X. (2025). Resveratrol aggravated H2O2-induced the HK-2 cell damage by inhibiting AKT phosphorylation. PLoS One 20 (7), e0327135. 10.1371/journal.pone.0327135 40743123 PMC12312898

[B237] Ma L. Liu X. Zhang M. Zhou L. Jiang L. Gao L. (2023). Paeoniflorin alleviates ischemia/reperfusion-induced acute kidney injury by inhibiting Slc7a11-mediated ferroptosis. Int. Immunopharmacol. 116, 109754. 10.1016/j.intimp.2023.109754 36753983

[B109] Mahmoudzadeh L. Najafi H. Ashtiyani S. C. Yarijani Z. M. (2017). Anti-inflammatory and protective effects of saffron extract in ischaemia/reperfusion-induced acute kidney injury. Nephrol. Carlt. 22 (10), 748–754. 10.1111/nep.12849 27381453

[B110] Mahtal N. Lenoir O. Tinel C. Anglicheau D. Tharaux P. L. (2022). MicroRNAs in kidney injury and disease. Nat. Rev. Nephrol. 18 (10), 643–662. 10.1038/s41581-022-00608-6 35974169

[B111] Makled M. N. El-Awady M. S. Abdel-Aziz R. R. Shehab El-Din A. B. Ammar E. M. Gameil N. M. (2021). Pomegranate extract ameliorates renal ischemia/reperfusion injury in rats via suppressing NF-kappaB pathway. Hum. Exp. Toxicol. 40 (12_Suppl. l), S573–S582. 10.1177/09603271211041998 34802289

[B112] Malathi H. Khandelwal G. Gayathri S. Sahoo S. Sharma S. (2025). Toll-like receptors in kidney ischemia-reperfusion injury: modulating macrophage responses for therapeutic insights. Pathol. Res. Pract. 269, 155940. 10.1016/j.prp.2025.155940 40174275

[B113] Mao H. Wang L. Xiong Y. Jiang G. Liu X. (2022). Fucoxanthin attenuates oxidative damage by activating the Sirt1/Nrf2/HO-1 signaling pathway to protect the kidney from ischemia-reperfusion injury. Oxid. Med. Cell. Longev. 2022, 7444430. 10.1155/2022/7444430 35126819 PMC8816562

[B114] Martinez-Rojas M. A. Balcazar H. Ponce-Nava M. S. Gonzalez-Soria I. Marquina-Castillo B. Perez-Villalva R. (2024). A short treatment with resveratrol after a renal ischaemia-reperfusion injury prevents maladaptive repair and long-term chronic kidney disease in rats. J. Physiol. 602 (8), 1835–1852. 10.1113/JP285979 38529522

[B115] Mohamed M. E. Elmorsy M. A. Younis N. S. (2022a). Renal ischemia/wreperfusion mitigation via geraniol: the role of Nrf-2/HO-1/NQO-1 and TLR2,4/MYD88/NFkappaB pathway. Antioxidants (Basel) 11 (8), 1568. 10.3390/antiox11081568 36009287 PMC9405463

[B116] Mohamed M. E. Kandeel M. Abd El-Lateef H. M. El-Beltagi H. S. Younis N. S. (2022b). The protective effect of anethole against renal ischemia/reperfusion: the role of the TLR2,4/MYD88/NFkappaB pathway. Antioxidants (Basel) 11 (3), 535. 10.3390/antiox11030535 35326185 PMC8944622

[B117] Mohamed M. E. Tawfeek N. Elbaramawi S. S. Elbatreek M. H. Fikry E. (2022c). Agathis robusta bark extract protects from renal ischemia-reperfusion injury: phytochemical, in silico and in vivo studies. Pharm. (Basel) 15 (10), 1270. 10.3390/ph15101270 PMC9610891 36297382

[B118] Mohammadi M. Najafi H. Mohamadi Yarijani Z. Vaezi G. Hojati V. (2020). Protective effect of piperine in ischemia-reperfusion induced acute kidney injury through inhibition of inflammation and oxidative stress. J. Tradit. Complement. Med. 10 (6), 570–576. 10.1016/j.jtcme.2019.07.002 33134133 PMC7588331

[B119] Moslemi Z. Gheitasi I. Doustimotlagh A. H. (2021). Protective effect of hydroalcoholic extract of stachys pilifera on oxidant-antioxidant status in renal ischemia/reperfusion injuries in male rats. J. Toxicol. 2021, 6646963–6646968. 10.1155/2021/6646963 33574840 PMC7864747

[B120] Na S. W. Jang Y. J. Hong M. H. Yoon J. J. Lee H. S. Kim H. Y. (2021). Protective effect of Joa-Gui Em through the improvement of the NLRP3 and TLR4/NF-κb signaling by ischemia/reperfusion-induced acute renal failure rats. Evid. Based Complement. Altern. Med. 2021, 7178868. 10.1155/2021/7178868 PMC8177998 34135984

[B121] Najafi H. Mohamadi Yarijani Z. Changizi-Ashtiyani S. Mansouri K. Modarresi M. Madani S. H. (2017). Protective effect of Malva sylvestris L. extract in ischemia-reperfusion induced acute kidney and remote liver injury. PLoS One 12 (11), e0188270. 10.1371/journal.pone.0188270 29155898 PMC5695808

[B122] Nasrallah H. Aissa I. Slim C. Boujbiha M. A. Zaouali M. A. Bejaoui M. (2020). Effect of oleuropein on oxidative stress, inflammation and apoptosis induced by ischemia-reperfusion injury in rat kidney. Life Sci. 255, 117833. 10.1016/j.lfs.2020.117833 32450167

[B123] Norgard M. O. Svenningsen P. (2023). Acute kidney injury by ischemia/reperfusion and extracellular vesicles. Int. J. Mol. Sci. 24 (20), 15312. 10.3390/ijms242015312 37894994 PMC10607034

[B124] Ohkita M. Hayashi H. Ito K. Shigematsu N. Tanaka R. Tsutsui H. (2019). Preventive effects of Grape Extract on ischemia/reperfusion-induced acute kidney injury in mice. Biol. Pharm. Bull. 42 (11), 1883–1890. 10.1248/bpb.b19-00462 31685770

[B125] Ostermann M. Lumlertgul N. Jeong R. See E. Joannidis M. James M. (2025). Acute kidney injury. Lancet 405 (10474), 241–256. 10.1016/S0140-6736(24)02385-7 39826969

[B126] Oyarzabal-Yera A. Rodriguez-Salgueiro S. Merino-Garcia N. Ocana-Napoles L. Gonzalez-Nunez L. Mena-Valdes L. (2019). Protective effects of D-005, a lipid extract from Acrocomia crispa fruits, against ischemia/reperfusion-induced acute kidney injury in rats. Kidney Res. Clin. Pract. 38 (4), 462–471. 10.23876/j.krcp.19.053 31826388 PMC6913585

[B127] Ozhan O. Ekici C. Ates B. Yildiz A. Balcioglu S. Vardi N. (2026). N-acetyl cysteine amide mitigates oxidative stress and apoptosis in a rat model of renal ischemia-reperfusion injury. Sci. Rep. 16 (1), 6323. 10.1038/s41598-026-37274-8 41593161 PMC12905359

[B128] Ozturk H. Cetinkaya A. Yilmaz F. Ozturk H. (2017). Protective effect of oxymatrine against renal ischemia/reperfusion injury in rats. Bratisl. Lek. Listy 118 (4), 217–222. 10.4149/BLL_2017_043 28471232

[B230] Ozbek Sebin S. Gulakar B. Tanyeli A. Guler M. C. Erbas E. Baser S. (2025). Alpha-pinene mitigates renal ischemia–reperfusion injury via anti-oxidant, anti-inflammatory, anti-apoptotic effects and favorable eNOS/iNOS shifts in rats. Toxicon. 269, 108637. 10.1016/j.toxicon.2025.108637 41197880

[B129] Pan Y. Zhu J. (2026). Mitochondria in renal ischemia-reperfusion injury: from mechanisms to therapeutics. Biomedicines 14 (2), 310. 10.3390/biomedicines14020310 41751208 PMC12937674

[B130] Pan C. Zhao H. Cai X. Wu M. Qin B. Li J. (2024). The connection between autophagy and ferroptosis in AKI: recent advances regarding selective autophagy. Ren. Fail. 46 (2), 2379601. 10.1080/0886022X.2024.2379601 39099238 PMC11302486

[B131] Pan M. Yan C. Zhang J. Li X. Sun L. Mao C. (2026). Exploring the potential mechanism of Ru-Pi-Ning Granules against mammary gland hyperplasia based on UPLC-Q-TOF-MS and network pharmacology. Biomed. Chromatogr. 40 (3), e70356. 10.1002/bmc.70356 41549505

[B132] Pang Y. Zhang P. C. Lu R. R. Li H. L. Li J. C. Fu H. X. (2020). Andrade-oliveira salvianolic acid B modulates caspase-1-mediated pyroptosis in renal ischemia-reperfusion injury via Nrf2 pathway. Front. Pharmacol. 11, 541426. 10.3389/fphar.2020.541426 33013384 PMC7495093

[B133] Park S. I. Yun H. C. Kang S. W. Kim S. K. Lee H. Park M. S. (2019). Effect of seed of Cassia tora extract in the prevention of remote renal reperfusion injury. Transpl. Proc. 51 (8), 2833–2837. 10.1016/j.transproceed.2019.04.082 31439329

[B236] Park E. J. Dusabimana T. Je J. Jeong K. Yun S. P. Kim H. J. (2020). Honokiol protects the kidney from renal ischemia and reperfusion injury by upregulating the glutathione biosynthetic enzymes. Biomedicines 8, 352. 10.3390/biomedicines8090352 32942603 PMC7555803

[B134] Peng J. Ren X. Lan T. Chen Y. Shao Z. Yang C. (2016). Renoprotective effects of ursolic acid on ischemia/reperfusion-induced acute kidney injury through oxidative stress, inflammation and the inhibition of STAT3 and NF-kappaB activities. Mol. Med. Rep. 14 (4), 3397–3402. 10.3892/mmr.2016.5654 27573738

[B135] Piko N. Bevc S. Hojs R. Ekart R. (2023). The role of oxidative stress in kidney injury. Antioxidants (Basel) 12 (9), 1772. 10.3390/antiox12091772 37760075 PMC10525550

[B136] Qin X. Liu J. Zhao X. Wang L. Liu X. Chen Z. (2025). Platycodin D attenuates diabetic renal ischemia/reperfusion injury by enhancing mitophagy and suppressing MAPK/NF-kappaB signaling activation. Biochim. Biophys. Acta Mol. Basis. Dis. 1871 (8), 168026. 10.1016/j.bbadis.2025.168026 40848991

[B231] Qiu Q. Zhou Y. Xia K. Yan Z. Hui Y. Chen Z. (2026). Luteoloside attenuates renal ischemia–reperfusion injury by suppressing ferroptosis through disruption of the KEAP1–NRF2 interaction. Free Radic. Biol. Med. 248, 177–193. 10.1016/j.freeradbiomed.2026.02.055 41730482

[B137] Reid S. Scholey J. W. (2021). Recent approaches to targeting canonical NFkappaB signaling in the early inflammatory response to renal IRI. J. Am. Soc. Nephrol. 32 (9), 2117–2124. 10.1681/ASN.2021010069 34108233 PMC8729839

[B138] Ren K. Jin C. Ma P. Ren Q. Jia Z. Zhu D. (2016). Ginsenoside Rd alleviates mouse acute renal ischemia/reperfusion injury by modulating macrophage phenotype. J. Ginseng Res. 40 (2), 196–202. 10.1016/j.jgr.2015.12.003 27158241 PMC4845042

[B139] Ren L. Zhao Y. Ji X. Li W. Jiang W. Li Q. (2024). The therapeutic effect of Picroside II in renal ischemia-reperfusion induced acute kidney injury: an experimental study. Eur. J. Pharmacol. 967, 176391. 10.1016/j.ejphar.2024.176391 38325794

[B140] Rodriguez-Rodriguez D. R. Mendoza-Hernandez O. H. Cordero-Perez P. Rivas-Galindo V. M. Moreno-Pena D. P. Tijerina-Marquez R. (2025). Nephroprotective and antioxidant effects of Jatropha dioica extract against ischemia-reperfusion injury in Wistar rats. Int. J. Mol. Sci. 26 (5), 1838. 10.3390/ijms26051838 40076464 PMC11899379

[B142] Sallabi S. M. Lubbad L. Toumi H. R. Hammad W. F. Sudhadevi M. Rasheed J. A. (2025). The effect of nuciferine on the renal dysfunction following ischemia-reperfusion injury. PLoS One 20 (3), e0320320. 10.1371/journal.pone.0320320 40138328 PMC11940656

[B143] Sampaio T. L. Menezes R. R. da Costa M. F. Meneses G. C. Arrieta M. C. Chaves Filho A. J. (2016). Nephroprotective effects of (-)-alpha-bisabolol against ischemic-reperfusion acute kidney injury. Phytomedicine 23 (14), 1843–1852. 10.1016/j.phymed.2016.11.008 27912887

[B144] Sanches P. H. G. de Melo N. C. Porcari A. M. de Carvalho L. M. (2024). Integrating molecular perspectives: strategies for comprehensive multi-omics integrative data analysis and machine learning applications in transcriptomics, proteomics, and metabolomics. Biol. (Basel) 13 (11), 848. 10.3390/biology13110848 PMC11592251 39596803

[B145] Sano M. Koseki Y. Shibata K. Fujisawa T. Nobe K. (2024). Therapeutic effects of the alkaline extract of leaves of Sasa sp. and elucidation of its mechanism in acute kidney injury. J. Pharmacol. Sci. 154 (3), 148–156. 10.1016/j.jphs.2024.01.004 38395515

[B146] Shan Y. Chen D. Hu B. Xu G. Li W. Jin Y. (2021). Allicin ameliorates renal ischemia/reperfusion injury via inhibition of oxidative stress and inflammation in rats. Biomed. Pharmacother. 142, 112077. 10.1016/j.biopha.2021.112077 34426252

[B147] Shan R. R. Yu J. T. Zhang S. F. Xie M. M. Hou R. Xie C. Y. (2024). Madecassoside alleviates acute kidney injury by regulating JNK-mediated oxidative stress and programmed cell death. Phytomedicine 123, 155252. 10.1016/j.phymed.2023.155252 38056145

[B148] Shao G. He J. Meng J. Ma A. Geng X. Zhang S. (2021). Ganoderic acids prevent renal ischemia reperfusion injury by inhibiting inflammation and apoptosis. Int. J. Mol. Sci. 22 (19), 10229. 10.3390/ijms221910229 34638569 PMC8508562

[B149] Shen X. Zheng Y. Yang H. Liu L. Yu L. Zhang Y. (2025). Combination treatment of timosaponin BII and pirfenidone attenuated pulmonary fibrosis through anti-inflammatory and anti-fibrotic process in rodent pulmonary fibrosis model and cellular epithelial-mesenchymal transition model. Molecules 30 (8), 1821. 10.3390/molecules30081821 40333870 PMC12029700

[B150] Shiva N. Sharma N. Kulkarni Y. A. Mulay S. R. Gaikwad A. B. (2020). Renal ischemia/reperfusion injury: an insight on *in vitro* and in vivo models. Life Sci. 256, 117860. 10.1016/j.lfs.2020.117860 32534037

[B152] Shyam M. Pal A. Dey D. J K. Sharma V. Wadhwa J. (2025). Natural compounds as nephroprotective agents: therapeutic potential against drug-Induced kidney injury. J. Biochem. Mol. Toxicol. 39 (12), e70649. 10.1002/jbt.70649 41420444

[B153] Si S. Wu K. Zhang X. Zhang L. Wang W. Xu X. (2026). Research progress of nanoparticles in the diagnosis and treatment of renal ischemia-reperfusion injury. J. Nanobiotechnology 24 (1), 201. 10.1186/s12951-025-03986-8 41622179 PMC12951938

[B154] Song X. Wang W. Liu L. Zhao Z. Shen X. Zhou L. (2024). Poria cocos attenuated DSS-induced ulcerative colitis via NF-kappaB signaling pathway and regulating gut microbiota. Molecules 29 (9), 2154. 10.3390/molecules29092154 38731645 PMC11085930

[B155] Song S. Su W. Wang H. Hong X. Xiao Y. Zeng X. (2025). Advances in therapeutic strategies for acute kidney injury. Life Sci. 380, 123943. 10.1016/j.lfs.2025.123943 40902875

[B156] Su Y. Xu J. Chen S. Feng J. Li J. Lei Z. (2022). Astragaloside IV protects against ischemia/reperfusion (I/R)-induced kidney injury based on the Keap1-Nrf2/ARE signaling pathway. Transl. Androl. Urol. 11 (8), 1177–1188. 10.21037/tau-22-505 36092836 PMC9459544

[B157] Sun Y. Xun L. Jin G. Shi L. (2018). Salidroside protects renal tubular epithelial cells from hypoxia/reoxygenation injury in vitro. J. Pharmacol. Sci. 137 (2), 170–176. 10.1016/j.jphs.2018.05.011 29960844

[B158] Sun W. Li A. Wang Z. Sun X. Dong M. Qi F. (2020). Tetramethylpyrazine alleviates acute kidney injury by inhibiting NLRP3/HIF-1alpha and apoptosis. Mol. Med. Rep. 22 (4), 2655–2664. 10.3892/mmr.2020.11378 32945382 PMC7453617

[B234] Sun D. Cui S. Ma H. Zhu P. Li N. Zhang X. (2022). Salvianolate ameliorates renal tubular injury through the Keap1/Nrf2/ARE pathway in mouse kidney ischemia-reperfusion injury. J. Ethnopharmacol. 293, 115331. 10.1016/j.jep.2022.115331 35489662

[B159] Sun D. Wang L. Wu Y. Yu Y. Yao Y. Yang H. (2025). Lipid metabolism in ferroptosis: mechanistic insights and therapeutic potential. Front. Immunol. 16, 1545339. 10.3389/fimmu.2025.1545339 40134420 PMC11932849

[B160] Takhtfooladi M. A. Asghari A. Hoseinzadeh H. A. Mokhtari F. (2016). Effect of Otostegia persica extract on ischemia/reperfusion induced renal damage in diabetic rats. A biochemical study. Acta Cir. Bras. 31 (6), 417–421. 10.1590/S0102-865020160060000009 27355750

[B161] Tan R. J. Liu Y. (2024). Matrix metalloproteinases in kidney homeostasis and diseases: an update. Am. J. Physiol. Ren. Physiol. 327 (6), F967–F984. 10.1152/ajprenal.00179.2024 PMC11687849 39361724

[B162] Tang H. Huang L. Zhao D. Sun C. Song P. (2020). Interaction mechanism of flavonoids on bovine serum albumin: insights from molecular property-binding affinity relationship. Spectrochim. Acta A Mol. Biomol. Spectrosc. 239, 118519. 10.1016/j.saa.2020.118519 32480277

[B163] Tang S. Xie X. Wang M. Yang L. Wei W. (2022). Protective effects of asiaticoside on renal ischemia reperfusion injury *in vivo* and in vitro. Bioengineered 13 (4), 10235–10243. 10.1080/21655979.2022.2061302 35435108 PMC9161827

[B164] Tang Q. Li J. Wang Y. Sun Q. (2023). Identification and verification of hub genes associated with ferroptosis in ischemia and reperfusion injury during renal transplantation. Int. Immunopharmacol. 120, 110393. 10.1016/j.intimp.2023.110393 37279643

[B165] Tang Z. Feng Y. Nie W. Li C. (2023a). Xanthohumol attenuates renal ischemia/reperfusion injury by inhibiting ferroptosis. Exp. Ther. Med. 26 (6), 571. 10.3892/etm.2023.12269 37954118 PMC10632967

[B166] Tang Z. Wang Y. Liu Y. Li C. (2023b). Salidroside inhibits renal ischemia/reperfusion injury-induced ferroptosis by the PI3K/AKT signaling pathway. Exp. Ther. Med. 26 (5), 507. 10.3892/etm.2023.12206 37822587 PMC10562959

[B167] Tang W. Liu H. Li X. Deng S. (2026). The key role and research progress of endothelial cells in renal microcirculation. Front. Med. (Lausanne) 13, 1717495. 10.3389/fmed.2026.1717495 41716809 PMC12913175

[B168] Thomas K. Zondler L. Ludwig N. Kardell M. Luneburg C. Henke K. (2022). Glutamine prevents acute kidney injury by modulating oxidative stress and apoptosis in tubular epithelial cells. JCI Insight 7 (21), e163161. 10.1172/jci.insight.163161 36107633 PMC9675453

[B169] Tian R. Guo S. Chen S. Wu J. Long A. Cheng R. (2025). Natural products as Nrf2 modulators for ferroptosis inhibition in renal disease therapy: recent progress and future prospects. Phytomedicine 136, 156342. 10.1016/j.phymed.2024.156342 39742572

[B170] Tong W. Mu X. Xu H. Yin X. (2026). Natural products as modulators of ferroptosis: therapeutic implications and molecular mechanisms in disease treatment. J. Nutr. Biochem. 151, 110259. 10.1016/j.jnutbio.2026.110259 41490559

[B171] Topkaraoglu S. Sapmaz T. Sevgin K. Tekayev M. Kuras S. Pence H. H. (2026). Combined melatonin and curcumin treatment ameliorates renal ischemia-reperfusion injury via reducing oxidative stress, inflammation, and apoptosis in rats. Biotech. Histochem. 101 (4), 215–225. 10.1080/10520295.2026.2642383 41869692

[B172] Toprak T. Sekerci C. A. Aydin H. R. Ramazanoglu M. A. Arslan F. D. Basok B. I. (2020). Protective effect of chlorogenic acid on renal ischemia/reperfusion injury in rats. Arch. Ital. Urol. Androl. 92 (2), 153–157. 10.4081/aiua.2020.2.153 32597123

[B173] Tu H. Tang L. J. Luo X. J. Ai K. L. Peng J. (2021). Insights into the novel function of system Xc- in regulated cell death. Eur. Rev. Med. Pharmacol. Sci. 25 (3), 1650–1662. 10.26355/eurrev_202102_24876 33629335

[B174] Wang X. Huang F. (2025). Berberine attenuates pyroptosis in renal tubular epithelial cells by activating the FOXO3a/ARC pathway. J. Appl. Toxicol. 45 (11), 2289–2296. 10.1002/jat.4837 40582714

[B175] Wang C. Hao Z. Zhou J. Zhang L. Sun Y. Liang C. (2017). Rutaecarpine alleviates renal ischemia reperfusion injury in rats by suppressing the JNK/p38 MAPK signaling pathway and interfering with the oxidative stress response. Mol. Med. Rep. 16 (1), 922–928. 10.3892/mmr.2017.6631 28560443

[B176] Wang M. Weng X. Chen H. Chen Z. Liu X. (2020). Resveratrol inhibits TNF-alpha-induced inflammation to protect against renal ischemia/reperfusion injury in diabetic rats. Acta Cir. Bras. 35 (5), e202000506. 10.1590/s0102-865020200050000006 32638845 PMC7341989

[B177] Wang Y. Han K. Li Z. Tang X. Wang C. Zhao Y. (2022a). Protective effect of hydroxysafflor yellow A on renal ischemia--reperfusion injury by targeting the Akt-Nrf2 axis in mice. Exp. Ther. Med. 24 (6), 741. 10.3892/etm.2022.11677 36478883 PMC9716340

[B178] Wang Y. Liu Q. Cai J. Wu P. Wang D. Shi Y. (2022b). Emodin prevents renal ischemia-reperfusion injury via suppression of CAMKII/DRP1-mediated mitochondrial fission. Eur. J. Pharmacol. 916, 174603. 10.1016/j.ejphar.2021.174603 34793771

[B179] Wang J. Yu Y. Zhang H. Li L. Wang J. Su S. (2024). Gypenoside XVII attenuates renal ischemia-reperfusion injury by inhibiting endoplasmic reticulum stress and NLRP3 inflammasome-triggered pyroptosis. Eur. J. Pharmacol. 962, 176187. 10.1016/j.ejphar.2023.176187 37984729

[B180] Wang M. Chen Z. Tang Z. Tang S. (2024a). Natural products derived from traditional Chinese medicines targeting ER stress for the treatment of kidney diseases. Ren. Fail. 46 (2), 2396446. 10.1080/0886022X.2024.2396446 39192602 PMC11360642

[B181] Wang M. Li Q. Wang S. Zuo L. Hai Y. Yuan S. (2024b). Astragaloside IV protects renal tubular epithelial cells against oxidative stress-induced injury by upregulating CPT1A-mediated HSD17B10 lysine succinylation in diabetic kidney disease. Phytother. Res. 38 (9), 4519–4540. 10.1002/ptr.8298 39038923

[B182] Wang J. Zheng Q. Chen Z. Liu X. Wan S. Wang L. (2025). Puerarin alleviates renal ischemia/reperfusion injury by inhibiting apoptosis and endoplasmic reticulum stress via Nrf2/HO-1 pathway. Iran. J. Basic Med. Sci. 28 (2), 187–193. 10.22038/ijbms.2024.80438.17412 39850122 PMC11756738

[B183] Wang N. Xue X. Zhang Z. Gao M. Yin L. Xu L. (2025). Curcumin-loaded nanoparticles for renal ischemia-reperfusion injuries: Triple-play of redox homeostasis accommodation, lipid metabolism regulation, and nuclear magnetic tracing. Mater. Today Bio. 33, 101986. 10.1016/j.mtbio.2025.101986 40600177 PMC12212281

[B184] Wang X. Chang X. Yang D. Zhang L. Guo Z. Sun X. (2025). Tetramethylpyrazine alleviates acute kidney injury by activating the Wnt/beta-catenin pathway independent of DKK1. Exp. Ther. Med. 30 (5), 208. 10.3892/etm.2025.12958 40927640 PMC12416129

[B185] Wang Y. Wang N. Qu L. Jiao Y. Xiao Z. (2025). Nanomedicine for acute kidney injury: precision delivery strategies, therapeutic breakthroughs, challenges, and future perspectives. Int. J. Nanomedicine 20, 14015–14031. 10.2147/IJN.S550080 41321901 PMC12658368

[B186] Wei J. P. Zhao B. Jiang Z. J. Wang P. Y. Xu Y. Ding N. (2025). Luteolin mitigates renal ischemia-reperfusion injury via anti-inflammatory, anti-apoptotic, and Nrf2/HO-1-mediated antioxidant effects. Eur. J. Pharmacol. 999, 177676. 10.1016/j.ejphar.2025.177676 40306537

[B187] Xia K. Jin Z. Qiu Q. Zhou Y. Lu Y. Qiu T. (2024). Ligustilide alleviates oxidative stress during renal ischemia-reperfusion injury through maintaining Sirt3-dependent mitochondrial homeostasis. Phytomedicine 134, 155975. 10.1016/j.phymed.2024.155975 39216302

[B188] Xiao Z. Vijayalakshmi A. (2022). Protective effect of piperlongumine on inflammation and oxidative stress against ischemia-reperfusion injury in animal kidney. Bratisl. Lek. Listy 123 (12), 878–884. 10.4149/BLL_2022_140 36342874

[B189] Xiao Y. Shan X. Wang H. Hong B. Ge Z. Ma J. (2022). Spectrum-effect relationship between HPLC fingerprint and antioxidant of “San-Bai Decoction” extracts. J. Chromatogr. B 1208, 123380. 10.1016/j.jchromb.2022.123380 35908440

[B190] Xing W. Wen C. Wang D. Shao H. Liu C. He C. (2022). Cardiorenal protective effect of costunolide against doxorubicin-induced toxicity in rats by modulating oxidative stress, inflammation and apoptosis. Molecules 27 (7), 2122. 10.3390/molecules27072122 35408518 PMC9000510

[B191] Xu Y. M. Ding G. H. Huang J. Xiong Y. (2016). Tanshinone IIA pretreatment attenuates ischemia/reperfusion-induced renal injury. Exp. Ther. Med. 12 (4), 2741–2746. 10.3892/etm.2016.3674 27698779 PMC5038172

[B192] Xu Y. Wang W. Jin K. Zhu Q. Lin H. Xie M. (2017). Perillyl alcohol protects human renal tubular epithelial cells from hypoxia/reoxygenation injury via inhibition of ROS, endoplasmic reticulum stress and activation of PI3K/Akt/eNOS pathway. Biomed. Pharmacother. 95, 662–669. 10.1016/j.biopha.2017.08.129 28886525

[B193] Xu C. H. Deng Y. Man J. W. Wang H. B. Che T. J. Ding L. Y. (2024). Unveiling the renoprotective mechanisms of schisandrin B in ischemia-reperfusion injury through transcriptomic and pharmacological analysis. Drug Des. devel. Ther. 18, 4241–4256. 10.2147/Dddt.S489458 39323973 PMC11423835

[B194] Xu C. Wang H. Wang H. Man J. Deng Y. Li Y. (2025). Schisandrin B regulates mitochondrial dynamics via AKT1 activation and mitochondrial targeting to ameliorate renal ischemia-reperfusion injury. Phytomedicine 141, 156672. 10.1016/j.phymed.2025.156672 40220406

[B195] Xu X. Zeng T. Chen S. Tian N. Zhang C. Chen Y. (2025). Acute kidney injury: pathogenesis and therapeutic interventions. Mol. Biomed. 6 (1), 61. 10.1186/s43556-025-00293-4 40911105 PMC12413393

[B196] Xue L. Jiang S. Wan X. Y. (2024). Protective effects of sesamol on renal ischemia-reperfusion injury via regulation of nuclear factor erythroid 2-related factor 2 pathway. Transpl. Proc. 56 (2), 290–296. 10.1016/j.transproceed.2023.12.009 38350822

[B197] Yan R. Zhang Y. Li Z. Wang H. Wang K. Man J. (2025). Core signaling pathways in renal ischemia-reperfusion injury: network regulation and therapeutic prospects. Cell Signal 136, 112185. 10.1016/j.cellsig.2025.112185 41130330

[B198] Yang Q. Zang H. M. Xing T. Zhang S. F. Li C. Zhang Y. (2021). Gypenoside XLIX protects against acute kidney injury by suppressing IGFBP7/IGF1R-mediated programmed cell death and inflammation. Phytomedicine 85, 153541. 10.1016/j.phymed.2021.153541 33773190

[B199] Yang D. Tang M. Zhang M. Ren H. Li X. Zhang Z. (2023). Downregulation of G protein-coupled receptor kinase 4 protects against kidney ischemia-reperfusion injury. Kidney Int. 103 (4), 719–734. 10.1016/j.kint.2022.12.023 36669643 PMC12621542

[B200] Yao M. Qin S. Xiong J. Xin W. Guan X. Gong S. (2022). Oroxylin A ameliorates AKI-to-CKD transition through maintaining PPARalpha-BNIP3 signaling-mediated mitochondrial homeostasis. Front. Pharmacol. 13, 935937. 10.3389/fphar.2022.935937 36081929 PMC9445212

[B201] Yarijani Z. M. Pourmotabbed A. Pourmotabbed T. Najafi H. (2017). Crocin has anti-inflammatory and protective effects in ischemia-reperfusion induced renal injuries. Iran. J. Basic Med. Sci. 20 (7), 753–759. 10.22038/IJBMS.2017.9005 28852439 PMC5569594

[B202] Ye Q. Zhu Y. I. Ye S. Liu H. She X. Niu Y. (2016). Gypenoside attenuates renal ischemia/reperfusion injury in mice by inhibition of ERK signaling. Exp. Ther. Med. 11 (4), 1499–1505. 10.3892/etm.2016.3034 27073472 PMC4812469

[B203] Ye Q. Chen H. Ma H. Xiang X. Hu S. Xia C. (2021). Xiaoyu Xiezhuo Drink protects against ischemia-reperfusion acute kidney injury in aged mice through inhibiting the TGF-beta1/Smad3 and HIF1 signaling pathways. Biomed. Res. Int. 2021, 9963732. 10.1155/2021/9963732 34545331 PMC8449228

[B204] Yin S. Liu J. Zhao X. Dong H. Cao Y. Zhang S. (2023). Chitosan oligosaccharide attenuates acute kidney injury and renal interstitial fibrosis induced by ischemia-reperfusion. Ren. Fail. 45 (1), 2238831. 10.1080/0886022X.2023.2238831 37482748 PMC10367574

[B205] Younis N. S. (2023). Myrrh essential oil mitigates renal ischemia/reperfusion-induced injury. Curr. Issues Mol. Biol. 45 (2), 1183–1196. 10.3390/cimb45020078 36826023 PMC9955815

[B206] Younis N. S. Ghanim A. M. H. (2022). The protective role of celastrol in renal ischemia-reperfusion injury by activating Nrf2/HO-1, PI3K/AKT signaling pathways, modulating NF-kappab signaling pathways, and inhibiting ERK phosphorylation. Cell Biochem. Biophys. 80 (1), 191–202. 10.1007/s12013-022-01064-6 35157199 PMC8881435

[B207] Yousefi-Manesh H. Hemmati S. Shirooie S. Nabavi S. M. Bonakdar A. T. Fayaznia R. (2019). Protective effects of hydroalcoholic extracts from an ancient apple variety 'Mela Rosa dei Monti Sibillini' against renal ischemia/reperfusion injury in rats. Food Funct. 10 (11), 7544–7552. 10.1039/c9fo01635j 31686074

[B208] Yue Q. Song Y. Liu Z. Zhang L. Yang L. Li J. (2022). Receptor for advanced glycation end products (RAGE): a pivotal hub in immune diseases. Molecules 27 (15), 4922. 10.3390/molecules27154922 35956875 PMC9370360

[B209] Zeng Y. Liu F. Wang J. Shao B. He T. Xiang Z. (2025). Fisetin micelles precisely exhibit a radiosensitization effect by inhibiting PDGFR β/STAT1/STAT3/Bcl-2 signaling pathway in tumor. Chin. Chem. Lett. 36 (2), 109734. 10.1016/j.cclet.2024.109734

[B210] Zhang Z. Qi D. Wang X. Gao Z. Li P. Liu W. (2018). Protective effect of Salvianolic acid A on ischaemia-reperfusion acute kidney injury in rats through protecting against peritubular capillary endothelium damages. Phytother. Res. 32 (1), 103–114. 10.1002/ptr.5954 29071768

[B211] Zhang S. Xu X. Huang Y. Sun S. Jin C. Ji H. (2021). Anisodamine ameliorates ischemia/reperfusion-induced renal injury in rats through activation of the extracellular signal-regulated kinase (ERK) pathway and anti-apoptotic effect. Pharmazie 76 (5), 220–224. 10.1691/ph.2021.1302 33964996

[B229] Zhang Y. Liu M. Zhang Y. Tian M. Chen P. Lan Y. (2022). Urolithin A alleviates acute kidney injury induced by renal ischemia reperfusion through the p62-Keap1-Nrf2 signaling pathway. Phytother. Res. 36, 984–995. 10.1002/ptr.7370 35040204

[B212] Zhang M. Liu Q. Meng H. Duan H. Liu X. Wu J. (2024). Ischemia-reperfusion injury: molecular mechanisms and therapeutic targets. Signal Transduct. Target Ther. 9 (1), 12. 10.1038/s41392-023-01688-x 38185705 PMC10772178

[B213] Zhao J. Y. Wu Y. B. (2020). Huaier extract attenuates acute kidney injury to chronic kidney disease transition by inhibiting endoplasmic reticulum stress and apoptosis *via* miR-1271 Upregulation. Biomed. Res. Int. 2020, 9029868. 10.1155/2020/9029868 33457422 PMC7787756

[B214] Zhao L. Xu L. Tao X. Han X. Yin L. Qi Y. (2016). Protective effect of the total flavonoids from Rosa laevigata michx fruit on renal ischemia-reperfusion injury through suppression of oxidative stress and inflammation. Molecules 21 (7), 952. 10.3390/molecules21070952 27455216 PMC6272996

[B215] Zhao H. H. Han Q. X. Ding X. N. Yan J. Y. Li Q. Zhang D. (2020). Critical hubs of renal ischemia-reperfusion injury: endoplasmic reticulum-mitochondria tethering complexes. Chin. Med. J. 133 (21), 2599–2609. 10.1097/CM9.0000000000001091 32960842 PMC7722596

[B216] Zhao F. Zhu J. Zhang M. Luo Y. Li Y. Shi L. (2023). OGG1 aggravates renal ischemia-reperfusion injury by repressing PINK1-mediated mitophagy. Cell Prolif. 56 (8), e13418. 10.1111/cpr.13418 36788635 PMC10392062

[B217] Zheng Y. Zhang Y. Zheng Y. Zhang N. (2018). Carnosol protects against renal ischemia-reperfusion injury in rats. Exp. Anim. 67 (4), 545–553. 10.1538/expanim.18-0067 30068825 PMC6219873

[B218] Zheng Y. Zhang N. Bai F. (2022). Gastrodin pretreatment alleviates renal ischemia-reperfusion injury. Urol. Int. 106 (6), 630–637. 10.1159/000520531 35051947

[B219] Zhong D. Wang H. Liu M. Li X. Huang M. Zhou H. (2015). Ganoderma lucidum polysaccharide peptide prevents renal ischemia reperfusion injury *via* counteracting oxidative stress. Sci. Rep. 5, 16910. 10.1038/srep16910 26603550 PMC4658483

[B220] Zhou Q. Gong X. Kuang G. Jiang R. Xie T. Tie H. (2018). Ferulic acid protected from kidney ischemia reperfusion injury in mice: possible mechanism through increasing adenosine generation via HIF-1alpha. Inflammation 41 (6), 2068–2078. 10.1007/s10753-018-0850-3 30143933

[B221] Zhou Y. Du D. Liu S. Zhao M. Yuan Y. Li L. (2018). Polyacetylene glycoside attenuates ischemic kidney injury by co-inhibiting inflammation, mitochondria dysfunction and lipotoxicity. Life Sci. 204, 55–64. 10.1016/j.lfs.2018.05.009 29733848

[B226] Zhou Q. Li J. Xiang Z. Zou H. Shao X. (2022). Amelioration of renal injury by resveratrol in a rat renal transplantation model via activation of the SIRT1/NF-κB signaling pathway. BioMed Res. Int. 2022, 7140961. 10.1155/2022/7140961 35386302 PMC8979694

[B222] Zhou P. Wang Z. Chen C. Famurewa A. C. Olatunji O. J. (2023). Naringin ameliorates 5-fluorouracil elicited neurotoxicity by curtailing oxidative stress and iNOS/NF-κB/caspase-3 pathway. Open Chem. 21 (1), 20230126. 10.1515/chem-2023-0126

[B223] Zhou J. Feng Y. Zhou W. Zhang M. Liu F. Mao J. (2025). Ultrasound-assisted metabolite detection in different extraction processes of Bletilla striata and bitter metabolite detection. Ultrason. Sonochem. 114, 107266. 10.1016/j.ultsonch.2025.107266 39952165 PMC12013125

[B224] Zhu Y. L. Huang J. Chen X. Y. Xie J. Yang Q. Wang J. F. (2022). Senkyunolide I alleviates renal ischemia-reperfusion injury by inhibiting oxidative stress, endoplasmic reticulum stress and apoptosis. Int. Immunopharmacol. 102, 108393. 10.1016/j.intimp.2021.108393 34857480

[B225] Zhu J. Shen H. Li G. Chen L. Kang P. Guo Y. (2024). Theaflavin pretreatment ameliorates renal ischemia/reperfusion injury by attenuating apoptosis and oxidative stress *in vivo* and *in vitro* . Biomed. Pharmacother. 171, 116114. 10.1016/j.biopha.2023.116114 38171247

