# Supplementary material for: Plant metabolites: potential treatments for ischemic acute kidney injury
Source: Front Pharmacol. 2026 Aug 14;17:1891651. doi: 10.3389/fphar.2026.1891651 (PMC13524399; doi:10.3389/fphar.2026.1891651)
Supplement: Supplementary file 1 [file Table1.docx]

**Table S1.** Protective effect of plant metabolites against ischemic AKI

| **Metabolites** | **Structure** | **Model** **(Animal species, sex,** **I/R duration)/ Cells** | **Treatment dosage and duration** | **Effect** | **Mechanism** | **Ref** |
| --- | --- | --- | --- | --- | --- | --- |
| **Phenylpropanoids** |  |  |  |  |  |  |
| Anethole |  | *In vivo*: Wistar rats (male), BIRI (45 min/24 h) | 125/250 mg/kg/d, [p.o.], 14 days before BIRI | ⭡Renal function; ↓Oxidative stress and apoptosis | ↓HMGB1/TLR/MyD88/NF-κB | (Mohamed et al., 2022b) |
| Eugenol |  | *In vivo*: C57BL/6 mice (male), BIRI (45 min/24 h);  *In vitro*: TCMK-1 cells, H/R (8 h/12 h) | 1/10/100/1000 mg/kg/d, [p.o.], 7 days before BIRI;  1–1000 μM in TCMK-1 cells, 100 μM selected | ⭣Renal damage; oxidative stress and apoptosis | ⭡Sestrin2 | (Liu et al., 2025a) |
| Ferulic Acid |  | *In vivo*: C57BL/6 mice (male), BIRI (35 min/24 h) | 10/30/100 mg/kg, [p.o.], 3 times within 24 h before BIRI | ↓Renal injury; ↓Inflammation and apoptosis; ⭡Adenosine generation | ⭡CD39/CD73 via HIF-1α | (Zhou et al., 2018) |
| Ligustilide |  | *In vivo*: C57BL/6 mice (male), UIRI/UNX (30 min/24 h);  *In vitro*: HK-2 cells, H/R (1 h/12 h) | 10/20 mg/kg/d, [i.p.], 3 days before UIRI/UNX;  10/20 μM, 24 h before H/R | ⭡Mitochondrial homeostasis; ↓Oxidative stress and apoptosis | ⭡Sirt3 | (Xia et al., 2024) |
| Methyl eugenol |  | *In vivo*: C57BL/6J mice (male), UIRI/UNX (35 min/24 h);  *In vitro*: HK-2 cells, H/R (24 h/2 h) | 5/10/20/40 mg/kg/d, [i.p.], 5 days before UIRI/UNX;  40 μM | ↓Renal injury; ↓ Oxidative stress and apoptosis | ⭡AMPK/GSK3β, ⭡(nuclear) Nrf2 | (Kuang et al., 2023) |

(*continued on next page*)

**Table 1.** (*continued*)

| **Metabolites** | **Structure** | **Model** **(Animal species, sex,** **I/R duration)/ Cells** | **Treatment dosage and duration** | **Effect** | **Mechanism** | **Ref** |
| --- | --- | --- | --- | --- | --- | --- |
| Nodakenin |  | *In vivo*: C57BL/6 mice (male), BIRI (35 min/3 d);  *In vitro*: PTC cells, (H/12 h) | 20 mg/kg/d, [p.o.], before BIRI and until day 3;  5/10/20 μM, 12 h before hypoxia | ⭣Renal dysfunction and tubular necrosis, ⭣Inflammation | ⭣ROS/NLRP3/caspase-1 and NF-κB | (Liao et al., 2021) |
| Salidroside |  | *In vitro*: HK-2 cells, H/R (24 h/12 h) | 6.25/12.5/25 μM, 2 h before H/R | ⭡Cell viability; ⭣Oxidative stress, inflammation, and apoptosis | ⭣TLR4/NF-κB | (Sun et al., 2018) |
|  |  | *In vivo*: SD rats (male), BIRI (45 min/24 h);  *In vitro*: NRK cells, H/R (8 h/12 h) | 1/10/100 mg/kg/d, [p.o.], 7 days before BIRI;  1–1000 μM at reoxygenation onset, 100 μM selected | ⭡Renal function and cell viability; ⭣Renal injury, oxidative stress, and ferroptosis | ⭡PI3K/AKT | (Tang et al., 2023b) |
| Schisantherin A |  | *In vitro*: HK-2 cells, H/R (4 h/2 h) | 5/10/20 μM, 24 h before H/R; 20 μM used for mechanism study | ⭡Cell viability; ⭣Oxidative stress, inflammation, and apoptosis | ⭡PI3K/Akt | (Gong and Wang, 2018) |
| Schisandrin B |  | *In vivo*: BALB/c mice (male), BIRI (45 min/24 h);  *In vitro*: HK-2 cells, H/R (12 h/2 h) | 20/40 mg/kg/d, [p.o.], 7 days before BIRI;  20 μM, 12 h before H/R | ⭣Renal injury, oxidative stress, inflammation, and apoptosis | ↑PI3K/AKT | (Xu et al., 2024) |
|  |  | *In vivo*: BALB/c mice (male), BIRI (45 min/24 h);  *In vitro*: HK-2 cells, H/R (12 h/2 h) | 20/40 mg/kg/d, [p.o.], 7 days before BIRI;  5/10/20 μM, 12 h before H/R; 20 μM selected | ⭣Renal injury, oxidative stress, apoptosis | ↑AKT1-mediated mitochondrial dynamics | (Xu et al., 2025) |

(*continued on next page*)

**Table 1.** (*continued*)

| **Metabolites** | **Structure** | **Model** **(Animal species, sex,** **I/R duration)/ Cells** | **Treatment dosage and duration** | **Effect** | **Mechanism** | **Ref** |
| --- | --- | --- | --- | --- | --- | --- |
| Sesamol |  | *In vivo*: C57BL/6J mice (male), UIRI/UNX (30 min/24 h) | 30 mg/kg/d, 6 days before UIRI/UNX | ↑Renal function; ↓ Inflammation and oxidative stress | ↑Nrf2/HO-1/NQO1 | (Xue et al., 2024) |
| Sesamin |  | *In vivo*: C57BL/6 mice (male), UIRI/UNX (30 min/24 h) | 100 mg/kg, [p.o.], every 8 h for 3 times within 24 h before UIRI/UNX | ↓Kidney damage and inflammation | ↑CD39-Adenosine-A2AR | (Li et al., 2016) |
| Senkyunolide I |  | *In vivo*: C57BL/6J mice (male), BIRI (40 min/24 h);  *In vitro*: HK-2 cells, H₂O₂-induced oxidative stress model | 36 mg/kg, [i.p.], during and 30 min after BIRI;  100/200/300 μg/mL before H₂O₂ | ⭣Renal injury, inflammation, oxidative stress, and apoptosis | ↑Nrf2/HO-1 | (Zhu et al., 2022) |
| Syringic Acid |  | *In vivo*: Wistar rats (male), BIRI (30 min/1 h) | 10 mg/kg, [i.p.], 0, 12 and 24 h before BIRI; last dose 30 min before BIRI | ↓Kidney damage, oxidative stress, and apoptosis | NR | (Sancak et al., 2016) |
| Trans-Cinnamaldehyde |  | *In vivo*: C57BL/6J mice (male), BIRI (40 min/24 h);  *In vitro*: HK-2 cells, H/R (6 h/12 h) or treated with CoCl₂ (600 μM, 12 h) | 40 mg/kg/d, [i.p.], 3 days before BIRI;  10/20 μM, 2 h before H/R or CoCl₂ injury | ⭣Renal injury and inflammation | ⭣JNK/p38 MAPK | (Chen et al., 2023) |
| **Polyphenols（non-flavonoids）** | |  |  |  |  |  |
| Chlorogenic Acid |  | *In vivo*: SD rats (male), UIRI (45 min/4 h) | 20 mg/kg, [i.p.], 10 min before reperfusion | ↓Renal histology, ↓oxidative stress | NR | (Toprak et al., 2020) |

(*continued on next page*)

**Table 1.** (*continued*)

| **Metabolites** | **Structure** | **Model** **(Animal species, sex,** **I/R duration)/ Cells** | **Treatment dosage and duration** | **Effect** | **Mechanism** | **Ref** |
| --- | --- | --- | --- | --- | --- | --- |
| Curcumin |  | *In vivo*: SD rats (male), BIRI (45 min/24 h) | 15/30/60 mg/kg; [i.v.], 5 days | ⭣Renal injury, oxidative stress, inflammation, and apoptosis | NR | (Cui et al., 2021) |
|  |  | *In vivo*: BALB/c mice (male), BIRI (30 min/24 h);  *In vitro*: EpiCM-a cells, H/R (3 h/24 h) | 100 mg/kg, [i.p.], 0.5 h before BIRI;  25 μM at the beginning of H/R | ⭣Renal injury and apoptosis | ↑APPL1; ⭣ Akt phosphorylation | (Fan et al., 2017) |
|  |  | *In vivo*: Wistar albino rats, UIRI/UNX (45 min/24 h); | 100 mg/kg, [i.p.], 10 min before reperfusion; | ↓Renal injury, oxidative stress, inflammation, and apoptosis | ↓SEMA3A | (Kar et al., 2020) |
|  |  | *In vivo*: Wistar albino rats (female), BIRI (40 min/24 h); | 30/60 mg/kg, [p.o.], 1 h before BIRI; | ↓Renal histological injury and oxidative stress | NR | (Kaur et al., 2016) |
|  |  | *In vivo*: C57BL/6 mice (male), UIRI/UNX (45 min/24h)  *In vitro*: HK-2 cells, H/R (24 h/2 h) | 50 mg/kg, [i.p.], after/around UIRI/UNX  5 μM during H/R | ↑Mitochondrial function; ↓ oxidative stress, inflammation, apoptosis | ↑NRF2, ↓NF-κB | (Li et al., 2021a) |
|  |  | *In vivo*: SD rats (male), UIRI by (45 min/24 h);  *In vitro*: HK-2 cells, | 100 mg/kg, tail vein injection, 2 h before renal I/R; | ↓Renal tubular injury and apoptosis | ↓iNOS/NO/cGMP/PKG | (Liu et al., 2017a) |
|  |  | *In vivo*: Wistar rats (sex NR), BIRI (45 min/6 h); | 5/10 mg/kg/d, [p.o.], 2 weeks before BIRI; | ↓Renal injury, inflammation, oxidative stress, and apoptosis | NR | (Liu et al., 2016) |
|  |  | *In vivo*: Wistar rats (male), BIRI (30 min; renal assessment on day 28 after day-26 I/R); | 30 mg/kg/d, [p.o.], days 17–27; BIRI performed on day 26; | ↑Thiol antioxidant reserve | NR | (Machado et al., 2022) |
|  |  | *In vivo*: SD rats (male), UIRI/UNX (45 min/24 h); | 60 mg/kg, [i.p.], 45 min before UIRI/UNX; | ↓Renal pathological injury and inflammation | ↑JAK2/STAT3; ↓ NF-κB | (Zhang et al., 2018a) |

(*continued on next page*)

**Table 1.** (*continued*)

| **Metabolites** | **Structure** | **Model** **(Animal species, sex,** **I/R duration)/ Cells** | **Treatment dosage and duration** | **Effect** | **Mechanism** | **Ref** |
| --- | --- | --- | --- | --- | --- | --- |
| Ellagic Acid |  | *In vivo*: SD rats (sex NR), UIRI (45 min/1 h);  *In vitro*: NRK-52E cells, H/R (24 h/12 h) | 0/100/150 mg/kg, [p.o.], 30 min before UIRI;  5/10/20 μM, 24 h before H/R | ↑Renal function, ↓oxidative stress, inflammation, and apoptosis | ↓NOX4/JAK/STAT | (Liu et al., 2020b) |
| Gallic Acid |  | *In vivo*: SD rats (male), BIRI (45 min/24 h) | 100 mg/kg/d, [i.p.], 15 days before BIRI; | ↓Renal oxidative stress and functional impairment | NR | (Ahmadvand et al., 2019) |
| Oleuropein |  | *In vivo*: Wistar rats (male), BIRI (60 min/120 min) | 10, 50, 100 mg/kg, [p.o.], 48 h, 24 h, and 30 min before BIRI; 50 mg/kg used for mechanism study | ↑Renal function, ↓oxidative stress, inflammation, and apoptosis | ↑AMPK/eNOS, ↓COX-2 | (Nasrallah et al., 2020) |
| Pterostilbene |  | *In vivo*: SD rats (male), IRI (duration not reported) | 0, 20, 30 mg/kg/d, [p.o.], from 24 h after IRI surgery for 2 weeks | ↓Renal injury, ↓oxidative stress, and inflammation | ↓TLR4/NF-κB | (Gao et al., 2018) |
| Resveratrol |  | *In vivo*: SD rats (male), IRI (30 min/2 h) | 30 mg/kg, [i.p.], 30 min before IRI | ↓Renal injury, oxidative stress, inflammation, and apoptosis | NR | (Alaasam et al., 2024) |
|  |  | *In vivo*: Wistar rats (male), UIRI (60 min/60 min) | 60 mg/kg/day, [p.o., drinking water], 3 weeks before IRI | ↓lipid peroxidation; histological injury | NR | (Baltaci et al., 2019) |
|  |  | *In vivo*: SD rats (male), UIRI (60 min/60 min) | 20 mg/kg/d, [p.o.], 14 days before IRI | ↓Apoptosis | ↑SIRT1 | (Erkasap et al., 2017) |
|  |  | *In vivo*: SD rats (male), UIRI/UNX (60 min/24 h);  *In vitro*: NRK-52E cells, H₂O₂ (4 h) | 0.23 μg/kg, [p.o.], 30 min before IRI;  100 μM, 1 h before H₂O₂ | ↓Renal injury, inflammation, oxidative stress, and apoptosis | ↑Nrf2/HO-1; ↓ TLR4/NF-κB | (Li et al., 2018) |

(*continued on next page*)

**Table 1.** (*continued*)

| **Metabolites** | **Structure** | **Model** **(Animal species, sex,** **I/R duration)/ Cells** | **Treatment dosage and duration** | **Effect** | **Mechanism** | **Ref** |
| --- | --- | --- | --- | --- | --- | --- |
|  |  | *In vivo*: Wistar rats (male), IRI (30 min/2 h) | 20 mg/kg/d, [p.o.], 24 h after IRI, daily for 10 days | ↓Renal injury, inflammation, oxidative stress, and apoptosis | NR | (Martinez-Rojas et al., 2024) |
|  |  | *In vivo*: SD rats (male), UIRI (45 min/24 h) | 10 mg/kg/d, [p.o.], 14 days before IRI | ↓Renal injury, oxidative stress, inflammation, and apoptosis | ↓TNF-α/NF-κB | (Wang et al., 2020a) |
| Rosmarinic Acid |  | *In vivo*: SD rats (male), BIRI (45 min/24 h) | 100 mg/kg, [i.p.], 30 min before BIRI | ↓Renal injury, inflammation, and oxidative stress | ↓TLR4 and NF-κB | (Firouzeh et al., 2025) |
| Salvianolic Acid A |  | *In vivo*: SD rats (male), BIRI (60 min/24 h) | 100 mg/kg, [i.p.], 30 min before BIRI | ↓Renal injury, protects the capillary endothelium | ↓ Platelet activation, ↑ VEGFA, and Klotho | (Zhang et al., 2018b) |
| Salvianolic Acid B |  | *In vivo*: BALB/c mice (male), UIRI/UNX (45 min/24 h);  *In vitro*: HK-2 cells H/R (6 h/1 h) | 50, 100, 200 mg/kg, [p.o.], daily for 1 week before UIRI/UNX  1, 5, 10, 20, 40, 80 μM screened; 80 μM for mechanism study, 24 h before H/R | ↓Renal injury and pyroptosis | ↑ Nrf2/NLRP3 | (Pang et al., 2020) |
| Tannic Acid |  | *In vivo*: SD rats (male), BIRI (60 min/3 h and 7 d);  *In vitro*: HAEC H/R (6 h/4 or 18 h) | 50 mg/kg, [i.p.], 30 min before BIRI  7.3 μM during H/R | ↑Renal function; ↓ROS generation and lipid oxidation; ↑ endothelial cell repair | ↑Nrf2 | (Alechinsky et al., 2020) |

(*continued on next page*)

**Table 1.** (*continued*)

| **Metabolites** | **Structure** | **Model** **(Animal species, sex,** **I/R duration)/ Cells** | **Treatment dosage and duration** | **Effect** | **Mechanism** | **Ref** |
| --- | --- | --- | --- | --- | --- | --- |
| Theaflavin |  | *In vivo*: C57BL/6J mice (male), BIRI (45 min/24 h)  *In vitro*: TCMK-1 cells, H/R (48 h/6 h) | 20, 50, 80 mg/kg/d, [p.o.], 3 days before BIRI  0, 2, 6, 10 μM, 2 h before H/R; 6 μM for mechanism study | ↓Renal injury, oxidative stress, apoptosis, and mitochondrial dysfunction | ↑Nrf2/HO-1/NQO1 | (Li et al., 2021b) |
|  |  | *In vivo*: C57BL/6J mice (male), BIRI (45 min/24 h)  *In vitro*: TCMK-1 cells, H/R (48 h/6 h) | 20, 50, 80 mg/kg/d, [p.o.], 3 days before BIRI  0, 2, 6, 10 μM, 2 h before H/R; 6 μM used for mechanism study | ↓Renal injury, oxidative stress, and apoptosis | ↑p53/GPX-1 | (Zhu et al., 2024) |
| Urolithin A |  | *In vivo*: C57BL/6 mice (male), BIRI (25 min/24 h);  *In vitro*: HK-2 cells H/R (24 h/12 h) | 20, 50, 100 mg/kg/d, [p.o.], 7 days before BIRI; 50 mg/kg/d selected as optimal dose | ↓Renal injury; ↓ apoptosis, oxidative stress, ↑autophagy | ↑p62-Keap1-Nrf2 | (Zhang et al., 2022c) |
| **Polyphenols（flavonoids）** | |  |  |  |  |  |
| Apigenin |  | *In vivo*: SD rats (male), BIRI (45 min/24 h);  *In vitro*: NRK-52E cells, H/R (3 h/24 h) | 4 mg/kg, [i.p.], 24 h before BIRI;  1 μM, 24 h before H/R | ↓Renal apoptosis | ↑JAK2/STAT3 | (Liu et al., 2017c) |
|  |  | *In vivo*: SD rats (male), UIRI/UNX (45 min/24 h);  *In vitro*: NRK-52E cells, H/R (3 h/24 h) | 2, 10, 50 mg/kg, [i.v.], 10 min before UIRI/UNX;  10, 100, 1000 nM, 24 h before H/R | ↓Renal apoptosis | ↑Bcl-2, ↓Fas/FasL | (Liu et al., 2017b) |
|  |  | *In vivo*: SD rats (male), UIRI/UNX (45 min/24 h);  *In vitro*: HK-2 cells, H/R (24 h/3h) | 20 mg/kg, [i.p.], 60 min before UIRI/UNX  20 μM, 60 min before H/R | ↓Renal tubular apoptosis | ↑PI3K/Akt | (Wang et al., 2018) |

(*continued on next page*)

**Table 1.** (*continued*)

| **Metabolites** | **Structure** | **Model** **(Animal species, sex,** **I/R duration)/ Cells** | **Treatment dosage and duration** | **Effect** | **Mechanism** | **Ref** |
| --- | --- | --- | --- | --- | --- | --- |
| Astilbin |  | *In vivo*: C57BL/6J mice (male), UIRI/UNX (35 min/72 h) | 20 μg/mL AST pretreatment of MSCs for 48 h, [i.v.], immediately after reperfusion; | ↑Renal function, ↓inflammation | ↑PTGS2 | (Geng et al., 2024) |
| Cardamonin |  | *In vivo*: C57BL/6 mice (male), UIRI/UNX (30 min/24 h);  *In vitro*: HK-2 cells, H/R (12 h/4 h) | 100 mg/kg, [p.o.], daily for 3 days before UIRI/UNX  100 μM, 24 h before H/R | ↑Renal function, oxidative stress, inflammation, and apoptosis | ↓MAPK/NF-κB | (Zhang et al., 2023) |
| Calycosin |  | *In vivo*: C57BL/6 mice (male), BIRI (35 min/24 h);  *In vitro*: HK-2 cells, H/R (24 h/3 h) | 5, 10, 20 mg/kg, [p.o.], daily for 7 days before BIRI;  8, 16, 32 μM, 24 h before H/R | ↓Renal injury and inflammation | ↑PPARγ, ↓EGR1 and NF-κB | (Zhang et al., 2022b) |
| Chrysin |  | *In vivo*: C57BL/6 mice (male), BIRI (30 min/48 h) | 100 mg/kg/d, [injection], for 3 days before BIRI; | ↑Renal function, ↓inflammation and apoptosis | ↓IκBα/NF-κB | (Xu et al., 2019) |
| Cyanidin-3-O-glucoside |  | *In vivo*: C57BL/6 mice (male), UIRI/UNX (30 min/24 h);  *In vitro*: HK-2 cells, H/R (12 h/6 h) | 50, 100, 200 mg/kg/d, [p.o.], for 2 weeks before UIRI/UNX;  50, 100, 150, 200 μM, 24 h before H/R | ↓Renal dysfunction, oxidative stress, apoptosis, and ERS | ↓JAK/STAT | (Xiong et al., 2023) |
| Diosmetin |  | *In vivo*: BALB/c mice (male), UIRI/UNX (45 min/24 h) | 0.25, 0.5, 1 mg/kg, [i.p.], 45 min before UIRI/UNX | ↓Renal dysfunction, oxidative stress, inflammation, and apoptosis | ↑Nrf2/HO-1; ↓NF-κB | (Yang et al., 2017) |
| Eriocitrin |  | *In vivo*: SD rats (male), BIRI (60 min/24 h);  *In vitro*: HK-2 cells, H/R (1.5 h/6 h) | 10, 30, 60 mg/kg/d, [i.p.];  1, 2, 4 μM, 30 min before H/R and maintained during H/R | ↓Renal injury, oxidative stress, apoptosis | ↑DUSP14 and Nrf2, ↓NF-κB | (Xu et al., 2021) |

(*continued on next page*)

**Table 1.** (*continued*)

| **Metabolites** | **Structure** | **Model** **(Animal species, sex,** **I/R duration)/ Cells** | **Treatment dosage and duration** | **Effect** | **Mechanism** | **Ref** |
| --- | --- | --- | --- | --- | --- | --- |
| Fisetin |  | *In vivo*: Wistar rats (male), BIRI (45 min/24 h) | 20 mg/kg, [i.p.], 15–30 min before BIRI | ↓Renal injury, oxidative stress, ferroptosis, ↑ mitochondrial function | ↓GSK3β | (Prem and Kurian, 2022) |
| Genistein |  | *In vivo*: SD rats (male), BIRI (28 min/24 h); | 15 mg/kg, [i.p.], 30 min before I/R | ↓Renal dysfunction and oxidative stress | ↓TLR4/NF-κB | (Gholampour et al., 2020) |
|  |  | *In vivo*: BALB/c mice (male), UIRI/UNX (45 min/24 h);  *In vitro*: NRK-52E cells, H/R (6h /6 h) | 2, 4 μg/mL, [i.v.], once daily for 7 days before UIRI/UNX  4 μg/mL, 2 h before H/R | ↓apoptosis and oxidative stress | ↑ADORA2A | (He et al., 2023) |
|  |  | *In vivo*: BALB/c mice (male), UIRI/UNX (45 min/24 h); | 10, 15 mg/kg, [i.v.], 30 min before ischemia | ↓renal apoptosis and tubular injury | ↑SIRT1 | (Li et al., 2017) |
| Hesperidin |  | *In vivo*: SD rats (male), BIRI (60 min/24 h) | 50, 100 mg/kg, [i.p.], 30 min before BIRI | ↓renal and lung oxidative injury; ↓ inflammation | ↓NF-κB | (Karacaer et al., 2023) |
|  |  | *In vivo*: SD rats (male), UIRI (45 min/14 d) | 10, 20 mg/kg, [i.v.], daily for 14 days after UIRI | ↓renal dysfunction, oxidative stress, inflammation and apoptosis | ↓TLR-4/NF-κB/iNOS | (Meng et al., 2020) |
|  |  | *In vivo*: SD rats (male), BIRI (30 min/24 h) | 100 mg/kg/d, [p.o.], daily for 7 days before BIRI | ↓renal dysfunction and oxidative stress | NR | (Park et al., 2019) |
| Hydroxysafflor Yellow A |  | *In vivo*: SD rats (male), UIRI (45 min/24 h);  *In vitro*: HK-2 cells, H/R (24 h) | 92 mg/kg, [i.v.], 0.5 h before UIRI/UNX;  5, 10, 50 μM, 24 h before H/R | ↓Renal dysfunction, tubular injury, apoptosis, and inflammation | ↓TLR4/NF-κB | (Bai et al., 2018) |
|  |  | *In vivo*: C57BL/6 mice (male), UIRI (30 min/24 h);  *In vitro*: HK-2 cells, H/R (12 h/2 h) | 100 mg/kg/d, [i.p.], 7 days before UIRI/UNX;  5, 10, 20 μg/mL, 12 h before H/R | ↓Renal dysfunction, tubular injury, oxidative stress, inflammation, and apoptosis | ↑Akt/GSK-3β/Fyn-Nrf2 | (Wang et al., 2022a) |

(*continued on next page*)

**Table 1.** (*continued*)

| **Metabolites** | **Structure** | **Model** **(Animal species, sex,** **I/R duration)/ Cells** | | **Treatment dosage and duration** | | | **Effect** | **Mechanism** | | **Ref** | |
| --- | --- | --- | --- | --- | --- | --- | --- | --- | --- | --- | --- |
| Hyperoside |  | *In vivo*: C57BL/6 mice (male), UIRI (30 min/24 h);  *In vitro*: HK-2 cells, CoCl₂-induced hypoxia (12 h/24 h) | | 20 mg/kg, [i.p.], 2 h before UIRI  50, 100, 150, 200 μM, 1 h before CoCl₂ exposure | | | ↓Renal injury, modulates mitochondrial fission, oxidative stress, and apoptosis | ↓OMA1 mediated OPA1 proteolysis | | (Wu et al., 2019) | |
| Isoquercitrin |  | *In vivo*: C57BL/6 mice (male), UIRI/UNX (30 min/24 h) | | 20 mg/kg, [i.p.], daily for 3 days before UIRI/UNX | | | ↓ Renal injury, ↓ oxidative stress, apoptosis, and inflammation | NR | | (Liang et al., 2020) | |
| Kaempferide |  | *In vivo*: C57BL/6 mice (male), BIRI (40 min/24 h);  *In vitro*: HK-2 cells, H/R (10 h/12 h) | | 50 mg/kg, [p.o.], 2 h after renal vascular ligation  10 μM, during H/R | | | ↓ Renal damage, ↑ autophagy, ↓oxidative stress, and inflammation | Modulates AMPK/AKT | | (Shao et al., 2023) | |
| Luteolin |  | *In vivo*: SD rats (male), UIRI (45 min/24 h) | | 40 mg/kg/d, [p.o.], daily for 3 days before UIRI and continued for 1 day after UIRI | | | ↓ Renal dysfunction, tubular injury, oxidative stress, inflammation, apoptosis | ↓HMGB1/NF-κB | | (Hong et al., 2017) | |
|  |  | *In vivo*: SD rats (male), BIRI (45 min/2 h) | | 50 mg/kg/d, [p.o.], daily for 3 days before BIRI | | | ↓ Renal dysfunction, tubular injury, and oxidative stress | ↓Nrf2 | | (Kalbolandi et al., 2019) | |
|  |  | *In vivo*: SD rats (male), UIRI (not specified/30 d) | | 50 mg/kg/d, [p.o.], daily from Day 1 after UIRI for 30 days | | | ↓ Renal dysfunction, tubular injury, oxidative stress, inflammation, apoptosis, and fibrosis | ↑Nrf2/HO-1 | | (Wei et al., 2025) | |
| Loureirin C |  | | *In vivo*: C57BL/6 mice (male), UIRI/UNX (45 min/24 h);  *In vitro*: HK-2 cells, H/R (24 h/12 h) | | 10, 25, 50 mg/kg/d, [p.o.], 3 days before UIRI/UNX;  0.1, 0.5, 5, 10, 20 μM, 24 h before H/R | ↓ Renal damage, ↓oxidative stress, ↑ mitochondrial function | | | ↑Nrf2, ↑ HO-1/NQO-1 | | (Qi et al., 2024) |

(*continued on next page*)

**Table 1.** (*continued*)

| **Metabolites** | **Structure** | **Model** **(Animal species, sex,** **I/R duration)/ Cells** | **Treatment dosage and duration** | **Effect** | **Mechanism** | **Ref** |
| --- | --- | --- | --- | --- | --- | --- |
| Naringenin |  | *In vivo*: C57BL/6 mice (male), UIRI (30 min/24 h);  *In vitro*: HK-2 cells, H/R (12 h/4 h) | 50 mg/kg/d, [p.o.], daily for 3 days before UIRI;  200 μM, 24 h before H/R | ↓Renal dysfunction, ↓ oxidative stress, apoptosis, and pyroptosis | ↑Nrf2/HO-1 | (Zhang et al., 2022a) |
| Naringin |  | *In vivo*: SD rats (male), BIRI (45 min/4 h) | 100 mg/kg/d, [i.p.], 7 days before BIRI; Trimetazidine 5 mg/kg, [i.v.], 5 min before reperfusion | ↓Renal and cardiac oxidative stress; ↓ remote myocardial injury | ↑Nrf2 | (Amini et al., 2019a) |
|  |  | *In vivo*: SD rats (male), BIRI (45 min/4 h); | 100 mg/kg/d, [i.p.], 7 days before BIRI; Trimetazidine 5 mg/kg, [i.v.], 5 min before reperfusion | ↓Renal dysfunction, oxidative stress, and apoptosis; ↑ renal hemodynamics | ↓miR-10a | (Amini et al., 2019b) |
|  |  | *In vivo*: Wistar rats (male), UIRI/UNX (45 min/72 h) | 50, 100 mg/kg/day, [i.p.], at the beginning of reperfusion, 24 h and 48 h later | ↓Renal apoptosis, tubular injury, and IL-1β-mediated inflammation | ↑HIF-1α | (Danis et al., 2024) |
| Nobiletin |  | *In vivo*: Wistar rats (male), UIRI (45 min/3 h) | 10 mg/kg, [p.o.], daily for 7 days before UIRI | ↓Renal dysfunction, tubular injury, oxidative stress, and inflammation | ↓iNOS/eNOS | (Güvenç et al., 2020) |
|  |  | *In vivo*: C57BL/6J mice (male), UIRI/UNX (45 min/24 h); | 25, 50, 100 mg/kg, [i.p.], daily for 7 days before UIRI/UNX | ↓Renal dysfunction, tubular injury, ER stress-related apoptosis, and oxidative stress | ↑PI3K/AKT | (Liu et al., 2020a) |
| Oroxylin A |  | *In vivo*: C57BL/6J mice (male), BIRI (35 min/24 h);  *In vitro*: HK-2 cells, H/R (24 h/6 h) | 20 mg/kg, [i.v.], after BIRI  10 μM, after H/R | ↓Kidney injury, ↑mitochondrial homeostasis | ↑PPARα-BNIP3 | (Yao et al., 2022) |

(*continued on next page*)

**Table 1.** (*continued*)

| **Metabolites** | **Structure** | **Model** **(Animal species, sex,** **I/R duration)/ Cells** | **Treatment dosage and duration** | **Effect** | **Mechanism** | **Ref** |
| --- | --- | --- | --- | --- | --- | --- |
| Proanthocyanidin B2 |  | *In vivo*: C57BL/6 mice (male), UIRI/UNX (45 min/24 h); | 30 mg/kg, [i.p.], 45 min before UIRI/UNX | ↓Renal dysfunction, tubular injury, mitochondrial damage, ER stress, and apoptosis | ↑Nrf2/HO-1 | (Wang et al., 2023) |
|  |  | *In vivo*: ICR mice (male), UIRI/UNX (45 min/24 h); | 30 mg/kg, [i.p.], 45 min before UIRI/UNX | ↓Renal dysfunction, tubular injury, oxidative stress, and apoptosis | ↑Nrf2/HO-1 | (Wang et al., 2020b) |
| Puerarin |    | *In vivo*: SD rats (male), UIRI/UNX (45 min/24 h);  *In vitro*: HK-2 cells, H/R (12 h/24 h) | 50, 100 mg/kg/d, [i.p.], 7 days before UIRI/UNX  1, 10 μM, once daily for 3 days before H/R | ↓ Kidney injury, ↓ apoptosis and ER stress | ↑Nrf2/HO-1 | (Wang et al., 2025) |
| Quercetin |  | *In vivo*: Wistar rats (male), UIRI (30 min/5 h); | 100 mg/kg, [p.o. or i.p.], 1 h before UIRI | ↓Renal dysfunction, oxidative stress, inflammation, and apoptosis | ↓NF-κB | (Bagheri et al., 2023) |
|  |  | *In vivo*: C57BL/6J mice (male), BIRI (30 min/24 h);  *In vitro*: NRK-52E/HK-2 cells, ferroptosis model | 25 mg/kg, [p.o.], before BIRI, 3 times/day  10 μM, co-treated with the ferroptosis inducer for 24 h | ↓Renal dysfunction, tubular injury, ferroptosis, and inflammation | ↓ATF3/SLC7A11 | (Wang et al., 2021) |
| Scutellarein |  | *In vivo*: Wistar rats (male), BIRI (45 min/24 h);  In vitro: HK-2 cells, H/R (24 h) | 50 mg/kg/d, [i.p.], 7 days before BIRI  20 μM, 12 h before H/R | ↓Renal dysfunction, tubular injury, and oxidative stress | ↑Nrf2/HO-1 | (Dai et al., 2022) |
|  |  | *In vivo*: SD rats (male), BIRI (30 min/48 h);  *In vitro*: HK-2 cells, H/R (24 h/6 h) | 5, 10, 20 mg/kg, [p.o.], every other day for 20 days before BIRI  2.5, 5, 10 μM, 24 h before OGD/R | ↓Renal dysfunction, tubular injury, apoptosis, and inflammation | NR | (Liu et al., 2021) |

(*continued on next page*)

**Table 1.** (*continued*)

| **Metabolites** | **Structure** | **Model** **(Animal species, sex,** **I/R duration)/ Cells** | **Treatment dosage and duration** | **Effect** | **Mechanism** | **Ref** |
| --- | --- | --- | --- | --- | --- | --- |
| Scutellarin |  | *In vivo*: C57BL/6J mice (male), UIRI/UNX (45 min/24 h);  *In vitro*: RAW 264.7 cells, BMDMs (24 h) | 5, 25, 50 mg/kg, [p.o.], 1 h before UIRI;  0–150 μM screened; 150 μM for mechanism study, 24 h before H/R | ↓Renal injury, inflammation, macrophage polarization | ↓MAPK | (Deng et al., 2024a) |
| Silibinin |  | *In vivo*: C57BL/6J mice (male), UIRI/UNX (45 min/24 h) | 200 μL intravenous SLB-HP-β-CD before reperfusion | ↓Ferroptosis | Targets NCOA4-FTH1 | (Deng et al., 2024b) |
|  |  | *In vivo*: Wistar rats (male), hepatic I/R-induced remote renal injury (45 min/60, 120, 180, 240 min) | 200 μL intravenous SLB-HP-β-CD before reperfusion | ↓Renal injury and dysfunction, ↓ inflammation, and ferroptosis | ↓NF-κB | (Kyriakopoulos et al., 2018) |
| Tilianin |  | *In vivo*: C57BL/6 mice (male), BIRI (35 min/24 h); | 5, 10, 15 mg/kg/d, [p.o.], 7 days before BIRI | ↓Renal dysfunction, tubular injury, and apoptosis | ↓ERK/EGR1 | (Liu et al., 2022) |
|  |  | *In vivo*: SD rats (male), BIRI (30 min/2 h) | 5 mg/kg, [i.p.], 30 min before BIRI | ↓Renal dysfunction, inflammation, oxidative stress, and apoptosis | NR | (Hameed Ali et al., 2023) |
| Tiliroside |  | *In vivo*: C57BL/6J mice (male), BIRI (35 min/24 h); | 30 mg/kg/d, [p.o.], for 48 h before BIRI; | ↓Renal injury and ferroptosis | ↑Nrf2/GPX4 | (Cai et al., 2024) |
| Xanthohumol |  | *In vivo*: SD rats (male), BIRI (45 min/24 h);  *In vitro*: HK-2 cells, H/R (8 h/4 h) | 0.4 mg/kg, [i.p.], 10 min before BIRI;  10 μM, at the beginning of reoxygenation | ↓Renal injury, ferroptosis | ↑Nrf2/HO-1 | (Tang et al., 2023a) |

(*continued on next page*)

**Table 1.** (*continued*)

| **Metabolites** | **Structure** | **Model** **(Animal species, sex,** **I/R duration)/ Cells** | **Treatment dosage and duration** | **Effect** | **Mechanism** | **Ref** |
| --- | --- | --- | --- | --- | --- | --- |
| **Saponins** |  |  |  |  |  |  |
| Astragaloside IV |  | *In vivo*: SD rats (male), UIRI/UNX (45 min/24 h and 14 d) | 2, 5, 10 mg/kg, [p.o.], 1 h before UIRI/UNX | ↓Kidney injury; ↓ oxidative stress and apoptosis | ↑Nrf2/HO-1 | (Su et al., 2022) |
| Clemaichinenoside |  | *In vitro*: HK-2 cells, H/R (6 h/6 h) | 0, 20 μM, 2 h before H/R | ↑Cell viability, ↓apoptosis, oxidative stress, and inflammation | ↑Nrf2/HO-1 | (Feng et al., 2020) |
| Ginsenoside Rd |  | *In vivo*: C57BL/6 mice (male), BIRI (25 min/Day 1, 3, 5, 7);  *In vitro*: M1 macrophages (24 h) | 10, 20, 50, 100 mg/kg, [i.p.], 30 min before BIRI;  10, 20, 50, 100 μg/mL, during M1 polarization | ↓Renal injury and inflammation | ↓M1 polarization | (Ren et al., 2016) |
| Gypenoside |  | *In vivo*: C57BL/6 mice (male), UIRI/UNX (NR/6 h) | 50 mg/kg, [i.v.], 1 h before UIRI/UNX | ↓Renal dysfunction; ↓inflammation and oxidative stress | ↓ ERK signaling | (Ye et al., 2016) |

(*continued on next page*)

**Table 1.** (*continued*)

| **Metabolites** | **Structure** | **Model** **(Animal species, sex,** **I/R duration)/ Cells** | **Treatment dosage and duration** | **Effect** | **Mechanism** | **Ref** |
| --- | --- | --- | --- | --- | --- | --- |
| Gypenoside XLIX |  | *In vivo*: C57BL/6 mice (male), BIRI (40 min/24 h);  *In vitro*: HK-2 cells, H/R (24 h/6 h) | 50 mg/kg, [i.p.], 30 min before BIRI;  32, 64, 128 μM screened; 64 μM for mechanism study, 24 h before H/R | ↓Renal dysfunction, ↓ inflammation and programmed cell death | Modulates IGFBP7/IGF1R | (Yang et al., 2021) |
| Gypenoside XVII |  | *In vivo*: ICR mice (male), BIRI (40 min/24 h) | 10, 20, 40 mg/kg/d, [p.o.], 7 days before BIRI | ↓Renal injury, ↓ pyroptosis and endoplasmic reticulum stress | ↓NLRP3 inflammasome activation | (Wang et al., 2024) |
| Madecassoside |  | *In vivo*: C57BL/6 mice (male), BIRI (40 min/24 h) | 40 mg/kg, i.p., 12 h before I/R | ↓Kidney damage, inflammation, apoptosis, and oxidative stress | ↓JNK/c-JUN | (Shan et al., 2024) |
| Notoginsenoside R1 |  | *In vivo*: Wistar rats (male), UIRI/UNX (45 min/72 h) | 50 mg/kg, [p.o.], 30 min before UIRI/UNX | ↓Renal dysfunction, ↓ inflammation and oxidative stress | NR | (Fan et al., 2020) |

(*continued on next page*)

**Table 1.** (*continued*)

| **Metabolites** | **Structure** | **Model** **(Animal species, sex,** **I/R duration)/ Cells** | **Treatment dosage and duration** | **Effect** | **Mechanism** | **Ref** |
| --- | --- | --- | --- | --- | --- | --- |
| Sodium Aescinate |  | *In vivo*: C57BL/6 mice (male), BIRI (40 min/24 h);  *In vitro*: NRK cells, H/R (2 h/24 h) | 2.5, 5 mg/kg, [i.p.], 3 days before BIRI;  20, 40, 80 μM screened; 40 μM for mechanism study, 24 h before H/R | ↓Kidney injury; ↓ pyroptosis, ↓inflammation and macrophage infiltration | ↓AKT/NLRP3 | (Liu et al., 2025b) |
| **Alkaloids** |  |  |  |  |  |  |
| Anisodamine |  | *In vivo*: SD rats (male), UIRI (45 min/24 h) | 1 mg/kg, [i.v.], 10 min before reperfusion | ↑Renal function and histology; ↓apoptosis inflammation | ↑ ERK | (Zhang et al., 2021) |
| Berberine |  | *In vivo*: SD rats (male), BIRI (60 min/24 h) | 150 mg/kg/d, [p.o.], for 14 days before BIRI | ↓Renal inflammation and damage | ↓Sirt1/NF-κB/TLR4; intestinal microbiota modulation | (Huo and Wang, 2024) |
|  |  | *In vitro*: NRK-52E cells, H/R (6 h/2 h) | 10 μM for mechanism study, 2 h before H/R | ↓ Apoptosis | Modulates Sirt1/p53 mitochondrial | (Lin et al., 2018) |
|  |  | *In vitro*: HK-2 cells H/R (6 h/6 h) | 1, 5, 10 μM screened; 10 μM for mechanism study, 24 h before H/R | ↓ Apoptosis | ↓ SPHK1/p38 MAPK/mitochondrial | (Lu et al., 2018) |
| Evodiamine |  | *In vivo*: Wistar rats (male), UIRI (60 min/24 h) | 10 mg/kg, [i.p.], 30 min before reperfusion | ↑ Renal function; ↓inflammation and apoptosis | ↓ NF-κB | (Eraslan et al., 2019) |
| Leonurine |  | In vivo: SD rats (male), BIRI (45 min/24 h) | 7.5, 15, 30 mg/kg/d, [p.o.], 7 days before BIRI | ↑ Renal function; ↓ oxidative stress and inflammation | ↑ Nrf2 nuclear translocation, ↓ TLR4/NF-κB | (Han et al., 2022) |

(*continued on next page*)

**Table 1.** (*continued*)

| **Metabolites** | **Structure** | **Model** **(Animal species, sex,** **I/R duration)/ Cells** | **Treatment dosage and duration** | **Effect** | **Mechanism** | **Ref** |
| --- | --- | --- | --- | --- | --- | --- |
| Neferine |  | *In vivo*: C57BL/6 mice (male), BIRI (30 min/24 h)  *In vitro*: NRK-52E cells H/R (2 h/12 h) | 20 mg/kg/d, [i.p.], 3 days before BIRI;  1–100 μM screened; 2,4,8 μM for mechanism study, 2 h before H/R | ↓ Kidney injury, ↓inflammation and apoptosis | ↓ NF-κB, ↑ Klotho | (Li et al., 2019) |
| Nuciferine |  | *In vivo*: Wistar rats (male), BIRI (35 min/72 h) | 30 mg/kg/d, [p.o.], 9 days before BIRI and continued for 3 days after BIRI | ↑Renal function; ↓ inflammation, fibrosis, and apoptosis | ↓NF-κB | (Sallabi et al., 2025) |
| Oxymatrine |  | *In vivo*: SD rats (male), UIRI/UNX (45 min/24 h) | 40 mg/kg, [i.v.], 10 min before reperfusion | ↑Renal function; ↓ oxidative stress | NR | (Ozturk et al., 2017) |
| Piperine |  | *In vivo*: Wistar rats (male), UIRI/UNX (30 min/24 h) | 10, 20 mg/kg/d, [p.o.], 10 days before IRI | ↑ Renal function; ↓ oxidative stress and inflammation | NR | (Mohammadi et al., 2020) |
| Piperlongumine |  | *In vivo*: SD rats (male), BIRI (1h/2h) | 25, 50 μg/kg, [i.p.], 30 min before IRI | ↑ Renal function; ↓ oxidative stress, inflammation | Modulates COX-2/eNOS | (Xiao and Vijayalakshmi, 2022) |
| Rutaecarpine |  | *In vivo*: SD rats (male), UIRI (60 min/24 h) | 30, 60 mg/kg, [i.p.], 10 min before reperfusion | ↑ Renal function; ↓ tubular necrosis, inflammation, and oxidative stress | ↓ JNK/p38 MAPK | (Wang et al., 2017) |
| Tetramethylpyrazine (Ligustrazine) |  | *In vivo*: SD rats (male), UIRI (50 min/24 h)  *In vitro*: NRK-52E cells H/R (2 h/24 h) | 40 mg/kg, [i.p.], every 6 h during reperfusion;  50 μM, after H/R for 24 h | ↓ Renal inflammation and apoptosis | ↑Autophagy, ↓ NOD2 | (Jiang et al., 2020) |
|  |  | *In vivo*: SD rats (male), BIRI (45 min/24 h); | 40 mg/kg, [i.p.], immediately after reperfusion, every 6 h; | ↓ Renal tubular apoptosis | ↓ NLRP3/HIF-1α | (Sun et al., 2020) |

(*continued on next page*)

**Table 1.** (*continued*)

| **Metabolites** | **Structure** | **Model** **(Animal species, sex,** **I/R duration)/ Cells** | **Treatment dosage and duration** | **Effect** | **Mechanism** | **Ref** |
| --- | --- | --- | --- | --- | --- | --- |
| **Terpenoids** |  |  |  |  |  |  |
| Astaxanthin |  | *In vivo*: Wistar rats (male), UIRI/UNX (45 min/24h); | 50, 100 mg/kg/d, [p.o.], 7 days before UIRI/UNX | ↓tubular injury, apoptosis, inflammation, oxidative stress | NR | (Arslan et al., 2021) |
|  |  | *In vivo*: SD rats (male), BIRI (45 min/24 h); | 5, 10, 25 mg/kg/d, [p.o.], 7 days before BIRI | ↓ Renal injury, tubular damage, oxidative stress | NR | (Kisaoglu et al., 2024) |
| Carnosol |  | *In vivo*: SD rats (male), UIRI/UNX (45 min/24 h) | 3 mg/kg, [i.v.], 1 h before UIRI/UNX | ↓ Renal damage; ↓inflammation and apoptosis | ↑p38 MAPK | (Zheng et al., 2018) |
| Celastrol |  | *In vivo*: Swiss albino rats (male), BIRI (30 min/24h) | 2 mg/kg, [p.o.], daily for 1 week before BIRI | ↓ Renal damage, ↓ inflammation and oxidative stress | ↑Nrf2/HO-1, PI3K/AKT; ↓ NF-κB | (Younis and Ghanim, 2022) |
| Crocin |  | *In vivo*: SD rats (male), UIRI (45 min/24 h) | 20 mg/kg, [p.o.], daily for 7 days before UIRI | ↓ Renal dysfunction, oxidative stress and inflammation | ↓ TLR4 | (Abou-Hany et al., 2018) |
|  |  | *In vivo*: Wistar rats (male), BIRI (30 min/24 h) | 100, 200, 400 mg/kg, [i.p.], 30 min before BIRI | ↓ Renal injury | NR | (Yarijani et al., 2017) |
| Costunolide |  | *In vivo*: Wistar albino rats (male), BIRI (45 min/24 h) | 5, 10 mg/kg, [i.p.], 30 min before reperfusion | ↓ Renal damage, ↓ autophagy, apoptosis, inflammation, and DNA damage | NR | (Guler et al., 2023) |
| Fucoxanthin |  | *In vivo*: C57BL/6 mice (male), UIRI/UNX (45 min/24 h)  *In vitro*: HK-2 cells H/R (6 h) | 25, 50, 100 mg/kg, [i.p.], 30 min before BIRI  0.1, 1, 5 μM screened; 5 μM for mechanism, 24 h before H/R | ↓ Oxidative damage, ↑renal function ↓ROS, apoptosis | ↑Sirt1/Nrf2/HO-1 | (Mao et al., 2022) |

(*continued on next page*)

**Table 1.** (*continued*)

| **Metabolites** | **Structure** | **Model** **(Animal species, sex,** **I/R duration)/ Cells** | **Treatment dosage and duration** | | **Effect** | **Mechanism** | **Ref** |  |
| --- | --- | --- | --- | --- | --- | --- | --- | --- |
| Ganoderic Acids |  | *In vivo*: C57BL/6J (male), UIRI/UNX (48h/24h)  *In vitro*: NRK-52E cells, H/R (12h /12 h) | 1.7, 5, 15 mg/kg/d, [i.p.], 30 min before UIRI/UNX  3.125, 12.5, 50 mg/mL. 12 h before the serum-deprived | | ↓ Renal damage, inflammation and apoptosis | ↓TLR4/MyD88/NF-κB | (Shao et al., 2021) |  |
| Geraniol |  | *In vivo*: Wistar rats (male), BIRI (45 min/5 h) | 100, 200 mg/kg, [p.o.], daily for 14 days before BIRI | ↑Renal function, ↓inflammation and apoptosis | | ↓TLR/MyD88/NF-κB; ↑Nrf2/HO-1 | (Mohamed et al., 2022a) | |
| Glaucocalyxin A |  | *In vitro*: HK-2 cells (24h/2h) | 0, 5, 10, 20, 40 μM screened; 5 μM, 2 h before H/R | | ↑Cell viability, ↓ROS and apoptosis | ↑Akt/Nrf2/HO-1 | (Hosohata et al., 2021) | |
| Loganin |  | *In vivo*: C57BL/6 (male), UIRI/UNX (30min/24h)  *In vitro*: NRK-52E cells | 5, 10, 20 mg/kg, [i.p.], for 1 week before UIRI/UNX  10, 20, 40 μM, 4 h before H/R | | ↓Renal damage, ↓ apoptosis, inflammation and oxidative stress | ↓JAK2/STAT3; ↑ Nrf2/HO-1 | (Huang et al., 2022) | |
| Lupeol |  | *In vivo*: Wistar rats (female), BIRI (45 min/24h) | 100 mg/kg, [i.p.], 1 h before BIRI | | ↑ Renal function, ↓ oxidative stress and inflammation | NR | (Kapisiz et al., 2024) | |
| Lycopene |  | *In vivo*: SD rats (male), UIRI/UNX (45 min/24 h) | 100 mg/kg, gavage, once daily for 7 days before I/R | | ↓renal damage, oxidative stress and inflammation | ↓MAPK | (Pan et al., 2024) | |
| Madecassoside |  | *In vivo*: C57BL/6 mice (male), BIRI (40 min/24 h) | 40 mg/kg, i.p., 12 h before I/R | | ↓Kidney damage, inflammation, apoptosis, and oxidative stress | ↓JNK/c-JUN | (Shan et al., 2024) | |

(*continued on next page*)

**Table 1.** (*continued*)

| **Metabolites** | **Structure** | **Model** **(Animal species, sex,** **I/R duration)/ Cells** | **Treatment dosage and duration** | **Effect** | **Mechanism** | **Ref** |
| --- | --- | --- | --- | --- | --- | --- |
| Myrtenal |  | *In vivo*: SD rats (male), BIRI (45 min/24 h) | 40, 80 mg/kg/d, [i.p.], 9 days before I/R | ↓ Renal damage, oxidative stress, inflammation, and apoptosis | NR | (Beytur et al., 2025) |
| Pachymic acid |  | *In vivo*: Wistar rats (male), BIRI (30 min/24 h) | 5, 10, 20 mg/kg, [i.p.], 3 days before I/R | ↓ Renal dysfunction, oxidative stress, and ferroptosis | ↑ Nrf2, GPX4, SLC7A11, HO-1 | (Jiang et al., 2021) |
| Perillyl alcohol |  | *In vitro*: HK-2 cells, H/R (24 h /12 h) 25–100 μM, 2 h before H/R | 25–100 μM, 2 h before H/R | Protects renal cells, ↓ ROS, ER stress, and apoptosis | ↑PI3K/Akt/eNOS | (Xu et al., 2017) |
| Poricoic acid A |  | *In vivo*: SD rats (male), IRI (1h /24 h)  *In vitro*: NRK-52E cells (9h/6h) | 10 mg/kg  10 µM | ↓ Renal fibrosis, podocyte injury, oxidative stress, and inflammation | ↓ NF-κB, Nrf2, and Gas6/Axl | (Chen et al., 2019) |
| Picroside II |  | *In vivo*: C57BL/6J mice (male), BIRI (35 min/24 h) | 20 mg/kg [i.p.] 24 h before ischemia | ↑ Renal microcirculation, ↓inflammation, and oxidative stress | ↓ TLR4/NF-κB | (Ren et al., 2024) |
| Taraxasterol |  | *In vivo*: C57BL/6 mice (male), BIRI (30 min/72 h)  *In vitro*: HK-2 cells, H/R (12h/2h) | 5 & 10 mg/kg in vivo; 5 & 10 µM in vitro | ↓Renal damage, inflammation, and apoptosis | ↓MAPK | (Li et al., 2020) |
| Tanshinone IIA |  | *In vivo*: SD rats (male), UIRI/UNX (25 min/10 days) | 25 mg/kg, [i.p.], daily for 10 days before I/R | ↓ Renal damage, inflammation, and oxidative stress | ↓p38 MAPK | (Xu et al., 2016) |
| Ursolic acid |  | *In vivo*: SD rats (male), UIRI/UNX (45–90 min/4 days) | 10 mg/kg, [i.p.], twice daily for 4 days before I/R | ↑ Renal function, ↓oxidative stress and inflammation | ↓STAT3, NF-κB, | (Peng et al., 2016) |

(*continued on next page*)

**Table 1.** (*continued*)

| **Metabolites** | **Structure** | **Model** **(Animal species, sex,** **I/R duration)/ Cells** | **Treatment dosage and duration** | **Effect** | **Mechanism** | **Ref** |
| --- | --- | --- | --- | --- | --- | --- |
| **Others** |  |  |  |  |  |  |
| Apocynin |  | *In vivo*: SD rats (male), BIRI (45 min/22 h) | 50 mg/kg, [i.p.], 30 min before I/R | ↓ Renal damage, oxidative stress and inflammation | Modulates zinc and metallothionen expression | (Hu et al., 2017) |
| Allicin |  | *In vivo*: SD rats (male), UIRI (45 min/12 h);  *In vitro*: NRK-52E cells, H/R (24 h/6 h) | 40, 50, 60 mg/kg, [i.p.], before I/R;  1, 3, 5 μM during H/R | ↓ Renal injury | NR | (Li et al., 2022) |
|  |  | *In vivo*: SD rats (male), BIRI (45 min/22 h) | 50 mg/kg, [i.p.], 30 min before I/R | ↓ Renal injury | NR | (Shan et al., 2021) |
| Cordycepin |  | *In vivo*: SD rats (female), UIRI (60 min/4 h) | 10 mg/kg, [i.p.], after reperfusion | ↓ Renal dysfunction, ↓ oxidative stress | NR | (Aydin et al., 2020) |
|  |  | *In vivo*: C57BL/6J mice (male), BIRI (30 min/24 h) | 10 mg/kg, [p.o.], every 2 days for 1 week before I/R | ↓ Renal dysfunction, ↓ oxidative stress | ↓ p38/JNK | (Chen et al., 2025) |
|  |  | *In vivo*: SD rats (male), UIRI/UNX (45 min/24 h) | 2, 4, 8 mg/kg/d, [p.o.], 7 days before I/R | ↓ Oxidative stress, inflammation, apoptosis, and ferroptosis | ↓ TLR4/MyD88 | (Han et al., 2020) |
| Chitosan oligosaccharide |  | *In vivo*: C57BL/6J mice (male), UIRI/UNX (35 min/2 days);  *In vitro*: TCMK-1 cells, tBHP-induced oxidative stress | 100, 200 mg/kg/d, [p.o.], 3 days before I/R until sacrifice;  200 μg/mL pre-treatment | ↓ Renal injury | ↑ Pam16, Trem 2, and Esrra | (Yin et al., 2023) |
| Diosgenin |  | *In vivo*: C57BL/6J mice (male), UIRI/UNX (45 min/21 days)  *In vitro*: HK-2 cells, H/R (6 h/1 h) | 1 or 10 mg/kg/day, [p.o.] 21 days post I/R  0.1 & 10 μM for 1 h | ↑Kidney function, ↓ fibrosis and inflammation | ↓ NOX4/p65 | (Chiang et al., 2024) |
| Emodin |  | *In vitro*: HK-2 cells, H/R (24 h/12 h) | 10–50 μM pre-treatment 2 h before H/R | ↑ Cell viability, ↓ Apoptosis | ↓ MAPK (ERK, JNK, p38) | (Chen et al., 2017) |

(*continued on next page*)

**Table 1.** (*continued*)

| **Metabolites** | **Structure** | **Model** **(Animal species, sex,** **I/R duration)/ Cells** | **Treatment dosage and duration** | **Effect** | **Mechanism** | **Ref** |
| --- | --- | --- | --- | --- | --- | --- |
|  |  | *In vivo*: SD rats (male), BIRI (45 min/24 h);  *In vitro*: HK-2 cells, H/R 12 h/8 h | 1, 3, 10 mg/kg/day, [i.p.], 7 days before I/R  10–80 μM for 4–48 h | ↑Renal function, ↓apoptosis and oxidative stress, ↓ mitochondrial fission, ↑ angiogenesis | ↓ p53/caspase-3/9 | (Lu et al., 2023) |
|  |  | *In vivo*: C57BL/6 mice (male), UIRI/UNX (35 min/24 h);  *In vitro*: HK-2 cells, H/R 12 h/8 h | 1, 3, 10 mg/kg/day, [i.p.], 7 days before I/R  1, 3, 10 μM 24 h pre-H/R | ↓Renal injury, ↓apoptosis and oxidative stress | ↓CAMKII , ↓ DRP1-Ser616 | (Wang et al., 2022b) |
| Gastrodin |  | *In vivo*: SD rats (male), UIRI/UNX (45 min/6–24 h) | 300 mg/kg/day, [p.o.] 8 days before I/R | ↓Renal injury, ↓oxidative stress and apoptosis | ↓ p38 MAPK | (Zheng et al., 2022) |
| Honokiol |  | *In vivo*: C57BL/6 mice (male), BIRI (25 min/24 h);  In vitro: HK-2 cells | 1 mg/kg, [i.p.], 1 h before ischemia, 4 h after reperfusion;  2–10 μM | ↓Apoptosis, neutrophil infiltration, ↓ peroxidation | ↑ PI3K/Akt and PKC, ↑ Nrf2 translocation | (Park et al., 2020) |
| Paeoniflorin |  | *In vivo*: C57BL/6 mice (male), BIRI (40 min/24 h);  In vitro: HK2 cells, H/R | 25–100 mg/kg/d, [i.p.], 3 days before I/R;  6.25–100 μM pre-treatment | ↓Renal dysfunction, tubular injury, inflammation, and ferroptosis | ↑ SLC7A11/GPX4 axis | (Ma et al., 2023) |
| Shikonin |  | *In vivo*: C57BL/6 mice (male), UIRI/UNX (45 min/24 h);  *In vitro*: HK-2 cells, H/R (12 h/2 h) | 60 mg/kg, [i.p.], 24 h pre-I/R;  100 μM, 12 h pre-H/R | ↑Renal function, ↓ apoptosis, and ER stress | ↑SIRT1/Nrf2/HO-1 | (Huang et al., 2025) |

Note: BIRI (Bilateral ischemia-reperfusion injury); IRI (Ischemia-reperfusion injury); UIRI (Unilateral ischemia-reperfusion injury); UIRI/UNX (Unilateral Ischemia-Reperfusion Injury combined with Uninephrectomy); H/R (Hypoxia/Reoxygenation); HK-2 (Human renal tubular epithelial cells); SD rats (Sprague–Dawley rats); NR (not reported); NRK (rat renal tubular epithelial cell line); TCMK-1 (mouse renal tubular epithelial cell line); PTC (primary tubular epithelial cells)

**References**

Abou-Hany, H. O., Atef, H., Said, E., Elkashef, H. A., and Salem, H. A. (2018). Crocin reverses unilateral renal ischemia reperfusion injury-induced augmentation of oxidative stress and toll like receptor-4 activity. *Environ. Toxicol. Pharmacol.* 59, 182-189. doi: 10.1016/j.etap.2018.03.017.

Ahmadvand, H., Yalameha, B., Adibhesami, G., Nasri, M., Naderi, N., Babaeenezhad, E., et al. (2019). The protective role of gallic acid pretreatment on renal ischemia-reperfusion injury in rats. *Rep. Biochem. Mol. Biol.* 8 (1), 42-48.

Alaasam, E. R., Janabi, A. M., Al-Buthabhak, K. M., Almudhafar, R. H., Hadi, N. R., Alexiou, A., et al. (2024). Nephroprotective role of resveratrol in renal ischemia-reperfusion injury: a preclinical study in Sprague-Dawley rats. *BMC Pharmacol. Toxicol.* 25 (1), 82. doi: 10.1186/s40360-024-00809-8.

Alechinsky, L., Favreau, F., Cechova, P., Inal, S., Faye, P. A., Ory, C., et al. (2020). Tannic acid improves renal function recovery after renal warm ischemia-reperfusion in a rat model. *Biomolecules* 10 (3). doi: 10.3390/biom10030439.

Amini, N., Sarkaki, A., Dianat, M., Mard, S. A., Ahangarpour, A., and Badavi, M. (2019a). Protective effects of naringin and trimetazidine on remote effect of acute renal injury on oxidative stress and myocardial injury through Nrf-2 regulation. *Pharmacol. Rep.* 71 (6), 1059-1066. doi: 10.1016/j.pharep.2019.06.007.

Amini, N., Sarkaki, A., Dianat, M., Mard, S. A., Ahangarpour, A., and Badavi, M. (2019b). The renoprotective effects of naringin and trimetazidine on renal ischemia/reperfusion injury in rats through inhibition of apoptosis and downregulation of micoRNA-10a. *Biomed. Pharmacother.* 112. doi: 10.1016/j.biopha.2019.01.029.

Arslan, E., Turk, H., Caglayan, M., Turkmenoglu, T. T., Gonel, A., and Tayman, C. (2021). Protective effects of astaxanthin on nephrotoxicity in rats with induced renovascular occlusion. *Comb. Chem. High. Throughput Screen.* 24 (8), 1236-1242. doi: 10.2174/1386207323666200914104432.

Aydin, H. R., Sekerci, C. A., Yigit, E., Kucuk, H., Kocakgol, H., Kartal, S., et al. (2020). Protective effect of cordycepin on experimental renal ischemia/reperfusion injury in rats. *Arch. Ital. Urol. Androl.* 92 (4). doi: 10.4081/aiua.2020.4.340.

Bagheri, A., Radman, G., Aria, N., Rezaei, F., Khajenouri, M., Ghiabi, S., et al. (2023). The effects of quercetin on apoptosis and antioxidant activity in a renal ischemia/reperfusion injury animal model. *Drug Res. (Stuttg)* 73 (5), 255-262. doi: 10.1055/a-1999-7600.

Bai, J., Zhao, J., Cui, D., Wang, F., Song, Y., Cheng, L., et al. (2018). Protective effect of hydroxysafflor yellow A against acute kidney injury via the TLR4/NF-kappaB signaling pathway. *Sci. Rep.* 8 (1), 9173. doi: 10.1038/s41598-018-27217-3.

Baltaci, A. K., Gokbudak, H., Baltaci, S. B., Mogulkoc, R., and Avunduk, M. C. (2019). The effects of resveratrol administration on lipid oxidation in experimental renal ischemia-reperfusion injury in rats. *Biotech. Histochem.* 94 (8), 592-599. doi: 10.1080/10520295.2019.1612091.

Beytur, L., Korkmaz, E., Kose, E., Taslidere, A., and Tekin, S. (2025). Myrtenal pretreatment exerts a protective effect against renal ischemia-reperfusion injury in rats. *J. Biochem. Mol. Toxicol.* 39 (3), e70202. doi: 10.1002/jbt.70202.

Cai, F. F., Li, D. R., Zhou, K. Q., Zhang, W., and Yang, Y. W. (2024). Tiliroside attenuates acute kidney injury by inhibiting ferroptosis through the disruption of NRF2-KEAP1 interaction. *Phytomedicine* 126. doi: 10.1016/j.phymed.2024.155407.

Chen, D. Q., Feng, Y. L., Chen, L., Liu, J. R., Wang, M., Vaziri, N. D., et al. (2019). Poricoic acid A enhances melatonin inhibition of AKI-to-CKD transition by regulating Gas6/AxlNFkappaB/Nrf2 axis. *Free Radic. Biol. Med.* 134, 484-497. doi: 10.1016/j.freeradbiomed.2019.01.046.

Chen, H., Huang, R. S., Yu, X. X., Ye, Q., Pan, L. L., Shao, G. J., et al. (2017). Emodin protects against oxidative stress and apoptosis in HK-2 renal tubular epithelial cells after hypoxia/reoxygenation. *Exp. Ther. Med.* 14 (1), 447-452. doi: 10.3892/etm.2017.4473.

Chen, L., Yuan, J., Li, H., Ding, Y., Yang, X., Yuan, Z., et al. (2023). Trans-cinnamaldehyde attenuates renal ischemia/reperfusion injury through suppressing inflammation via JNK/p38 MAPK signaling pathway. *Int. Immunopharmacol.* 118, 110088. doi: 10.1016/j.intimp.2023.110088.

Chen, Q., Guo, J., Han, S., Wang, T., Xia, K., Yu, B., et al. (2025). Cordycepin alleviates renal ischemia-reperfusion injury by suppressing the p38/JNK signaling pathway. *Int. Immunopharmacol.* 150, 114264. doi: 10.1016/j.intimp.2025.114264.

Chiang, C. H., Lan, T. Y., Hsieh, J. H., Lin, S. C., Chen, J. W., and Chang, T. T. (2024). Diosgenin reduces acute kidney injury and ameliorates the progression to chronic kidney disease by modifying the NOX4/p65 signaling pathways. *J. Agric. Food Chem.* 72 (31), 17444-17454. doi: 10.1021/acs.jafc.4c04183.

Cui, X., Lin, L., Sun, X., Wang, L., and Shen, R. (2021). Curcumin protects against renal ischemia/reperfusion injury by regulating oxidative stress and inflammatory response. *Evid. Based Complement. Altern. Med.* 2021, 8490772-8490778. doi: 10.1155/2021/8490772.

Dai, J., Li, C., Zhao, L., Guan, C., Yang, C., Zhang, N., et al. (2022). Scutellarin protects the kidney from ischemia/reperfusion injury by targeting Nrf2. *Nephrol. Carlt.* 27 (8), 690-700. doi: 10.1111/nep.14069.

Danis, E.G., Acar, G., Dasdelen, D., Solmaz, M., Mogulkoc, R., and Baltaci, A.K. (2024). Naringin affects caspase-3, IL-1beta, and HIF-1alpha levels in experimental kidney ischemia-reperfusion in rats. *Curr. Pharm. Des.* 30 (42), 3339-3349. doi: 10.2174/0113816128324562240816095551.

Deng, G., Zheng, B., Dou, M., Gao, Y., Zhang, X., Niu, Z., et al. (2024a). Scutellarin alleviates renal ischemia-reperfusion injury by inhibiting the MAPK pathway and pro-inflammatory macrophage polarization. *FASEB J.* 38 (13), e23769. doi: 10.1096/fj.202302243R.

Deng, Y., Zeng, L., Liu, H., Zuo, A., Zhou, J., Yang, Y., et al. (2024b). Silibinin attenuates ferroptosis in acute kidney injury by targeting FTH1. *Redox Biol.* 77, 103360. doi: 10.1016/j.redox.2024.103360.

Eraslan, E., Tanyeli, A., Polat, E., and Yetim, Z. (2019). Evodiamine alleviates kidney ischemia reperfusion injury in rats: A biochemical and histopathological study. *J. Cell. Biochem.* 120 (10), 17159-17166. doi: 10.1002/jcb.28976.

Erkasap, S., Erkasap, N., Bradford, B., Mamedova, L., Uysal, O., Ozkurt, M., et al. (2017). The effect of leptin and resveratrol on JAK/STAT pathways and Sirt-1 gene expression in the renal tissue of ischemia/reperfusion induced rats. *Bratisl. Lek. Listy* 118 (8), 443-448. doi: 10.4149/BLL_2017_086.

Fan, C. R. N., Chen, Q. N., Ren, J. Y., Yang, X. H., Ru, J., Zhang, H. B., et al. (2020). Notoginsenoside R1 suppresses inflammatory signaling and rescues renal ischemia-reperfusion injury in experimental rats. *Med. Sci. Monit.* 26, e920442 doi: 10.12659/MSM.920442.

Fan, Y., Chen, H., Peng, H., Huang, F., Zhong, J., and Zhou, J. (2017). Molecular mechanisms of curcumin renoprotection in experimental acute renal injury. *Front. Pharmacol.* 8, 912. doi: 10.3389/fphar.2017.00912.

Feng, J., Kong, R. R., Xie, L. Y., Lu, W. H., Zhang, Y. L., Dong, H. J., et al. (2020). Clemaichinenoside protects renal tubular epithelial cells from hypoxia/reoxygenation injury in vitro through activating the Nrf2/HO-1 signalling pathway. *Clin. Exp. Pharmacol. Physiol.* 47 (3), 495-502. doi: 10.1111/1440-1681.13219.

Firouzeh, G., Samira, M. H., and Zeinab, K. (2025). Rosmarinic acid: a promising agent for male rats' renal protection against ischemia/reperfusion injury. *Mol. Biol. Rep.* 52 (1), 325. doi: 10.1007/s11033-025-10422-5.

Gao, D., Jing, S., Zhang, Q., and Wu, G. (2018). Pterostilbene protects against acute renal ischemia reperfusion injury and inhibits oxidative stress, inducible nitric oxide synthase expression and inflammation in rats via the Toll-like receptor 4/nuclear factor-kappaB signaling pathway. *Exp. Ther. Med.* 15 (1), 1029-1035. doi: 10.3892/etm.2017.5479.

Geng, X., Fu, Z., Geng, G., Chi, K., Liu, C., Hong, H., et al. (2024). Astilbin improves the therapeutic effects of mesenchymal stem cells in AKI-CKD mice by regulating macrophage polarization through PTGS2-mediated pathway. *Stem Cell Res. Ther.* 15 (1), 427. doi: 10.1186/s13287-024-04025-3.

Gholampour, F., Mohammadi, Z., Karimi, Z., and Owji, S. M. (2020). Protective effect of genistein in a rat model of ischemic acute kidney injury. *Gene* 753, 144789. doi: 10.1016/j.gene.2020.144789.

Gong, J., and Wang, X. (2018). Schisantherin A protects renal tubular epithelial cells from hypoxia/reoxygenation injury through the activation of PI3K/Akt signaling pathway. *J. Biochem. Mol. Toxicol*, e22160. doi: 10.1002/jbt.22160.

Guler, M. C., Akpinar, E., Tanyeli, A., Comakli, S., and Bayir, Y. (2023). Costunolide prevents renal ischemia-reperfusion injury in rats by reducing autophagy, apoptosis, inflammation, and DNA damage. *Iran. J. Basic Med. Sci.* 26 (10), 1168-1176. doi: 10.22038/IJBMS.2023.71779.15596.

Güvenç, M., Cellat, M., Uyar, A., Özkan, H., Gokcek, I., Isler, C. T., et al. (2020). Nobiletin protects from renal ischemia-reperfusion injury in rats by suppressing inflammatory cytokines and regulating iNOS-eNOS expressions. *Inflammation* 43 (1), 336-346. doi: 10.1007/s10753-019-01123-w.

Hameed Ali, R. A., Altimimi, M., and Hadi, N. R. (2023). The potential renoprotective effect of tilianin in renal ischemia reperfusion injury in male rat model. *Wiad. Lek.* 76 (12), 2657-2667. doi: 10.36740/WLek202312115.

Han, F., Dou, M., Wang, Y. X., Xu, C. X., Li, Y., Ding, X. M., et al. (2020). Cordycepin protects renal ischemia/reperfusion injury through regulating inflammation, apoptosis, and oxidative stress. *Acta Bioch. Biophys. Sin.* 52 (2), 125-132. doi: 10.1093/abbs/gmz145.

Han, L., Chen, A., Liu, L., and Wang, F. (2022). Leonurine preconditioning attenuates ischemic acute kidney injury in rats by promoting Nrf2 nuclear translocation and suppressing TLR4/NF-kappaB pathway. *Chem. Pharm. Bull. (Tokyo)* 70 (1), 66-73. doi: 10.1248/cpb.c21-00740.

He, H. Y., Shan, H. Z., Li, S. Q., and Diao, R. G. (2023). Genistein attenuates renal ischemia-reperfusion injury via ADORA2A pathway. *Hum. Exp. Toxicol.* 42, 9603271231164913. doi: 10.1177/09603271231164913.

Hong, X., Zhao, X., Wang, G., Zhang, Z., Pei, H., and Liu, Z. (2017). Luteolin treatment protects against renal ischemia-reperfusion injury in rats. *Mediat. Inflamm.* 2017, 9783893. doi: 10.1155/2017/9783893.

Hosohata, K., Jin, D., and Takai, S. (2021). Glaucocalyxin A ameliorates hypoxia/reoxygenation-induced injury in human renal proximal tubular epithelial cell line HK-2 cells. *Int. J. Mol. Sci.* 23 (1). doi: 10.3390/ijms23010446.

Hu, B., Wu, Y., Tong, F., Liu, J., Shen, X., Shen, R., et al. (2017). Apocynin alleviates renal ischemia/reperfusion injury through regulating the level of zinc and metallothionen. *Biol. Trace Elem. Res.* 178 (1), 71-78. doi: 10.1007/s12011-016-0904-z.

Huang, F., Wang, X., Xiao, G., and Xiao, J. (2022). Loganin exerts a protective effect on ischemia-reperfusion-induced acute kidney injury by regulating JAK2/STAT3 and Nrf2/HO-1 signaling pathways. *Drug Dev. Res.* 83 (1), 150-157. doi: 10.1002/ddr.21853.

Huang, Q., Shi, Z., Zheng, D., Chen, H., and Huang, Q. (2025). Shikonin inhibits endoplasmic reticulum stress-induced apoptosis to attenuate renal ischemia/reperfusion injury by activating the Sirt1/Nrf2/HO-1 pathway. *Kidney Blood Press. Res.* 50 (1), 131-146. doi: 10.1159/000542417.

Huo, A., and Wang, F. (2024). Berberine alleviates ischemia reperfusion injury induced AKI by regulation of intestinal microbiota and reducing intestinal inflammation. *BMC Complement. Med. Ther.* 24 (1), 66. doi: 10.1186/s12906-023-04323-y.

Jiang, G. P., Liao, Y. J., Huang, L. L., Zeng, X. J., and Liao, X. H. (2021). Effects and molecular mechanism of pachymic acid on ferroptosis in renal ischemia reperfusion injury. *Mol. Med. Rep.* 23 (1). doi: 10.3892/mmr.2020.11704.

Jiang, G. S., Xin, R., Yuan, W. D., Zhang, L. X., Meng, X. H., Sun, W. N., et al. (2020). Ligustrazine ameliorates acute kidney injury through downregulation of NOD2-mediated inflammation. *Int. J. Mol. Med.* 45 (3), 731-742. doi: 10.3892/ijmm.2020.4464.

Kalbolandi, S. M., Gorji, A. V., Babaahmadi-Rezaei, H., and Mansouri, E. (2019). Luteolin confers renoprotection against ischemia-reperfusion injury via involving Nrf2 pathway and regulating miR320. *Mol. Biol. Rep.* 46 (4), 4039-4047. doi: 10.1007/s11033-019-04853-0.

Kapisiz, A., Kaya, C., Eryilmaz, S., Karabulut, R., Turkyilmaz, Z., Inan, M. A., et al. (2024). Protective effects of lupeol in rats with renal ischemia‑reperfusion injury. *Exp. Ther. Med.* 28 (2), 313. doi: 10.3892/etm.2024.12602.

Kar, F., Hacioglu, C., Senturk, H., Donmez, D.B., Kanbak, G., and Uslu, S. (2020). Curcumin and LOXblock-1 ameliorate ischemia-reperfusion induced inflammation and acute kidney injury by suppressing the semaphorin-plexin pathway. *Life Sci.* 256, 118016. doi: 10.1016/j.lfs.2020.118016.

Karacaer, C., Erdogan, D. G., Tanyeli, A., Baylan, H., Eraslan, E., Guler, M. C., et al. (2023). The protective effects of hesperidin pretreatment on kidney and remote organs against renal ischemia-reperfusion injury. *Eur. Rev. Med. Pharmacol. Sci.* 27 (7), 2808-2814. doi: 10.26355/eurrev_202304_31911.

Kaur, A., Kaur, T., Singh, B., Pathak, D., Singh Buttar, H., and Pal Singh, A. (2016). Curcumin alleviates ischemia reperfusion-induced acute kidney injury through NMDA receptor antagonism in rats. *Ren. Fail.* 38 (9), 1462-1467. doi: 10.1080/0886022X.2016.1214892.

Kisaoglu, A., Kose, E., Yilmaz, N., Tanbek, K., Yildiz, A., Yilmaz, U., et al. (2024). Investigation of the effect of astaxanthin on autophagy in renal ischemia-reperfusion modeled rats. *Medeni. Med. J.* 39 (2), 101-108. doi: 10.4274/MMJ.galenos.2024.27243.

Kuang, B. C., Wang, Z. H., Hou, S. H., Zhang, J., Wang, M. Q., Zhang, J. S., et al. (2023). Methyl eugenol protects the kidney from oxidative damage in mice by blocking the Nrf2 nuclear export signal through activation of the AMPK/GSK3beta axis. *Acta Pharmacol. Sin.* 44 (2), 367-380. doi: 10.1038/s41401-022-00942-2.

Kyriakopoulos, G., Tsaroucha, A. K., Valsami, G., Lambropoulou, M., Kostomitsopoulos, N., Christodoulou, E., et al. (2018). Silibinin improves TNF-alpha and M30 expression and histological parameters in rat kidneys after hepatic ischemia/reperfusion. *J. Invest. Surg.* 31 (3), 201-209. doi: 10.1080/08941939.2017.1308044.

Li, C., Zheng, Z., Xie, Y., Zhu, N., Bao, J., Yu, Q., et al. (2020). Protective effect of taraxasterol on ischemia/reperfusion-induced acute kidney injury via inhibition of oxidative stress, inflammation, and apoptosis. *Int. Immunopharmacol.* 89 (Pt A), 107169. doi: 10.1016/j.intimp.2020.107169.

Li, H., Chen, W., Chen, Y., Zhou, Q., Xiao, P., Tang, R., et al. (2019). Neferine attenuates acute kidney injury by inhibiting NF-kappaB signaling and upregulating klotho expression. *Front. Pharmacol.* 10, 1197. doi: 10.3389/fphar.2019.01197.

Li, J., Li, L., Wang, S., Zhang, C., Zheng, L., Jia, Y., et al. (2018). Resveratrol alleviates inflammatory responses and oxidative stress in rat kidney ischemia-reperfusion injury and H2O2-induced NRK-52E cells via the Nrf2/TLR4/NF-kappaB pathway. *Cell. Physiol. Biochem.* 45 (4), 1677-1689. doi: 10.1159/000487735.

Li, K., Gong, X., Kuang, G., Jiang, R., Wan, J., and Wang, B. (2016). Sesamin protects against renal ischemia reperfusion injury by promoting CD39-adenosine-A2AR signal pathway in mice. *Am. J. Transl. Res.* 8 (5), 2245-2254.

Li, L., Liu, S., Zhou, Y., Zhao, M., Wang, Y., Wang, C., et al. (2021a). Indispensable role of mitochondria in maintaining the therapeutic potential of curcumin in acute kidney injury. *J. Cell. Mol. Med.* 25 (20), 9863-9877. doi: 10.1111/jcmm.16934.

Li, M., Ning, J., Huang, H., Jiang, S., and Zhuo, D. (2022). Allicin protects against renal ischemia-reperfusion injury by attenuating oxidative stress and apoptosis. *Int. Urol. Nephrol.* 54 (7), 1761-1768. doi: 10.1007/s11255-021-03014-2.

Li, W.F., Yang, K., Zhu, P., Zhao, H.Q., Song, Y.H., Liu, K.C., et al. (2017). Genistein ameliorates ischemia/reperfusion-induced renal injury in a SIRT1-dependent manner. *Nutrients* 9 (4). doi: 10.3390/nu9040403.

Li, Z., Zhu, J., Wan, Z., Li, G., Chen, L., and Guo, Y. (2021b). Theaflavin ameliorates renal ischemia/reperfusion injury by activating the Nrf2 signalling pathway in vivo and in vitro. *Biomed. Pharmacother.* 134, 111097. doi: 10.1016/j.biopha.2020.111097.

Liang, S., Xu, Z., Ruan, Y., Niu, T., Guo, W., Jiang, W., et al. (2020). Isoquercitrin attenuates renal ischemia/reperfusion injury through antioxidation, anti-inflammation, and antiapoptosis in mice. *Transplant. Proc.* 52 (3), 1014-1019. doi: 10.1016/j.transproceed.2019.12.038.

Liao, Y., Lin, X., Li, J., Tan, R., Zhong, X., and Wang, L. (2021). Nodakenin alleviates renal ischaemia-reperfusion injury via inhibiting reactive oxygen species-induced NLRP3 inflammasome activation. *Nephrology (Carlton)* 26 (1), 78-87. doi: 10.1111/nep.13781.

Lin, Y., Sheng, M., Ding, Y., Zhang, N., Song, Y., Du, H., et al. (2018). Berberine protects renal tubular cells against hypoxia/reoxygenation injury via the Sirt1/p53 pathway. *J. Nat. Med.* 72 (3), 715-723. doi: 10.1007/s11418-018-1210-1.

Liu, B., Deng, Q., Zhang, L., and Zhu, W. (2020a). Nobiletin alleviates ischemia/reperfusion injury in the kidney by activating PI3K/AKT pathway. *Mol. Med. Rep.* 22 (6), 4655-4662. doi: 10.3892/mmr.2020.11554.

Liu, D., Zhang, C., Hu, M., and Su, K. (2021). Scutellarein relieves the death and inflammation of tubular epithelial cells in ischemic kidney injury by degradation of COX-2 protein. *Int. Immunopharmacol.* 101 (Pt A), 108193. doi: 10.1016/j.intimp.2021.108193.

Liu, F., Ni, W., Zhang, J., Wang, G., Li, F., and Ren, W. (2017a). Administration of curcumin protects kidney tubules against renal ischemia-reperfusion injury (RIRI) by modulating nitric oxide (NO) signaling pathway. *Cell. Physiol. Biochem.* 44 (1), 401-411. doi: 10.1159/000484920.

Liu, F. H., Ni, W. J., Wang, G. K., and Zhang, J. J. (2016). Protective role of curcumin on renal ischemia reperfusion injury via attenuating the inflammatory mediators and Caspase-3. *Cell. Mol. Biol. (Noisy-le-grand)* 62 (11), 95-99.

Liu, J., Sun, X., Liang, J., and Song, S. (2025a). Eugenol alleviates renal ischemia-reperfusion injury induced-endoplasmic reticulum stress via activating Sestrin2. *Clinics (Sao Paulo)* 80, 100627. doi: 10.1016/j.clinsp.2025.100627.

Liu, Q., Liang, X. B., Liang, M. T., Qin, R. B., Qin, F. X., and Wang, X. L. (2020b). Ellagic acid ameliorates renal ischemic-reperfusion injury through NOX4/JAK/STAT signaling pathway. *Inflammation* 43 (1), 298-309. doi: 10.1007/s10753-019-01120-z.

Liu, X., Ning, K. H., Li, J. C., Yan, X. D., Yan, J. H., Zhao, X. Y., et al. (2025b). Sodium aescinate protects renal ischemia-reperfusion and pyroptosis through AKT/NLRP3 signaling pathway. *Ren. Fail.* 47 (1). doi: 10.1080/0886022x.2025.2488140.

Liu, Y., Liu, X., Wang, L., Du, Y., Chen, Z., Chen, H., et al. (2017b). Effects of apigenin on the expression levels of B-cell lymphoma-2, Fas and Fas ligand in renal ischemia-reperfusion injury in rats. *Exp. Ther. Med.* 14 (6), 5345-5354. doi: 10.3892/etm.2017.5241.

Liu, Y., Wang, L., Du, Y., Chen, Z., Guo, J., Weng, X., et al. (2017c). Effects of apigenin pretreatment against renal ischemia/reperfusion injury via activation of the JAK2/STAT3 pathway. *Biomed. Pharmacother.* 95, 1799-1808. doi: 10.1016/j.biopha.2017.09.091.

Liu, Z., Guan, C., Li, C., Zhang, N., Yang, C., Xu, L., et al. (2022). Tilianin reduces apoptosis via the ERK/EGR1/BCL2L1 pathway in ischemia/reperfusion-induced acute kidney injury mice. *Front. Pharmacol.* 13, 862584. doi: 10.3389/fphar.2022.862584.

Lu, H., Xie, D., Qu, B., Li, M., He, Y., and Liu, W. (2023). Emodin prevents renal ischemia-reperfusion injury via suppression of p53-mediated cell apoptosis based on network pharmacology. *Heliyon* 9 (5), e15682. doi: 10.1016/j.heliyon.2023.e15682.

Lu, J., Yi, Y., Pan, R., Zhang, C., Han, H., Chen, J., et al. (2018). Berberine protects HK-2 cells from hypoxia/reoxygenation induced apoptosis via inhibiting SPHK1 expression. *J. Nat. Med.* 72 (2), 390-398. doi: 10.1007/s11418-017-1152-z.

Ma, L., Liu, X., Zhang, M., Zhou, L., Jiang, L., Gao, L., et al. (2023). Paeoniflorin alleviates ischemia/reperfusion induced acute kidney injury by inhibiting Slc7a11-mediated ferroptosis. *Int. Immunopharmacol.* 116, 109754. doi: 10.1016/j.intimp.2023.109754.

Machado, D. I., de Oliveira Silva, E., Ventura, S., and Vattimo, M. F. F. (2022). The effect of curcumin on renal ischemia/reperfusion injury in diabetic rats. *Nutrients* 14 (14). doi: 10.3390/nu14142798.

Mao, H., Wang, L., Xiong, Y., Jiang, G., and Liu, X. (2022). Fucoxanthin attenuates oxidative damage by activating the Sirt1/Nrf2/HO-1 signaling pathway to protect the kidney from ischemia-reperfusion injury. *Oxid. Med. Cell. Longev.* 2022, 7444430. doi: 10.1155/2022/7444430.

Martinez-Rojas, M. A., Balcazar, H., Ponce-Nava, M. S., Gonzalez-Soria, I., Marquina-Castillo, B., Perez-Villalva, R., et al. (2024). A short treatment with resveratrol after a renal ischaemia-reperfusion injury prevents maladaptive repair and long-term chronic kidney disease in rats. *J. Physiol.* 602 (8), 1835-1852. doi: 10.1113/JP285979.

Meng, X., Wei, M., Wang, D., Qu, X., Zhang, K., Zhang, N., et al. (2020). The protective effect of hesperidin against renal ischemia-reperfusion injury involves the TLR-4/NF-kappaB/iNOS pathway in rats. *Physiol. Int.* 107 (1), 82-91. doi: 10.1556/2060.2020.00003.

Mohamed, M. E., Elmorsy, M. A., and Younis, N. S. (2022a). Renal ischemia/reperfusion mitigation via geraniol: The role of Nrf-2/HO-1/NQO-1 and TLR2,4/MYD88/NFkappaB pathway. *Antioxidants (Basel)* 11(8). doi: 10.3390/antiox11081568.

Mohamed, M. E., Kandeel, M., Abd El-Lateef, H. M., El-Beltagi, H. S., and Younis, N. S. (2022b). The protective effect of anethole against renal ischemia/reperfusion: the role of the TLR2,4/MYD88/NFkappaB pathway. *Antioxidants (Basel)* 11 (3). doi: 10.3390/antiox11030535.

Mohammadi, M., Najafi, H., Mohamadi Yarijani, Z., Vaezi, G., and Hojati, V. (2020). Protective effect of piperine in ischemia-reperfusion induced acute kidney injury through inhibition of inflammation and oxidative stress. *J. Tradit. Complement. Med.* 10 (6), 570-576. doi: 10.1016/j.jtcme.2019.07.002.

Nasrallah, H., Aissa, I., Slim, C., Boujbiha, M. A., Zaouali, M. A., Bejaoui, M., et al. (2020). Effect of oleuropein on oxidative stress, inflammation and apoptosis induced by ischemia-reperfusion injury in rat kidney. *Life Sci.* 255, 117833. doi: 10.1016/j.lfs.2020.117833.

Ozturk, H., Cetinkaya, A., Yilmaz, F., and Ozturk, H. (2017). Protective effect of oxymatrine against renal ischemia/reperfusion injury in rats. *Bratisl. Lek. Listy.* 118 (4), 217-222. doi: 10.4149/BLL_2017_043.

Pan, X., Zhu, R., Peng, J., Liu, H., Pan, W., Jin, Y., et al. (2024). Molecular mechanisms and potential targets of lycopene for alleviating renal ischemia-reperfusion injury revealed by network pharmacology and animal experiments. *Int. Immunopharmacol.* 143 (Pt 2), 113421. doi: 10.1016/j.intimp.2024.113421.

Pang, Y., Zhang, P.C., Lu, R. R., Li, H. L., Li, J. C., Fu, H. X., et al. (2020). Andrade-oliveira salvianolic acid B modulates caspase-1-mediated pyroptosis in renal ischemia-reperfusion injury via Nrf2 pathway. *Front. Pharmacol.* 11, 541426. doi: 10.3389/fphar.2020.541426.

Park, E. J., Dusabimana, T., Je, J., Jeong, K., Yun, S. P., Kim, H. J., et al. (2020). Honokiol protects the kidney from renal ischemia and reperfusion injury by upregulating the glutathione biosynthetic enzymes. *Biomedicines* 8(9). doi: 10.3390/biomedicines8090352.

Park, W. S., Park, M. S., Kang, S. W., Jin, S. A., Jeon, Y., Hwang, J., et al. (2019). Hesperidin shows protective effects on renal function in ischemia-induced acute kidney injury (sprague-dawley rats). *Transplant. Proc.* 51(8), 2838-2841. doi: 10.1016/j.transproceed.2019.02.055.

Peng, J., Ren, X., Lan, T., Chen, Y., Shao, Z., and Yang, C. (2016). Renoprotective effects of ursolic acid on ischemia/reperfusion‑induced acute kidney injury through oxidative stress, inflammation and the inhibition of STAT3 and NF‑kappaB activities. *Mol. Med. Rep.* 14 (4), 3397-3402. doi: 10.3892/mmr.2016.5654.

Prem, P. N., and Kurian, G. A. (2022). Fisetin attenuates renal ischemia/reperfusion injury by improving mitochondrial quality, reducing apoptosis and oxidative stress. *Naunyn Schmiedebergs Arch. Pharmacol.* 395 (5), 547-561. doi: 10.1007/s00210-022-02204-8.

Qi, Y., Zheng, J., Zi, Y., Song, W., Chen, X., Cao, S., et al. (2024). Loureirin C improves mitochondrial function by promoting NRF2 nuclear translocation to attenuate oxidative damage caused by renal ischemia-reperfusion injury. *Int. Immunopharmacol.* 138, 112596. doi: 10.1016/j.intimp.2024.112596.

Ren, K., Jin, C., Ma, P., Ren, Q., Jia, Z., and Zhu, D. (2016). Ginsenoside Rd alleviates mouse acute renal ischemia/reperfusion injury by modulating macrophage phenotype. *J. Ginseng Res.* 40 (2), 196-202. doi: 10.1016/j.jgr.2015.12.003.

Ren, L., Zhao, Y., Ji, X., Li, W., Jiang, W., Li, Q., et al. (2024). The therapeutic effect of Picroside II in renal ischemia-reperfusion induced acute kidney injury: An experimental study. *Eur. J. Pharmacol.* 967, 176391. doi: 10.1016/j.ejphar.2024.176391.

Sallabi, S. M., Lubbad, L., Toumi, H. R., Hammad, W. F., Sudhadevi, M., Rasheed, J. A., et al. (2025). The effect of nuciferine on the renal dysfunction following ischemia-reperfusion injury. *PLoS One* 20 (3), e0320320. doi: 10.1371/journal.pone.0320320.

Sancak, E. B., Akbas, A., Silan, C., Cakir, D. U., Turkon, H., and Ozkanli, S. S. (2016). Protective effect of syringic acid on kidney ischemia-reperfusion injury. *Ren. Fail.* 38 (4), 629-635. doi: 10.3109/0886022X.2016.1149868.

Shan, R. R., Yu, J. T., Zhang, S. F., Xie, M. M., Hou, R., Xie, C. Y., et al. (2024). Madecassoside alleviates acute kidney injury by regulating JNK-mediated oxidative stress and programmed cell death. *Phytomedicine* 123, 155252. doi: 10.1016/j.phymed.2023.155252.

Shan, Y., Chen, D., Hu, B., Xu, G., Li, W., Jin, Y., et al. (2021). Allicin ameliorates renal ischemia/reperfusion injury via inhibition of oxidative stress and inflammation in rats. *Biomed. Pharmacother.* 142, 112077. doi: 10.1016/j.biopha.2021.112077.

Shao, G., He, J., Meng, J., Ma, A., Geng, X., Zhang, S., et al. (2021). Ganoderic acids prevent renal ischemia reperfusion injury by inhibiting inflammation and apoptosis. *Int. J. Mol. Sci.* 22(19). doi: 10.3390/ijms221910229.

Shao, Y. F., Tang, B. B., Ding, Y. H., Fang, C. Y., Hong, L., Shao, C. X., et al. (2023). Kaempferide ameliorates cisplatin-induced nephrotoxicity via inhibiting oxidative stress and inducing autophagy. *Acta Pharmacol. Sin.* 44 (7), 1442-1454. doi: 10.1038/s41401-023-01051-4.

Su, Y., Xu, J., Chen, S., Feng, J., Li, J., Lei, Z., et al. (2022). Astragaloside IV protects against ischemia/reperfusion (I/R)-induced kidney injury based on the Keap1-Nrf2/ARE signaling pathway. *Transl. Androl. Urol.* 11 (8), 1177-1188. doi: 10.21037/tau-22-505.

Sun, W., Li, A., Wang, Z., Sun, X., Dong, M., Qi, F., et al. (2020). Tetramethylpyrazine alleviates acute kidney injury by inhibiting NLRP3/HIF‑1alpha and apoptosis. *Mol. Med. Rep.* 22 (4), 2655-2664. doi: 10.3892/mmr.2020.11378.

Sun, Y., Xun, L., Jin, G., and Shi, L. (2018). Salidroside protects renal tubular epithelial cells from hypoxia/reoxygenation injury in vitro. *J. Pharmacol. Sci.* 137 (2), 170-176. doi: 10.1016/j.jphs.2018.05.011.

Tang, Z., Feng, Y., Nie, W., and Li, C. (2023a). Xanthohumol attenuates renal ischemia/reperfusion injury by inhibiting ferroptosis. *Exp. Ther. Med.* 26 (6), 571. doi: 10.3892/etm.2023.12269.

Tang, Z., Wang, Y., Liu, Y., and Li, C. (2023b). Salidroside inhibits renal ischemia/reperfusion injury‑induced ferroptosis by the PI3K/AKT signaling pathway. *Exp. Ther. Med.* 26 (5), 507. doi: 10.3892/etm.2023.12206.

Toprak, T., Sekerci, C. A., Aydin, H. R., Ramazanoglu, M. A., Arslan, F. D., Basok, B. I., et al. (2020). Protective effect of chlorogenic acid on renal ischemia/reperfusion injury in rats. *Arch. Ital. Urol. Androl.* 92 (2). doi: 10.4081/aiua.2020.2.153.

Wang, C., Hao, Z., Zhou, J., Zhang, L., Sun, Y., and Liang, C. (2017). Rutaecarpine alleviates renal ischemia reperfusion injury in rats by suppressing the JNK/p38 MAPK signaling pathway and interfering with the oxidative stress response. *Mol. Med. Rep.* 16 (1), 922-928. doi: 10.3892/mmr.2017.6631.

Wang, J., Yu, Y., Zhang, H., Li, L., Wang, J., Su, S., et al. (2024). Gypenoside XVII attenuates renal ischemia-reperfusion injury by inhibiting endoplasmic reticulum stress and NLRP3 inflammasome-triggered pyroptosis. *Eur. J. Pharmacol.* 962, 176187. doi: 10.1016/j.ejphar.2023.176187.

Wang, J., Zheng, Q., Chen, Z., Liu, X., Wan, S., and Wang, L. (2025). Puerarin alleviates renal ischemia/reperfusion injury by inhibiting apoptosis and endoplasmic reticulum stress via Nrf2/HO-1 pathway. *Iran. J. Basic Med. Sci.* 28 (2), 187-193. doi: 10.22038/ijbms.2024.80438.17412.

Wang, M., Weng, X., Chen, H., Chen, Z., and Liu, X. (2020a). Resveratrol inhibits TNF-alpha-induced inflammation to protect against renal ischemia/reperfusion injury in diabetic rats. *Acta Cir. Bras.* 35 (5), e202000506. doi: 10.1590/s0102-865020200050000006.

Wang, X., Wang, W., Wang, J.Z., Yang, C., and Liang, C.Z. (2018). Effect of apigenin on apoptosis induced by renal ischemia/reperfusion injury in vivo and in vitro. *Ren. Fail.* 40 (1), 498-505. doi: 10.1080/0886022X.2018.1497517.

Wang, Y., Han, K., Li, Z., Tang, X., Wang, C., Zhao, Y., et al. (2022a). Protective effect of hydroxysafflor yellow A on renal ischemia‑-reperfusion injury by targeting the Akt‑Nrf2 axis in mice. *Exp. Ther. Med.* 24 (6), 741. doi: 10.3892/etm.2022.11677.

Wang, Y., Liu, Q., Cai, J., Wu, P., Wang, D., Shi, Y., et al. (2022b). Emodin prevents renal ischemia-reperfusion injury via suppression of CAMKII/DRP1-mediated mitochondrial fission. *Eur. J. Pharmacol.* 916, 174603. doi: 10.1016/j.ejphar.2021.174603.

Wang, Y., Quan, F., Cao, Q., Lin, Y., Yue, C., Bi, R., et al. (2021). Quercetin alleviates acute kidney injury by inhibiting ferroptosis. *J. Adv. Res.* 28, 231-243. doi: 10.1016/j.jare.2020.07.007.

Wang, Z. S., Shu, B., Han, Q., Li, G. H., and Guo, Y. L. (2023). Effects of grape seed-derived proanthocyanidin B2 pretreatment on oxidative stress, endoplasmic reticulum stress and apoptosis of renal tubular epithelial cells in renal ischemia-reperfusion injury model of mice. *Int. Urol. Nephrol.* 55 (10), 2599-2610. doi: 10.1007/s11255-023-03494-4.

Wang, Z. S., Zhou, H. H., Han, Q., Guo, Y. L., and Li, Z. Y. (2020b). Effects of grape seed proanthocyanidin B2 pretreatment on oxidative stress and renal tubular epithelial cell apoptosis after renal ischemia reperfusion in mice. *Acta Cir. Bras.* 35 (8), e202000802. doi: 10.1590/s0102-865020200080000002.

Wei, J. P., Zhao, B., Jiang, Z. J., Wang, P. Y., Xu, Y., Ding, N., et al. (2025). Luteolin mitigates renal ischemia-reperfusion injury via anti-inflammatory, anti-apoptotic, and Nrf2/HO-1-mediated antioxidant effects. *Eur. J. Pharmacol.* 999, 177676. doi: 10.1016/j.ejphar.2025.177676.

Wu, L., Li, Q., Liu, S., An, X., Huang, Z., Zhang, B., et al. (2019). Protective effect of hyperoside against renal ischemia-reperfusion injury via modulating mitochondrial fission, oxidative stress, and apoptosis. *Free Radic. Res.* 53 (7), 727-736. doi: 10.1080/10715762.2019.1623883.

Xia, K., Jin, Z., Qiu, Q., Zhou, Y., Lu, Y., Qiu, T., et al. (2024). Ligustilide alleviates oxidative stress during renal ischemia-reperfusion injury through maintaining Sirt3-dependent mitochondrial homeostasis. *Phytomedicine* 134, 155975. doi: 10.1016/j.phymed.2024.155975.

Xiao, Z., and Vijayalakshmi, A. (2022). Protective effect of piperlongumine on inflammation and oxidative stress against ischemia-reperfusion injury in animal kidney. *Bratisl. Lek. Listy.* 123 (12), 878-884. doi: 10.4149/BLL_2022_140.

Xiong, Y., Jian, J., Yu, H., Wu, J., Mao, H., Feng, R., et al. (2023). Cyanidin-3-O-glucoside plays a protective role against renal ischemia/ reperfusion injury via the JAK/STAT pathway. *Acta Cir. Bras.* 38, e381023. doi: 10.1590/acb381023.

Xu, C., Wang, H., Wang, H., Man, J., Deng, Y., Li, Y., et al. (2025). Schisandrin B regulates mitochondrial dynamics via AKT1 activation and mitochondrial targeting to ameliorate renal ischemia-reperfusion injury. *Phytomedicine* 141, 156672. doi: 10.1016/j.phymed.2025.156672.

Xu, C. H., Deng, Y., Man, J. W., Wang, H. B., Che, T. J., Ding, L. Y., et al. (2024). Unveiling the renoprotective mechanisms of schisandrin B in ischemia-reperfusion injury through transcriptomic and pharmacological analysis. *Drug Des. Dev. Ther.* 18, 4241-4256. doi: 10.2147/Dddt.S489458.

Xu, J., Ma, L., and Fu, P. (2021). Eriocitrin attenuates ischemia reperfusion-induced oxidative stress and inflammation in rats with acute kidney injury by regulating the dual-specificity phosphatase 14 (DUSP14)-mediated Nrf2 and nuclear factor-kappaB (NF-kappaB) pathways. *Ann. Transl. Med.* 9 (4), 350. doi: 10.21037/atm-21-337.

Xu, M. W., Shi, H. B., and Liu, D. C. (2019). Chrysin protects against renal ischemia reperfusion induced tubular cell apoptosis and inflammation in mice. *Exp. Ther. Med.* 17 (3), 2256-2262. doi: 10.3892/etm.2019.7189.

Xu, Y., Wang, W., Jin, K., Zhu, Q., Lin, H., Xie, M., et al. (2017). Perillyl alcohol protects human renal tubular epithelial cells from hypoxia/reoxygenation injury via inhibition of ROS, endoplasmic reticulum stress and activation of PI3K/Akt/eNOS pathway. *Biomed. Pharmacother.* 95, 662-669. doi: 10.1016/j.biopha.2017.08.129.

Xu, Y. M., Ding, G. H., Huang, J., and Xiong, Y. (2016). Tanshinone IIA pretreatment attenuates ischemia/reperfusion-induced renal injury. *Exp. Ther. Med.* 12 (4), 2741-2746. doi: 10.3892/etm.2016.3674.

Xue, L., Jiang, S., and Wan, X.Y. (2024). Protective effects of sesamol on renal ischemia-reperfusion injury via regulation of nuclear factor erythroid 2-related factor 2 pathway. *Transplant. Proc.* 56 (2), 290-296. doi: 10.1016/j.transproceed.2023.12.009.

Yang, K., Li, W. F., Yu, J. F., Yi, C., and Huang, W. F. (2017). Diosmetin protects against ischemia/reperfusion-induced acute kidney injury in mice. *J. Surg. Res.* 214, 69-78. doi: 10.1016/j.jss.2017.02.067.

Yang, Q., Zang, H. M., Xing, T., Zhang, S. F., Li, C., Zhang, Y., et al. (2021). Gypenoside XLIX protects against acute kidney injury by suppressing IGFBP7/IGF1R-mediated programmed cell death and inflammation. *Phytomedicine* 85, 153541. doi: 10.1016/j.phymed.2021.153541.

Yao, M., Qin, S., Xiong, J., Xin, W., Guan, X., Gong, S., et al. (2022). Oroxylin A ameliorates AKI-to-CKD transition through maintaining PPARalpha-BNIP3 signaling-mediated mitochondrial homeostasis. *Front. Pharmacol.* 13, 935937. doi: 10.3389/fphar.2022.935937.

Yarijani, Z. M., Pourmotabbed, A., Pourmotabbed, T., and Najafi, H. (2017). Crocin has anti-inflammatory and protective effects in ischemia-reperfusion induced renal injuries. *Iran. J. Basic Med. Sci.* 20 (7), 753-759. doi: 10.22038/IJBMS.2017.9005.

Ye, Q., Zhu, Y.I., Ye, S., Liu, H., She, X., Niu, Y., et al. (2016). Gypenoside attenuates renal ischemia/reperfusion injury in mice by inhibition of ERK signaling. *Exp. Ther. Med.* 11 (4), 1499-1505. doi: 10.3892/etm.2016.3034.

Yin, S., Liu, J., Zhao, X., Dong, H., Cao, Y., Zhang, S., et al. (2023). Chitosan oligosaccharide attenuates acute kidney injury and renal interstitial fibrosis induced by ischemia-reperfusion. *Ren. Fail.* 45 (1), 2238831. doi: 10.1080/0886022X.2023.2238831.

Younis, N.S., and Ghanim, A.M.H. (2022). The protective role of celastrol in renal ischemia-reperfusion injury by activating Nrf2/HO-1, PI3K/AKT signaling pathways, modulating NF-kappab signaling pathways, and inhibiting ERK phosphorylation. *Cell Biochem. Biophys.* 80 (1), 191-202. doi: 10.1007/s12013-022-01064-6.

Zhang, B., Chen, Z. Y., Jiang, Z., Huang, S., Liu, X. H., and Wang, L. (2023). Nephroprotective effects of cardamonin on renal ischemia reperfusion injury/UUO-induced renal fibrosis. *J. Agric. Food Chem.* 71 (36), 13284-13303. doi: 10.1021/acs.jafc.3c01880.

Zhang, B., Wan, S., Liu, H., Qiu, Q., Chen, H., Chen, Z., et al. (2022a). Naringenin alleviates renal ischemia reperfusion injury by suppressing ER stress-induced pyroptosis and apoptosis through activating Nrf2/HO-1 signaling pathway. *Oxid. Med. Cell. Longev.* 2022, 5992436. doi: 10.1155/2022/5992436.

Zhang, J., Tang, L., Li, G. S., and Wang, J. (2018a). The anti-inflammatory effects of curcumin on renal ischemia-reperfusion injury in rats. *Ren. Fail.* 40(1), 680-686. doi: 10.1080/0886022X.2018.1544565.

Zhang, N., Guan, C., Liu, Z., Li, C., Yang, C., Xu, L., et al. (2022b). Calycosin attenuates renal ischemia/reperfusion injury by suppressing NF-kappaB mediated inflammation via PPARgamma/EGR1 pathway. *Front. Pharmacol.* 13, 970616. doi: 10.3389/fphar.2022.970616.

Zhang, S., Xu, X., Huang, Y., Sun, S., Jin, C., Ji, H., et al. (2021). Anisodamine ameliorates ischemia/reperfusion-induced renal injury in rats through activation of the extracellular signal-regulated kinase (ERK) pathway and anti-apoptotic effect. *Pharmazie* 76 (5), 220-224. doi: 10.1691/ph.2021.1302.

Zhang, Y., Liu, M., Zhang, Y., Tian, M., Chen, P., Lan, Y., et al. (2022c). Urolithin A alleviates acute kidney injury induced by renal ischemia reperfusion through the p62-Keap1-Nrf2 signaling pathway. *Phytother. Res.* 36 (2), 984-995. doi: 10.1002/ptr.7370.

Zhang, Z., Qi, D., Wang, X., Gao, Z., Li, P., Liu, W., et al. (2018b). Protective effect of Salvianolic acid A on ischaemia-reperfusion acute kidney injury in rats through protecting against peritubular capillary endothelium damages. *Phytother. Res.* 32 (1), 103-114. doi: 10.1002/ptr.5954.

Zheng, Y., Zhang, N., and Bai, F. (2022). Gastrodin pretreatment alleviates renal ischemia-reperfusion injury. *Urol. Int.* 106 (6), 630-637. doi: 10.1159/000520531.

Zheng, Y., Zhang, Y., Zheng, Y., and Zhang, N. (2018). Carnosol protects against renal ischemia-reperfusion injury in rats. *Exp. Anim.* 67 (4), 545-553. doi: 10.1538/expanim.18-0067.

Zhou, Q., Gong, X., Kuang, G., Jiang, R., Xie, T., Tie, H., et al. (2018). Ferulic acid protected from kidney ischemia reperfusion injury in mice: possible mechanism through increasing adenosine generation via HIF-1alpha. *Inflammation* 41 (6), 2068-2078. doi: 10.1007/s10753-018-0850-3.

Zhu, J., Shen, H., Li, G., Chen, L., Kang, P., Guo, Y., et al. (2024). Theaflavin pretreatment ameliorates renal ischemia/reperfusion injury by attenuating apoptosis and oxidative stress in vivo and in vitro. *Biomed. Pharmacother.* 171, 116114. doi: 10.1016/j.biopha.2023.116114.

Zhu, Y. L., Huang, J., Chen, X. Y., Xie, J., Yang, Q., Wang, J. F., et al. (2022). Senkyunolide I alleviates renal ischemia-reperfusion injury by inhibiting oxidative stress, endoplasmic reticulum stress and apoptosis. *Int. Immunopharmacol.* 102, 108393. doi: 10.1016/j.intimp.2021.108393.
